# Engineered *E. coli* swarming for binary and analog input recording

**DOI:** 10.1038/s44320-026-00232-7

**Published:** 2026-07-16

**Authors:** Marian Shaw, Sadhya Garg, Julia Kirby, Samuel Schmidgall, Filippo Liguori, Anjali Doshi, Tao Tu, Tal Danino

**Affiliations:** 1 Department of Biomedical Engineering, Columbia University, New York, NY USA; 2 Google DeepMind, Mountain View, CA USA; 3 Herbert Irving Comprehensive Cancer Center, Columbia University, New York, NY USA; 4 Data Science Institute, Columbia University, New York, NY USA

**Keywords:** Microbiology, Virology & Host Pathogen Interaction

## Abstract

Many bacterial species form self-organized macroscale patterns through swarming. Despite its extensive genetic tractability, *Escherichia coli* remains underexplored for robust, applied control of swarming. Here we develop a set of *E. coli* strains that generate centimeter-scale swarming patterns to spatially record environmental inputs. Specifically, we modulate the expression of swarming-related genes in response to chemical and optical signals, reshaping baseline swarm patterns in analog or binary-like fashions. To decode bacterial patterns across space and time, we develop scalable computational methods incorporating feature extraction, regression, and deep-learning models. Time-lapse imaging reveals that colonies record inputs dynamically, enabling early-stage classification. This work establishes a strategy for spatial information recording in *E. coli* and expands the toolkit for programming emergent microbial behaviors at macroscopic scales.

## Synopsis

Swarming bacteria form structured, macroscale colony patterns that reflect local conditions. Here, engineered swarming *E. coli* recorded predefined inputs in distinct spatial patterns, enabling visual readout and quantitative decoding using computational analysis.

- Colony patterns were modulated across chemical (IPTG) and optical (blue light) input ranges, exhibiting switch-like and quasi-continuous responses.
- Patterns were quantified using interpretable image-processing features and decoded with regression and deep-learning models, achieving high performance (AUC 0.89–1.00) across strains and input levels.
- Time-resolved features enabled early decoding (~ 5 h) and captured transient input perturbations, demonstrating dynamic spatiotemporal recording during colony expansion.

## Abstract (web summary)

Swarming bacteria form structured, macroscale colony patterns that reflect local conditions. Here, engineered swarming *E. coli* recorded predefined inputs in distinct spatial patterns, enabling visual readout and quantitative decoding using computational analysis.

## Graphical abstract

**Figure:**
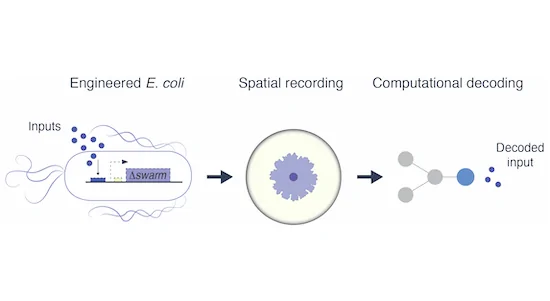

**Figure:**
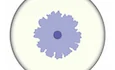

## Introduction

Across nature, coordinated collective behaviors emerge as individuals sense local cues and integrate their responses, often yielding striking self-organized patterns exemplified by starling murmurations, fish schools, and microbial swarms (Sumpter, 2006; Camazine et al, 2020). In bacteria, swarming is a collective surface-based motility that enhances nutrient access, colonization, and stress survival. Expansive and highly coordinated, swarming arises through environmentally responsive physiological changes—including cell elongation, hyperflagellation, and intercellular interactions—that generate macroscale morphologies such as concentric circles, branching networks, or spiraling vortices (Kearns, 2010). As colonies expand across surfaces, these emergent morphologies integrate cellular responses over space and time, effectively serving as visible records of local environmental conditions. The prospect of harnessing natural self-organizing behaviors has long inspired efforts to engineer adaptive systems that illuminate principles of coordination and enable innovations in medicine, materials fabrication, and beyond (Rubenstein et al, 2014; Slavkov et al, 2018; Johnson et al, 2017; Santos-Moreno and Schaerli, 2019; Das et al, 2025).

Over the past two decades, synthetic biology has made substantial progress in engineering bacterial patterning systems. Many such systems demonstrate non-autonomous patterning, leveraging projected light masks, prepatterned templates, or other fabrication tools to spatially guide cellular behaviors, including fluorescence, pigment production, biofilm formation, or swimming motility (Tabor et al, 2009; Huang et al, 2019; Kim et al, 2022; Arlt et al, 2018). Complementary autonomous patterning systems instead program genetic circuits to generate spatial structures intrinsically through mechanisms such as quorum sensing, diffusible morphogen gradients, or swimming motility–based self-organization (Danino et al, 2010; Karig et al, 2018; Liu et al, 2011; Curatolo et al, 2020). Collectively, these studies have provided powerful strategies for engineering spatial organization in microbial systems across a range of scales and community compositions, yielding insights into mechanisms of multicellular coordination and pattern formation.

Another strategy for generating spatial organization lies in bacterial swarming, a distinct motility mechanism that differs from many prior patterning systems based on swimming, growth, or externally imposed spatial cues. Through coordinated surface expansion, swarming innately produces large-scale, stable, and information-rich patterns. These features complement DNA-based recorders, which store information in genomic sequences that require sequencing for readout (Sheth et al, 2017; Shipman et al, 2017), as well as phenotypic recorders that encode input signals into measurable cellular outputs such as gene-expression dynamics or spatial patterns (preprint: Duncker et al, 2025; Basu et al, 2005; Prindle et al, 2012). Swarm morphologies provide a particularly attractive phenotype because their spatial structure captures how cells respond to environmental cues across time and large populations, making them well-suited for intuitive visible outputs (Dietrich et al, 2008; Prindle et al, 2015; Lu et al, 2022; Doshi et al, 2023).

Building on this concept, we leverage *Escherichia coli*—a genetically tractable chassis whose swarming behavior remains comparatively underexplored as a programmable biosensor—to create new logic-based recording systems (Budrene and Berg, 1991, 1995; Bhattacharyya et al, 2023). Using modular genetic circuits and the extensive synthetic biology toolkit available in *E. coli*, we engineer swarming populations to record chemical and optical inputs in both binary and analog modes within spatiotemporal colony features. To decode colony phenotypes across space and time, we develop flexible and scalable computational pipelines. Together, these advances establish a modular and robust *E. coli*-based platform for engineering coordinated cellular behaviors and advancing biological information processing.

## Results

### Programming *E. coli* swarming

**Figure 1:**
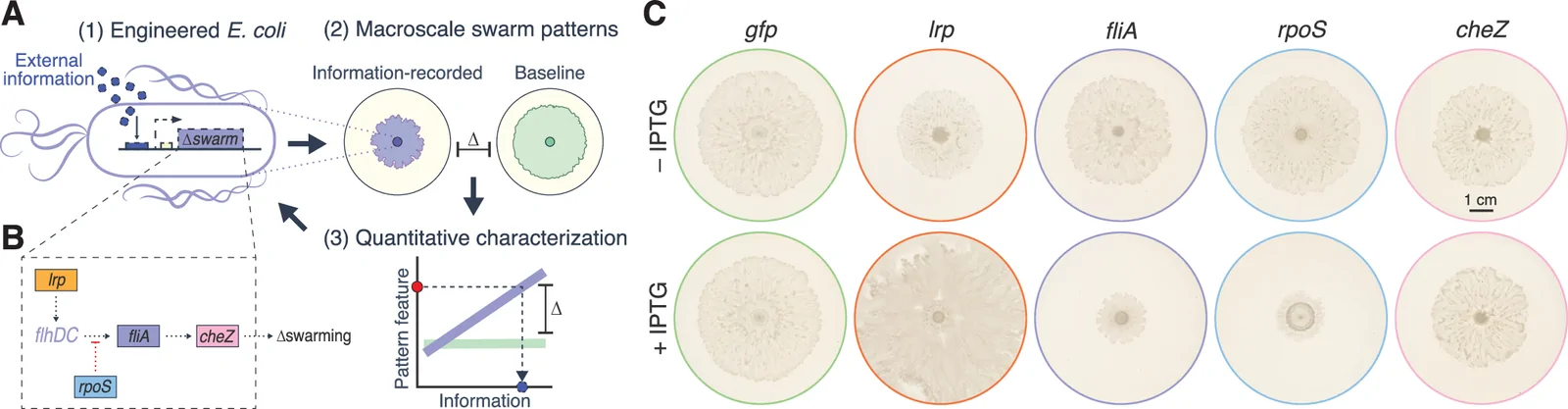
Engineering information-responsive swarming with *E. coli.* (**A**) Information recording and decoding in engineered bacteria occurs via three main steps. (1) Engineered hypermotile *E. coli* cells sense predefined inputs and modulate expression of a swarming-related gene. (2) Tuned swarming motility alters colony pattern formation in time and space. (3) Computational methods quantitatively interpret the patterns and decode the recorded information. (**B**) Candidate genes cloned onto pLac plasmids for top-down modulation of swarming. Each gene plays a distinct role within *E. coli*’s native motility pathway, approximated in a high-level network diagram centered on the Class I master flagellar operon *flhDC*. (**C**) Representative images of 24-h swarm colonies formed by engineered strains on swarming medium with and without IPTG. Overexpression of *lrp*, *fliA*, and *rpoS*—but not *cheZ* or the *gfp* control—visibly altered the baseline pattern. IPTG concentrations—selected to illustrate representative high-induction phenotypes for each construct—were 10 mM for pLac-*gfp*, -*fliA*, and -*rpoS*, and 2 mM for pLac-*lrp* and -*cheZ*. Source data are available online for this figure.

To enable spatial information recording in *E. coli* (Fig. 1A), we first optimized conditions for reproducible, macroscale swarm patterning. We defined an ideal baseline pattern as having consistent macroscopic morphology, readily distinguishable by eye and capturable using low-cost flatbed imaging. Screening the MG1655 strain and its genetic variants, we identified a hypermotile isolate (MG1655hm; CGSC #8237) that formed centimeter-scale, radially symmetric colonies on semi-solid agar with glucose (Appendix Fig. S1A; glucose supports flagella-driven swarming motility in *E. coli* by fueling cellular energy metabolism and the production of surface-wetting factors required for colony expansion) (Inoue et al, 2007). With this baseline pattern established, we tuned endogenous *E. coli* swarming pathways through inducible gene expression. We used a high-copy plasmid carrying an isopropyl β-d-1-thiogalactopyranoside (IPTG)-inducible pLac promoter driving green fluorescent protein (*gfp*) (Lutz, 1997). This circuit exhibited ~28-fold inducibility in liquid culture (Appendix Fig. S1B), providing sufficient dynamic range to perturb swarming-related genes without disrupting baseline pattern formation.

We next replaced *gfp* with a set of candidate genes implicated in *E. coli* swarming regulation and signaling. We selected *lrp*, *fliA*, *rpoS*, and *cheZ* as targets for circuit-based modulation of swarming based on their distinct roles across the motility pathway (Fig. 1B). At the top of the transcriptional hierarchy, *lrp* (encoding the leucine-responsive regulatory protein) activates *flhDC*, the Class I master regulator of flagellar gene expression (Sun et al, 2022; Lintner et al, 2008). The FlhDC complex activates transcription of Class II genes involved in flagellar biosynthesis and regulation. One of these genes, *fliA*, encodes the flagellum-specific sigma factor σ^28^ that activates transcription of Class III genes for flagellar assembly and chemotaxis (Sun et al, 2022). Separately, the global stress regulator *rpoS* (encoding the alternative sigma factor σ^S^) indirectly represses flagellar gene expression under stress conditions (Farewell et al, 1998; Ojima et al, 2012). Within the chemotaxis network, the phosphatase gene *cheZ* promotes the forward motion of runs by reducing tumble bias through rapid signal termination (Partridge et al, 2019). We cloned each gene from the MG1655 genome downstream of the pLac promoter, thereby creating four experimental circuits for inducible overexpression, with *gfp* serving as a control (Appendix Tables S1–S2).

To test whether basal or IPTG-induced overexpression of the selected genes modifies the MG1655hm swarm colony, we conducted 24-h swarming assays under fine-tuned conditions with and without IPTG (Fig. 1C; Appendix Fig. S1C). *cheZ* overexpression slightly decreased colony size in both uninduced and induced states but did not alter colony morphology. The reduced colony size observed in both states is consistent with mild basal leakiness from the pLac promoter. Prior work suggests that swarming cells naturally exhibit low tumble bias associated with elevated CheZ activity, and that increasing CheZ beyond this level can reduce colony expansion (Partridge et al, 2019; Virgile et al, 2018).

In contrast to the *gfp* control and *cheZ* construct, induction of the other motility regulators yielded distinct morphological changes in the swarm colonies. *lrp* overexpression produced feather-like branching structures and increased colony area, reflecting its role as an upstream activator of the *flhDC* flagellar regulon (Sun et al, 2022; Lintner et al, 2008). *fliA* overexpression yielded smaller, denser colonies with jagged borders. Although *fliA* promotes expression of late flagellar genes, excessive activation may disrupt the regulatory balance of the flagellar transcriptional hierarchy or impose energetic costs associated with flagellar biosynthesis and rotation, thereby limiting swarm expansion (Sun et al, 2022; Fitzgerald et al, 2014). *rpoS* overexpression also produced smaller colonies characterized by a dense inner ring and short branching protrusions, consistent with its known inhibitory effects on flagellar gene expression and motility (Farewell et al, 1998; Ojima et al, 2012). Perturbations of flagellar regulation via *fliA* and *rpoS* induction may destabilize coordinated outward swarm expansion, leading to irregular borders at the advancing colony front. Despite their distinct swarming responses to IPTG, all strains maintained mean liquid-culture growth rates above 60% of their baselines, indicating adequate fitness across the tested induction range (Appendix Fig. S1D). These results demonstrate that targeted overexpression of motility-related genes produces distinct swarming phenotypes.

### Characterizing binary and analog-recording strains

**Figure EV1:**
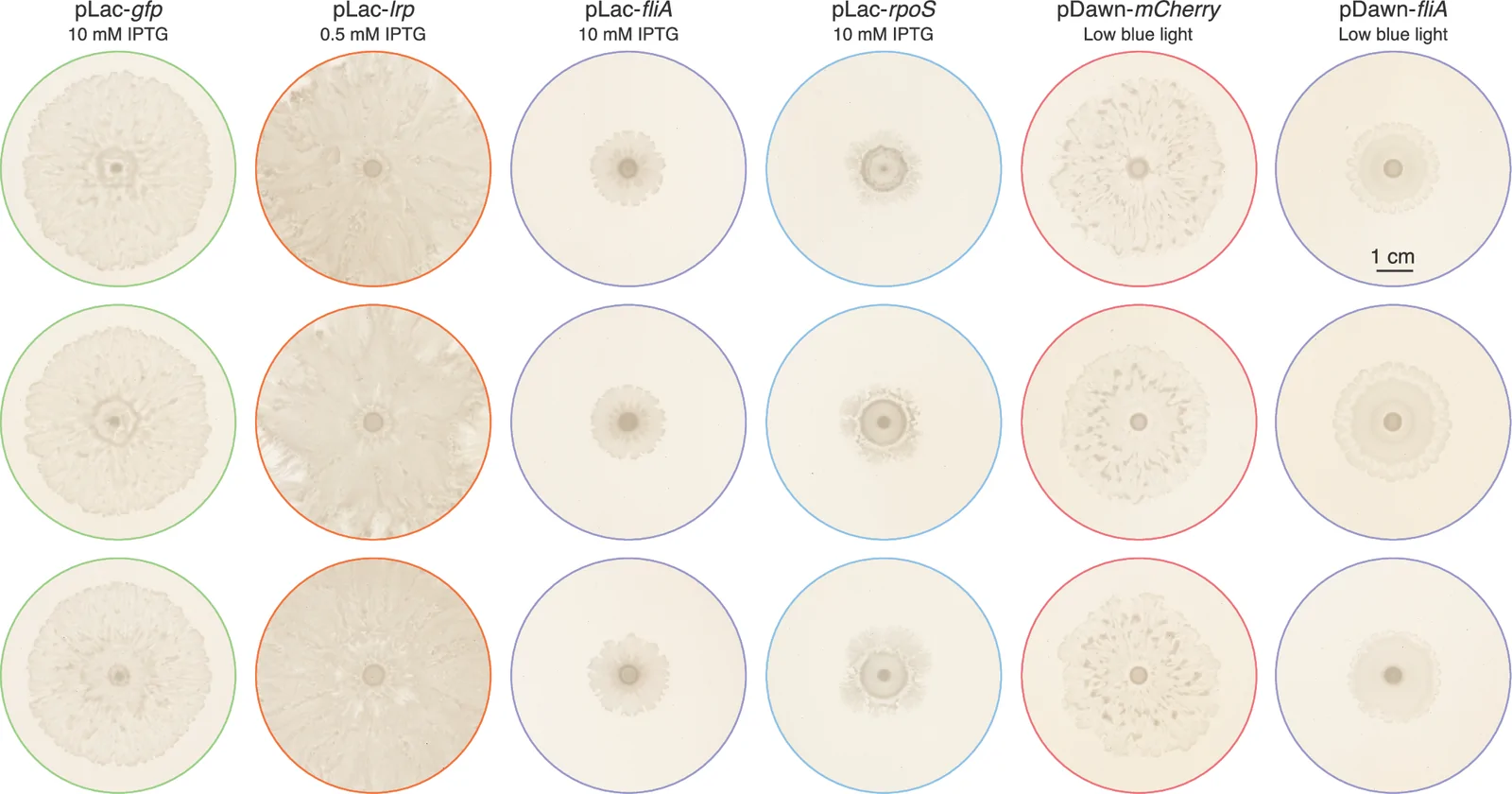
Engineered swarm colonies exhibit reproducible morphologies across biological replicates and experimental days. Representative replicate images of 24-h swarm colonies formed by engineered MG1655hm strains on swarming medium with select induction conditions (IPTG or blue light, as indicated). For each strain, a single induction condition was chosen from screening assays to illustrate characteristic morphologies. Across rows, three independent biological replicates are shown (*n* = 3), demonstrating reproducibility across experiments.

**Figure EV2:**
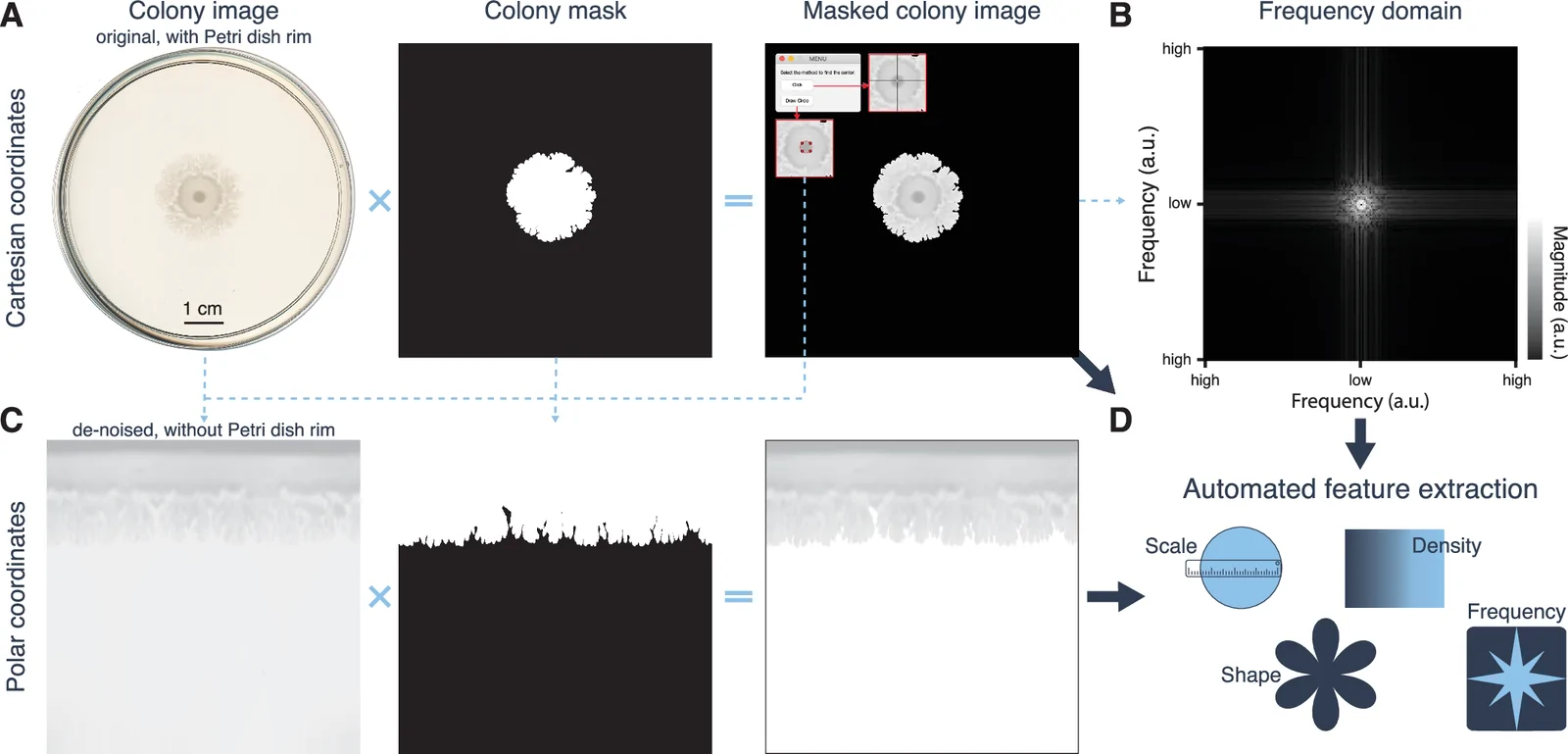
Semi-automated computational pipeline for swarm pattern quantification. (**A**) Swarm colonies in 100-mm Petri dishes were scanned at 400 dpi using a commercially available flatbed document scanner. Colony images were segmented with image-processing functions and minor manual refinement in Adobe Photoshop as needed. Masked colonies were transformed from the spatial to (**B**) frequency domain (via Fourier transform), and from Cartesian to (**C**) polar coordinates, with user input to locate the inoculum center. (**D**) From these three colony representations (Cartesian, Fourier, and polar), features of scale, density, shape, and frequency were extracted for downstream analysis. (Example shown: MG1655hm pLac-*rpoS* at 2.5 mM IPTG, 24 h).

To evaluate the strains’ information-recording behaviors, we conducted 24-h swarming assays across a broader range of IPTG concentrations. For each strain, we identified five concentrations (including 0 mM) that were practical to replicate experimentally while adequately capturing the strain’s characteristic swarming responses (Fig. EV1). To quantitatively characterize the observed pattern changes, we developed a semi-automated computational pipeline, scaling colony segmentation, feature extraction, and feature analysis across datasets (Fig. EV2). By analyzing significant changes in features related to colony size, shape, density, and texture, we categorized select engineered strains as binary- or analog-like information recorders.

**Figure 2:**
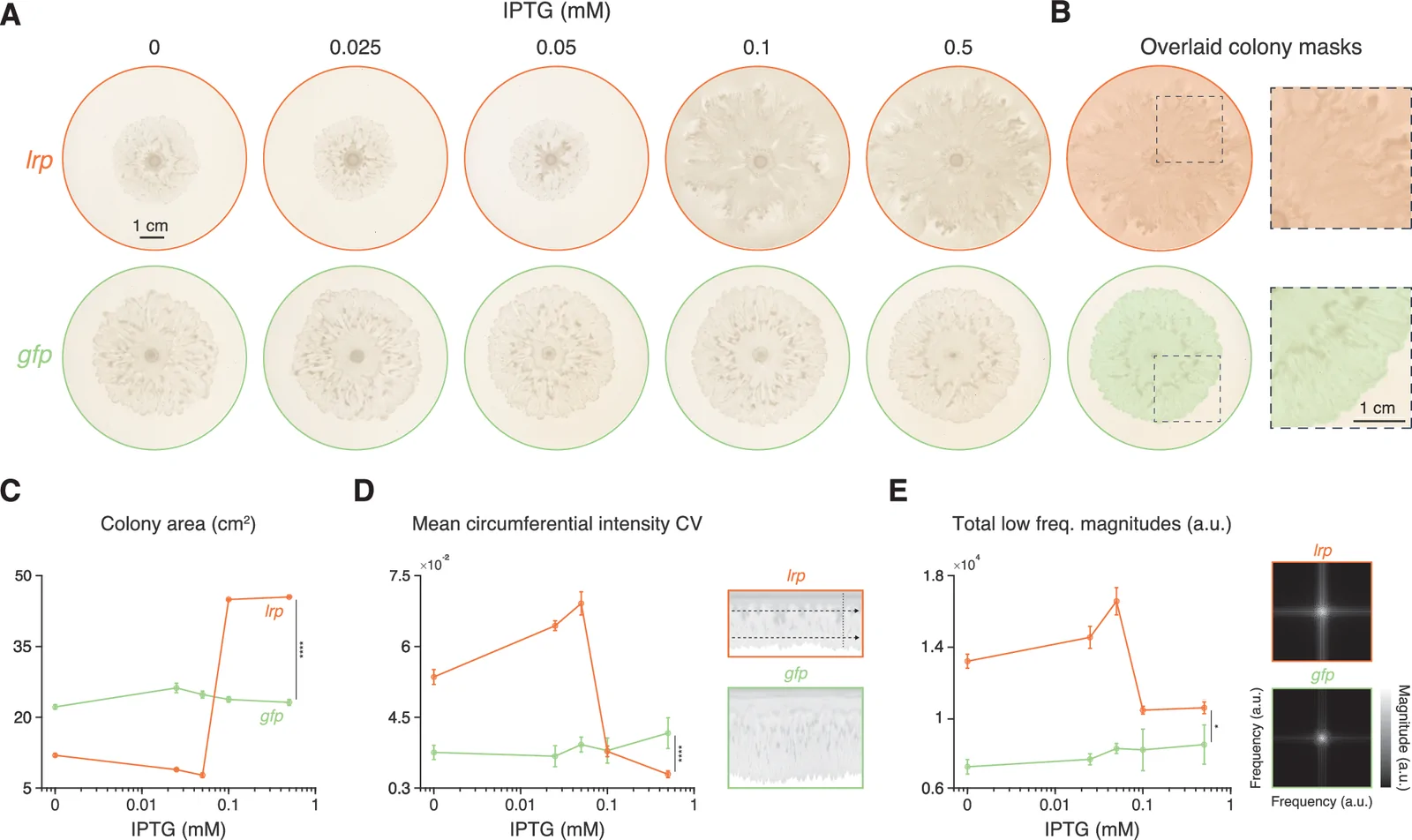
Modulation of *lrp* gene results in binary-like information recording. (**A**) Representative images of 24-h swarm colonies formed by MG1655hm pLac-*lrp* and pLac-*gfp* (control) on swarming medium supplemented with an empirically selected set of IPTG concentrations. (**B**), Representative false-color binary colony segmentation masks overlaid on the 0.5 mM IPTG images. All colony images and corresponding masks were used for analysis (see Fig. EV2 and Methods, “Colony segmentation” and “Colony feature extraction”). (**C**–**E**) Quantified features generally show stepwise changes at 0.1 mM IPTG for pLac-*lrp*, while remaining relatively constant for pLac-*gfp* across IPTG concentrations. Points represent the mean of *n* = 9–18 independent biological replicates (swarm colonies) collected across ≥3 independent experiments per condition performed on different days. Error bars represent the standard error of the mean (SEM). Asterisks denote significant differences between the overall IPTG–response curves, determined using linear mixed-effects models with ANOVA (Strain × IPTG interaction; *P* ≤ 0.01; see Methods, “Statistical analysis of colony features”). Exact *P* values are reported in Appendix Table S3. **C** Colony area calculated from Cartesian-coordinate masks. **D** Mean circumferential intensity coefficient of variation (CV), reflecting asymmetry in internal density or global shape, computed from polar-coordinate images on a row-wise basis. Representative masked polar-coordinate colony images at 0.05 mM IPTG are shown. Dashed arrows indicate the left-to-right direction along each circumferential row over which pixel intensities are sampled, and the vertical dotted line indicates that CV values are computed for all rows and subsequently averaged. **E** Total low-frequency magnitudes from Fourier transforms of cropped Cartesian images. Representative spectra at 0.025 mM IPTG are shown. Spectra are radially symmetric, with low frequencies at the center and high frequencies at the edges. Source data are available online for this figure.

The pLac-*lrp* strain stood out for its threshold-like response across IPTG concentrations (Fig. 2A). In the presence of no or minimal IPTG, pLac-*lrp* produced the baseline colony, albeit slightly smaller. Induced with 0.1 mM IPTG or higher, the strain’s swarming expanse increased significantly, covering the entire Petri dish (Fig. 2B,C). A coefficient of variation (CV) metric captured increased density variation at low IPTG, as small lobate, branch-like features emerged from the inoculum (Fig. 2D). CV dropped steeply at the threshold concentration, when these features expanded and homogenized across the dish. A similar non-monotonic trend appeared in total low-frequency magnitudes: a slight initial increase from alternating light and dark regions around the inoculum, followed by a sharp decrease as darker, high-density features dominated the colony texture (Fig. 2E). The pLac-*gfp* control strain exhibited relatively constant feature values across the IPTG threshold, whereas pLac-*lrp*’s step-like changes positioned it as a threshold-based, binary information recorder.

**Figure 3:**
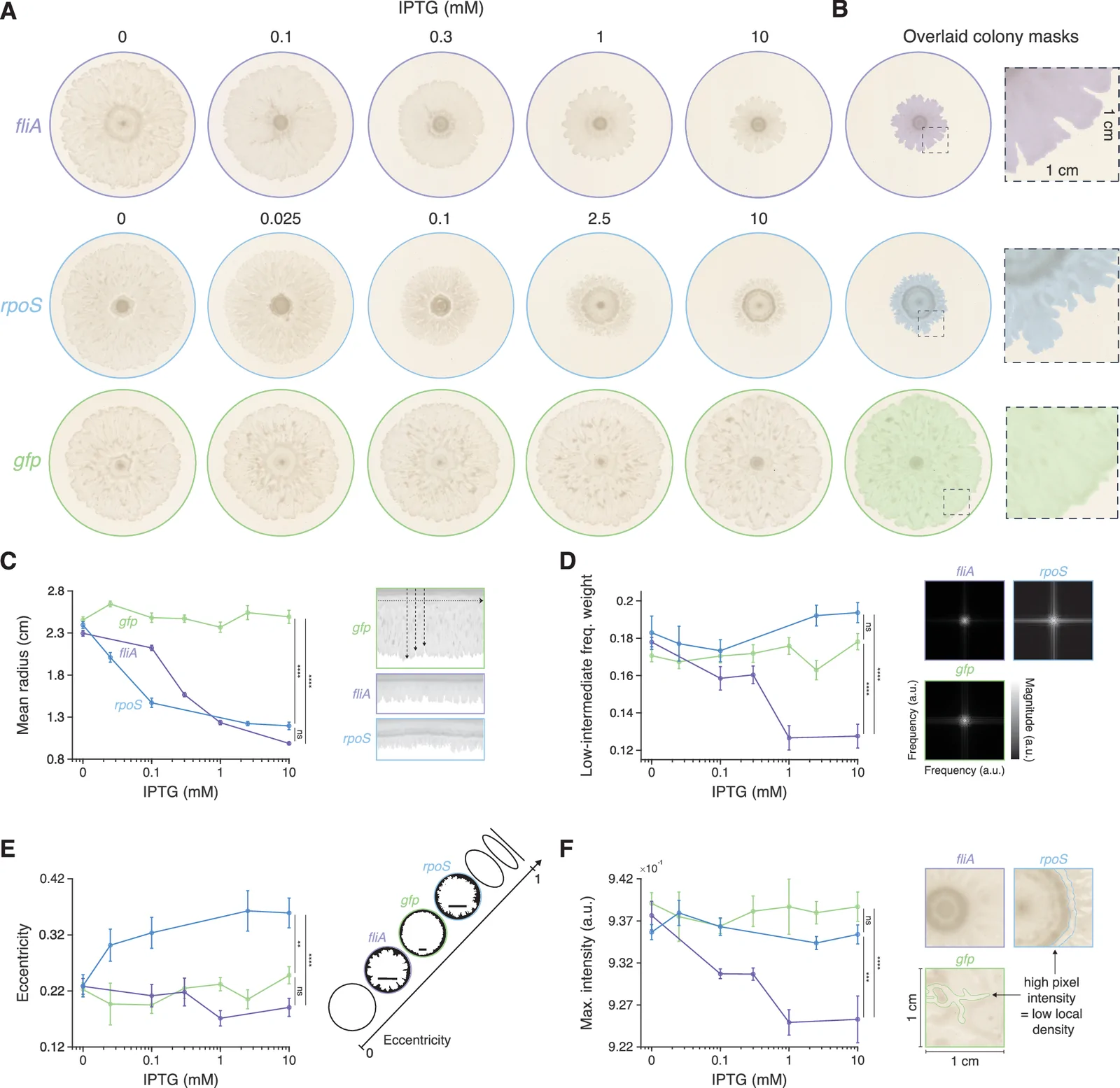
*fliA* and *rpoS* genes result in analog-like information recording. (**A**) Representative images of 24-h swarm colonies formed by MG1655hm pLac-*fliA*, pLac-*rpoS*, and pLac-*gfp* (control) on swarming medium supplemented with empirically selected IPTG concentrations. The pLac-*gfp* row shows control colonies grown in parallel with the pLac-*rpoS* experiments under the same IPTG concentrations; pLac-*gfp* controls grown in parallel with the pLac-*fliA* experiments are shown in Appendix Fig. S2. (**B**) Representative false-color binary colony segmentation masks overlaid on the 10 mM IPTG images. All colony images and corresponding masks were used for analysis (see Fig. EV2 and Methods, “Colony segmentation” and “Colony feature extraction”). (**C**–**F**) Quantified features generally show gradual, analog-like changes with increasing IPTG concentration for pLac-*fliA* and/or pLac-*rpoS*, while remaining relatively constant for pLac-*gfp*. Representative images at 10 mM IPTG, shown aside each plot, illustrate how the feature was extracted or how it manifests in colony morphology. Points represent the mean of *n* = 9–35 independent biological replicates (swarm colonies) collected across ≥3 independent experiments per condition performed on different days. Error bars represent SEM. Asterisks denote significant differences between the overall IPTG–response curves, determined using linear mixed-effects models with post hoc comparisons (Strain × IPTG interaction; *P* ≤ 0.01; see Methods, “Statistical analysis of colony features”). Exact *P* values are reported in Appendix Table S3. **C** Mean colony radius calculated from polar-coordinate masks by averaging the distance from the inoculation point to the colony edge across angular columns. In the representative polar-coordinate image, vertical dashed arrows indicate the radial direction along which distances are sampled, and the horizontal dotted line indicates that distances are computed for all columns and subsequently averaged. **D** Weight of low–intermediate frequencies from Fourier transforms of cropped Cartesian images. Spectra are radially symmetric, with low frequencies at the center and high frequencies at the edges. **E** Colony eccentricity, measuring deviation from circularity, calculated directly from Cartesian masks (scale bars = 1 cm). **F** Maximum pixel intensity within masked colony regions, where higher intensity corresponds to lower local density. Source data are available online for this figure.

Unlike the quasi-binary recording observed in pLac-*lrp* colonies, the pLac-*fliA* and pLac-*rpoS* strains exhibited more gradual, analog-like responses across wide IPTG ranges (Fig. 3A,B). Via strain-specific screening, we selected distinct IPTG concentrations to capture the IPTG window over which pLac-*fliA* and pLac-*rpoS* exhibited measurable morphological changes. As IPTG increased, both strains progressively decreased their colony radii (Fig. 3C), while other feature changes appeared more strain specific. pLac-*fliA* displayed a gradual, step-like decline in low–intermediate frequency weight, likely reflecting a decrease in broad, low-detail features and/or an emergence of finer-scale texture not readily visible by eye (Fig. 3D). In contrast, pLac-*rpoS* showed a slight increase in this frequency feature at maximal induction, potentially due to a dark inner ring bordered by a lighter region. Colony shape metrics also diverged, with eccentricity values indicating that pLac-*rpoS* colonies became increasingly asymmetric with IPTG, whereas pLac-*fliA* colonies—despite jagged borders—modestly approached greater global symmetry (Fig. 3E). Intensity features further distinguished the strains: pLac-*fliA* colonies recorded IPTG through a gradual stepwise decline in maximum pixel intensity, consistent with increased internal density, while pLac-*rpoS*, maintaining light wispy branches, showed only minimal changes (Fig. 3F). As in the *lrp* experiments, induced *gfp* expression did not significantly alter colony features across IPTG concentrations (Appendix Fig. S2). Overall, by progressively modifying such features, the pLac-*fliA* and pLac-*rpoS* strains rounded out the information-recording toolbox with analog-like recording capacities that complemented the binary-like behavior of pLac-*lrp*. In these IPTG-screening assays, *cheZ* overexpression consistently preserved the baseline swarm colony at all induction levels, suggesting that only select motility-related genes—such as *lrp*, *fliA*, and *rpoS*—yield distinctive, tunable morphological responses under inducible control (Appendix Fig. S3).

### Decoding information from engineered swarm patterns

**Figure 4:**
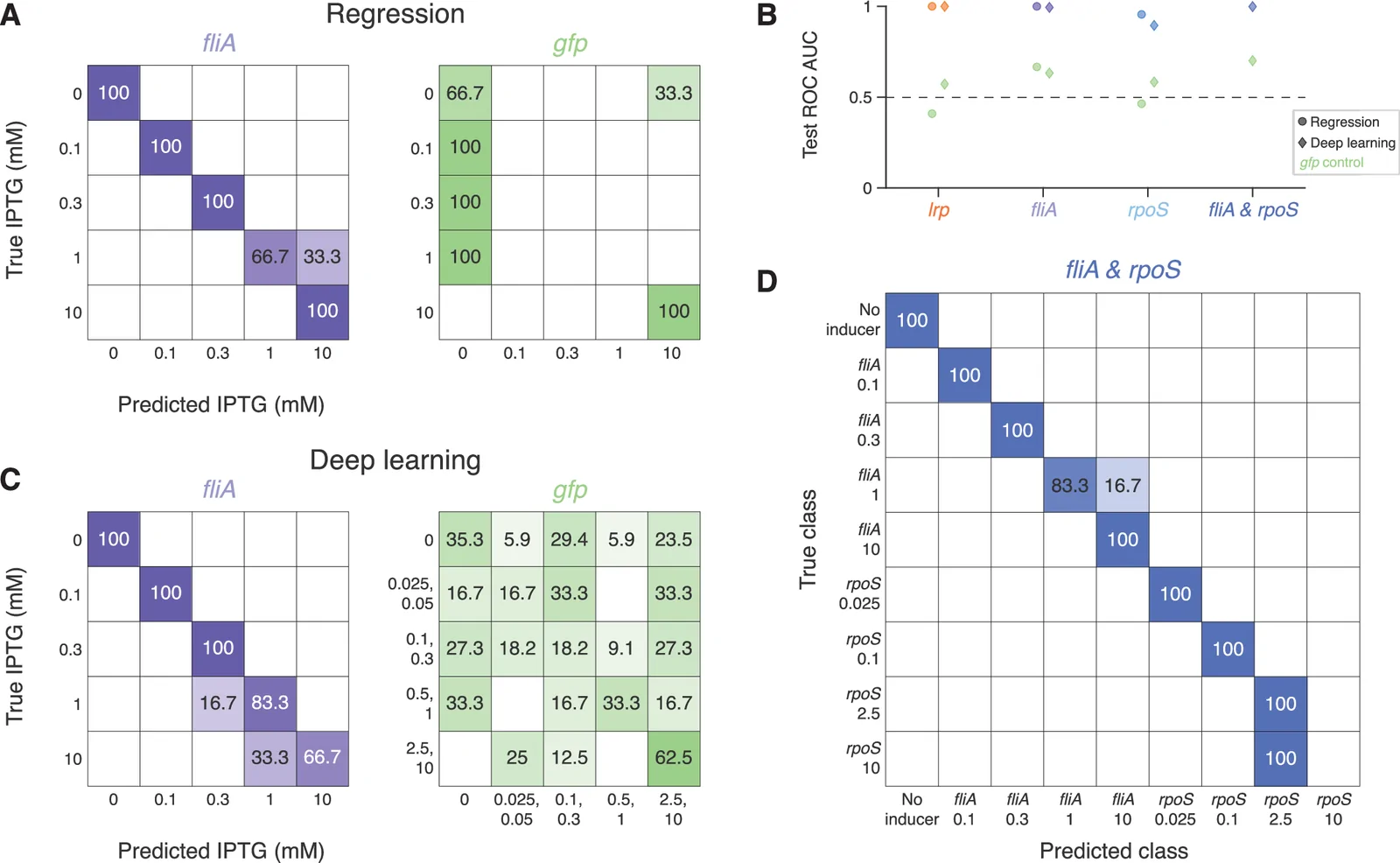
Computationally decodable swarm patterns. (**A**) Test confusion matrices of representative regression models decoding IPTG concentration from 24-h pLac-*fliA* and pLac-*gfp* colony features. (**B**) Test ROC–AUC values for regression and deep-learning models across tasks: binary IPTG decoding (pLac-*lrp*), 5-class IPTG decoding (pLac-*fliA*, pLac-*rpoS*), and 9-class joint strain–IPTG decoding (pooled pLac-*fliA* and pLac-*rpoS*), with corresponding pLac-*gfp* control models. (**C**) Test confusion matrices of representative deep-learning models decoding IPTG concentration from 24-h pLac-*fliA* and pLac-*gfp* colony images. (**D**) Test confusion matrix of a dual-strain deep-learning model jointly decoding IPTG concentration and strain identity from pooled 24-h pLac-*fliA* and pLac-*rpoS* colony images. In all confusion matrices (**A**, **C**, **D**), values indicate the percentage of test predictions within each true class. Additional regression results are shown in Fig. EV3; additional single-strain deep-learning results in Fig. EV4; and additional deep-learning results for the dual-strain decoding task in Appendix Fig. S4. Source data are available online for this figure.

**Figure EV3:**
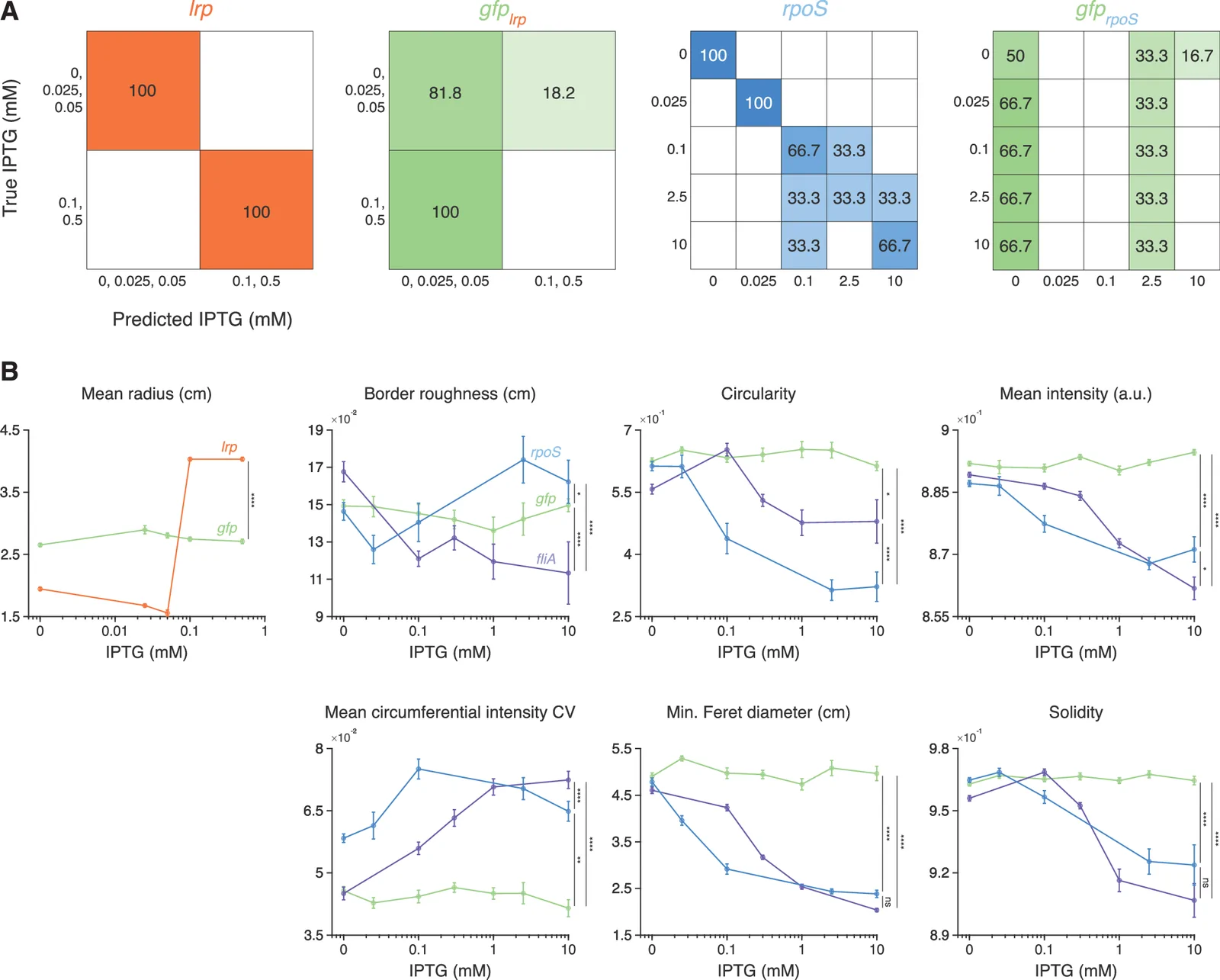
Regression models decode IPTG from swarm pattern features. (**A**) Test confusion matrices of regression models decoding IPTG concentration from 24-h colony features of pLac-*lrp*, pLac-*rpoS*, and their corresponding pLac-*gfp* control strains. Confusion matrices share the same axes; inner values indicate the percentage of test predictions within each true IPTG class. Confusion matrices for the pLac-*fliA* model and its corresponding pLac-*gfp* control model are shown in Fig. 4A. (**B**) Select IPTG-dependent trends of morphological and intensity-based features identified through strain-specific model fitting. The pLac-*lrp* model used mean radius; the pLac-*fliA* model used border roughness, circularity, and mean intensity; and the pLac-*rpoS* model used mean circumferential intensity CV, minimum Feret diameter, and solidity. Points represent the mean of independent biological replicates (swarm colonies): *n* = 9–18 for the pLac-*lrp*/*gfp* strain set, and *n* = 9–35 for the pLac-*fliA*/*rpoS*/*gfp* strain sets, collected across ≥3 independent experiments per condition performed on different days. Error bars represent SEM. Asterisks denote significant differences between the overall IPTG–response curves, determined using linear mixed-effects models with ANOVA for the pLac-*lrp*/*gfp* strain set and with post hoc comparisons for the pLac-*fliA*/*rpoS*/*gfp* strain sets (both based on the Strain × IPTG interaction; *P* ≤ 0.01; see Methods, “Statistical analysis of colony features”). Exact *P* values are reported in Appendix Table S3.

With the image and feature datasets, we aimed to extend our computational pipeline beyond pattern quantification to pattern decoding—that is, back-calculating the information recorded within engineered swarm colonies. To assess how reliably certain colony features support decoding binary or multiclass IPTG levels, we fit regression models independently for each strain and evaluated their performances on held-out test sets. The optimal model for each strain leveraged a unique combination of statistically significant features to achieve the highest test metrics (Figs. 4A,B and EV3; Appendix Tables S4 and S5). The pLac-*lrp* model performed binary IPTG decoding perfectly, achieving 100% accuracy and an area under the receiver operating characteristic curve (ROC–AUC) of 1. The analog pLac-*fliA* and pLac-*rpoS* models achieved five-class decoding accuracies of 94 and 78%, with AUCs of 1 and 0.96, respectively. The slightly higher performance of the pLac-*lrp* model reflects the highly separable, threshold-like nature of the strain’s phenotypic response and the accordingly simpler binary decoding task. In contrast, the pLac-*fliA* and pLac-*rpoS* strains record IPTG across five classes with more gradually varying morphologies that are harder to separate perfectly. The pLac-*gfp* control models exhibited low decoding performance, with test metrics near random guessing (AUC ≈ 0.5), suggesting weak or inconsistent IPTG recording in pLac-*gfp* colony features as expected. These results demonstrate that extracted morphological features not only quantify observable differences in information-recorded patterns, but also serve as reliable inputs for computational models that decode the information.

**Figure EV4:**
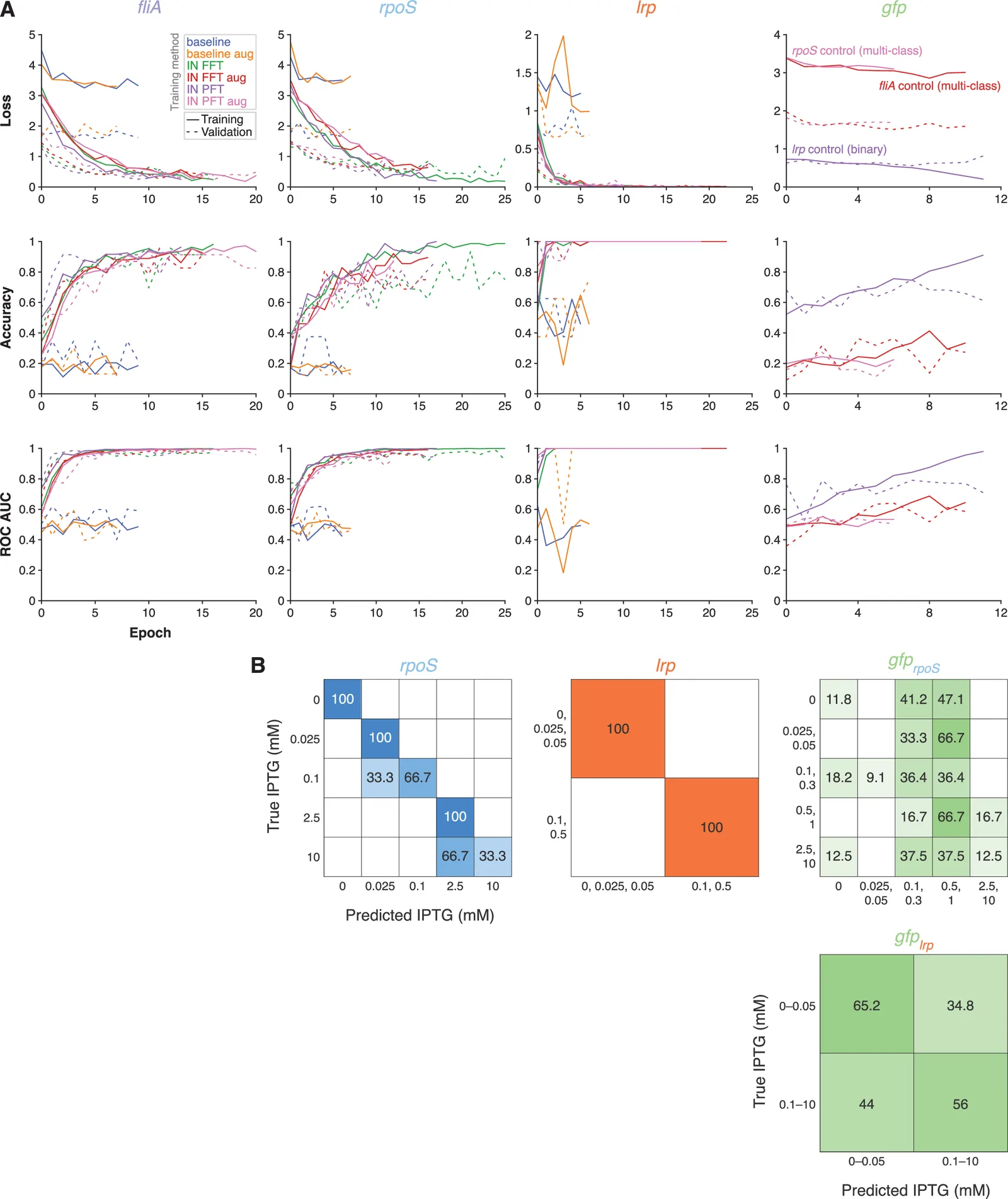
Deep-learning models decode IPTG from swarm colony images. (**A**) Training and validation learning curves (loss, accuracy, ROC–AUC) for SwinTransformer models trained on pLac-*fliA*, pLac-*rpoS*, pLac-*lrp*, and pLac-*gfp* image datasets. Model configurations included ImageNet pretraining (IN), full or partial fine-tuning (FFT, PFT), and random data augmentation (aug). Baseline models lacked ImageNet pretraining and fine-tuning. (**B**) Test confusion matrices of the optimal models for pLac-*rpoS* and pLac-*lrp*, defined as those achieving the lowest validation loss, and their corresponding pLac-*gfp* control models. The pLac-*gfp* models were trained and evaluated for multiclass IPTG classification (top; corresponding to the pLac-*rpoS* model configurations) and binary IPTG classification (bottom; corresponding to the pLac-*lrp* model configurations). Confusion matrices for the pLac-*fliA* model and its corresponding pLac-*gfp* control model are shown in Fig. 4C. Axes are shared across confusion matrices.

Given that the human eye can readily distinguish the engineered swarm colonies, we reasoned that computer vision models could similarly learn to decode the information directly from colony images. We thus trained deep-learning models to classify IPTG concentration from 24-h endpoint swarm colony images, bypassing the handcrafted feature extraction required for the regression models. Using the hierarchical vision transformer architecture SwinTransformer (Liu et al, 2021), we trained separate models on each strain’s image dataset, addressing limited dataset sizes through pretraining, fine-tuning, and data augmentation (Fig. EV4A and Appendix Table S6). For each strain, the best-performing model utilized a distinct combination of optimization strategies and demonstrated strong generalization on unseen test sets (Figs. 4B,C and EV4B; Appendix Table S7). Mirroring its regression counterpart, the optimal pLac-*lrp* model achieved 100% accuracy and an AUC of 1 in binary IPTG classification. The optimal pLac-*fliA* and pLac-*rpoS* models likewise performed comparably to their regression counterparts, achieving accuracies of 92 and 82%, Top-2 accuracies of 96 and 88%, and AUCs of 0.99 and 0.89, respectively. Again, the control models performed poorly in predicting IPTG concentration from pLac-*gfp* images, confirming that high decoding performance was specific to strains that reliably recorded IPTG in responsive swarm patterns. Deep learning therefore provides an alternative route to information decoding that can scale flexibly without handcrafted feature design.

To further evaluate scalability and multiplexing capacity, we trained a unified deep-learning model to decode both strain identity and IPTG concentration from pooled pLac-*fliA* and pLac-*rpoS* images in a nine-class classification task. Despite the increased complexity of jointly distinguishing gene- and input-dependent morphological features, the model achieved 93% accuracy, 98% Top-2 accuracy, and a near-perfect macro-averaged AUC of 0.9986 on unseen test data (Fig. 4B,D; Appendix Fig. S4; Table S7). This performance exceeded that of the individual strain-specific deep-learning models, demonstrating that multiplexed decoding can be achieved without sacrificing accuracy. In contrast, a matched nine-class pLac-*gfp* control model achieved only 18% accuracy, 41% Top-2 accuracy, and a macro-averaged AUC of 0.7009. Although the control AUC reflects weak above-chance ranking structure, the widespread misclassification across IPTG classes suggests limited class-specific separability in the absence of engineered morphological responses. The strong performance of the unified model, coupled with the substantially lower performance of the control model, indicates that decoding success arises from biologically structured, dose-dependent pattern formation and supports the extension of this framework to multiplexed sensing applications.

### Dynamic features support early decoding and transient input recording

**Figure 5:**
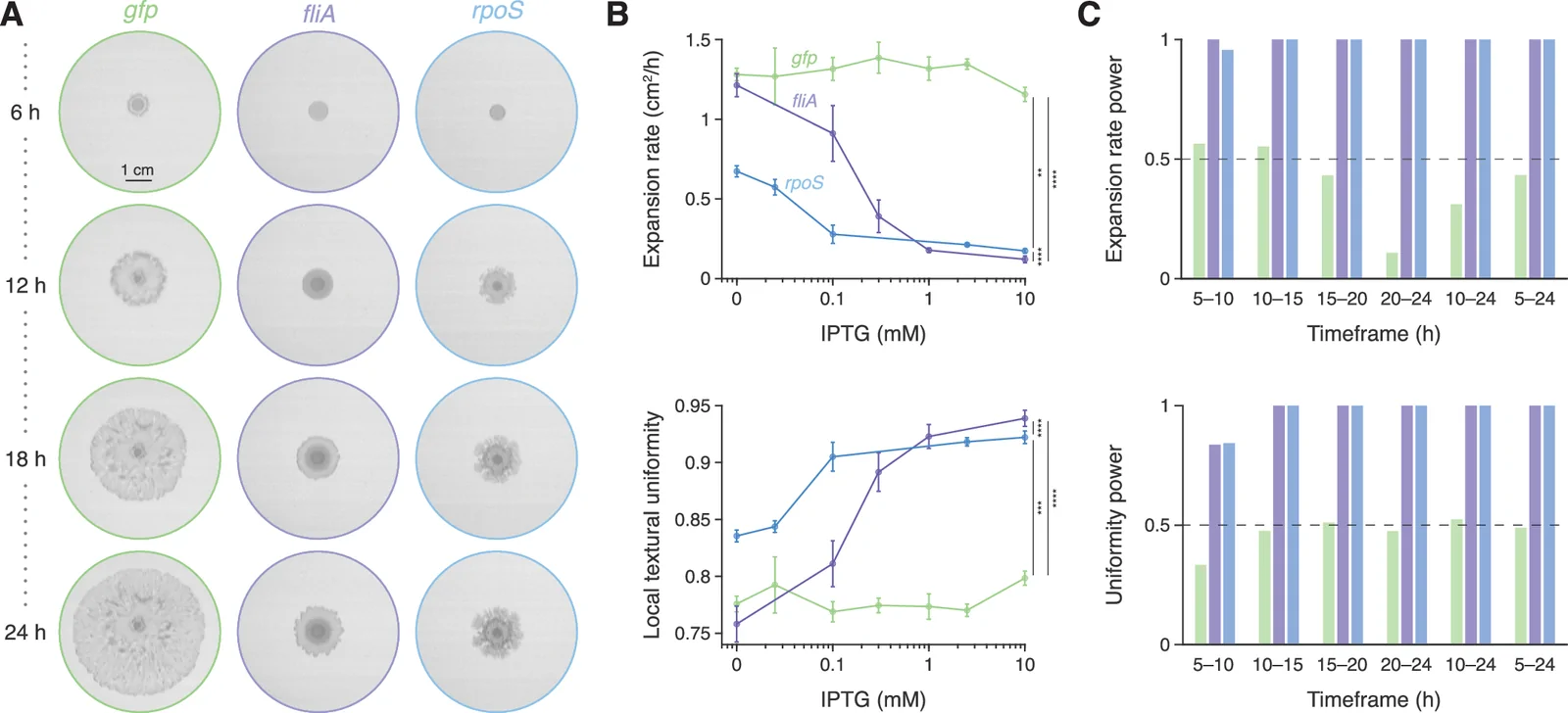
Dynamic swarming features support early distinction of recorded IPTG. (**A**) Six-hour snapshot images from representative 24-h time-lapse swarming assays. Shown are control (pLac-*gfp*) and analog (pLac-*fliA* and pLac-*rpoS*) strains on swarming medium supplemented with 10 mM IPTG. (**B**) IPTG-dependent responses in colony expansion rate (top) and local textural uniformity (bottom), averaged between hours 5 and 24. Points represent the mean of *n* = 3–12 independent biological replicates collected across independent experiments performed on different days (typically ≥3 per condition); error bars represent SEM. Asterisks denote significant differences between the complete IPTG–response curves, determined using linear mixed-effects models with post hoc comparisons (Strain × IPTG interaction; *P* ≤ 0.01; see Methods, “Time-lapse feature analysis”). Exact *P* values are reported in Appendix Table S3. (**C**), Statistical power to distinguish 0 vs. 10 mM IPTG using each strain’s colony expansion rate (top) and local textural uniformity (bottom) across different time windows (significance level α = 0.05). Source data are available online for this figure.

Using the dynamic nature of swarming, we investigated whether we could use temporal features in conjunction with spatial features to enhance our platform’s recording and decoding capabilities. We focused on characterizing the dynamics of pLac-*fliA* and pLac-*rpoS*, the two strains that collectively recorded seven IPTG concentrations in distinct colony morphologies. To maintain our previously optimized swarming conditions while continuing to use accessible technology, we repurposed a commercial flatbed scanner to capture high-resolution scans of Petri dishes every 10 min during 24-h swarming assays at 37 °C. We acquired 75 time-lapse series across representative IPTG concentrations and strains, with each series comprising approximately 145 image frames (Fig. 5A; Movies EV1 and EV2). The resulting large-scale dataset motivated us to develop a semi-automated pipeline that combined limited user input with automated temporal feature extraction, enabling high-throughput analysis across hundreds of frames and complementing our endpoint analysis pipeline.

As an example of a dynamic, time-dependent feature, we quantified colony expansion rate by calculating the frame-by-frame change in colony area over time (Fig. 5B, top). Despite IPTG-induced *gfp* expression, the control strain expanded at a relatively constant rate of ~1.3 cm^2 ^h^−1^ over 5–24 h. The pLac-*fliA* and pLac-*rpoS* strains, however, recorded IPTG through decreasing expansion rates, consistent with the non-linear reductions in final colony size we previously observed. From uninduced baselines of ~1.2 and ~0.7 cm^2 ^h^−1^ respectively, pLac-*fliA* and pLac-*rpoS* expansion rates decreased with IPTG along distinct trajectories, converging to ~0.2 cm^2 ^h^−1^ at maximal induction. To assess how early expansion rate could help us distinguish recorded information, we performed a power analysis comparing measurements at 0 vs. 10 mM IPTG across different timeframes (Fig. 5C, top). Both pLac-*fliA* and pLac-*rpoS* expansion-rate metrics achieved 100% statistical power across all timeframes, except for pLac-*rpoS* at 5–10 h, when power remained high at ~96%. In contrast, pLac-*gfp* expansion rate showed poor distinguishing power across all timeframes, ranging from ~11 to ~56%, further confirming the absence of IPTG recording in the control strain’s swarming dynamics.

Time-lapse imaging enables extraction of spatial features that, while not inherently time-dependent, may fluctuate, stabilize, or gain robustness when evaluated across timeframes. As one example, we assessed local colony texture by computing pixel uniformity within small gray-level co-occurrence matrices, where uniformity (also known as energy) is 1 for a constant image (Fig. 5B, bottom). With increasing IPTG amount, the radially striated pLac-*gfp* colonies maintained fine-scale local density variations, yielding a mean uniformity of ~0.78 over 5–24 h. Such variations became more locally homogenous in pLac-*fliA* and pLac-*rpoS* colonies, both distinctly trending toward values of ~0.93 at maximal induction. As with expansion rate, pLac-*fliA* and pLac-*rpoS* uniformity metrics enabled detection of 0 vs. 10 mM IPTG with ~100% power across all evaluated timeframes except at 5–10 h, when power for both strains dropped slightly to ~84% (Fig. 5C, bottom). The pLac-*gfp* uniformity metric achieved at best ~52% power, reflecting the absence of IPTG-recorded spatial features early in control colony development. Overall, these results demonstrate that by expanding the feature set beyond static endpoint measurements, time-lapse analysis strengthens quantitative understanding of swarm-based information recording and supports the possibility of early, accurate, and scalable decoding.

**Figure EV5:**
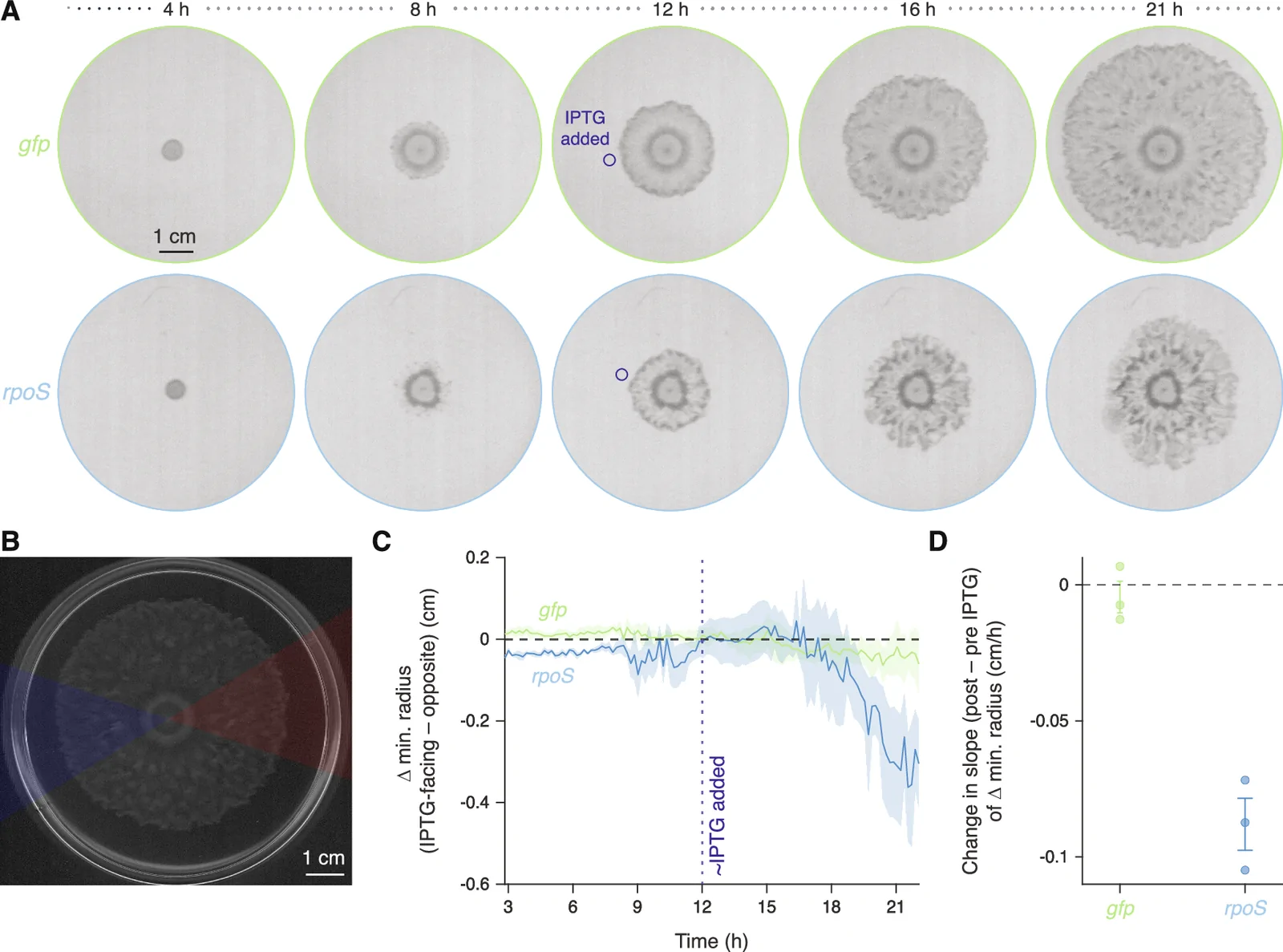
Localized IPTG perturbation induces asymmetric swarm expansion. (**A**) Four-hour snapshot images from representative time-lapse swarming assays. Shown are the control strain (pLac-*gfp*) and an analog strain (pLac-*rpoS*) on swarming medium. At ~12 h, 2 µL of 1 M IPTG was spotted just outside the expanding colony front. (**B**) Representative uncropped endpoint image of a pLac-*gfp* swarm colony (~22 h) with overlaid analysis masks. The blue wedge denotes the IPTG-facing region, and the red wedge denotes the opposite, untreated control region. (**C**) Time course of the difference in minimum colony radius between the IPTG-facing and opposite regions, with the timing of localized IPTG addition indicated. (**D**) Change in the slope of the minimum-radius difference post-IPTG relative to pre-IPTG. Two-sample, two-tailed Welch’s *t*-test: *P* = 3.5 × 10^−3^; Wilcoxon rank-sum test: *P* = 0.1. In plots (**C**, **D**), lines represent the mean of *n* = 3 biological replicates; shaded regions and error bars represent SEM; points indicate individual biological replicates.

Having established that engineered swarm colonies record constant IPTG in both spatial and temporal features, we next asked whether they could respond to an input that changes during colony growth. To introduce a transient, spatially localized perturbation, we pipetted a single drop of IPTG adjacent to the expanding swarm front ~12 h after the start of a time-lapse swarming assay (Fig. EV5A). To quantify directional responses in colony development, we measured the difference in minimum radius between the IPTG-facing and diametrically opposite regions over time (Fig. EV5B,C). Prior to IPTG addition, both pLac-*gfp* and pLac-*rpoS* exhibited near-zero radius differences, indicating symmetric radial expansion under baseline conditions. Following localized IPTG exposure, pLac-*gfp* maintained minimal directional deviation, whereas pLac-*rpoS* exhibited a reduction in minimum radius within the IPTG-facing region, decreasing by ~0.4 cm between ~15 and 21 h. Quantification of slope changes in this radius difference before and after IPTG addition confirmed this asymmetric response: pLac-*rpoS* displayed a negative shift in radial expansion slope, whereas pLac-*gfp* showed no substantial change (Fig. EV5D). Together, our findings show that engineered swarm colonies can record temporally and spatially varying inputs, extending beyond static induction conditions and supporting the applicability of this platform to dynamically varying environmental signals.

### Adapting swarming strains to record light

To test the modularity and utility of the platform beyond chemical induction, we adapted it to record an orthogonal input—blue light. We selected the optogenetic pDawn (Ohlendorf et al, 2012) system for its strong inducibility in *E. coli* and its demonstrated applications in diverse areas including therapeutics and materials fabrication (Jin and Riedel-Kruse, 2018; Magaraci et al, 2014; Yang et al, 2020; Wang et al, 2021). Compared with chemical inducers, light offers higher spatial and temporal resolution and enables transient, reversible control. Previous studies have used optogenetics to control cellular processes such as swimming motility, signaling, and differentiation—which all influence bacterial spatial organization—and inspired us to explore optogenetic control of swarming (Walter et al, 2007; Frangipane et al, 2018; Zhang et al, 2020; Repina et al, 2017; Mushnikov et al, 2019).

**Figure 6:**
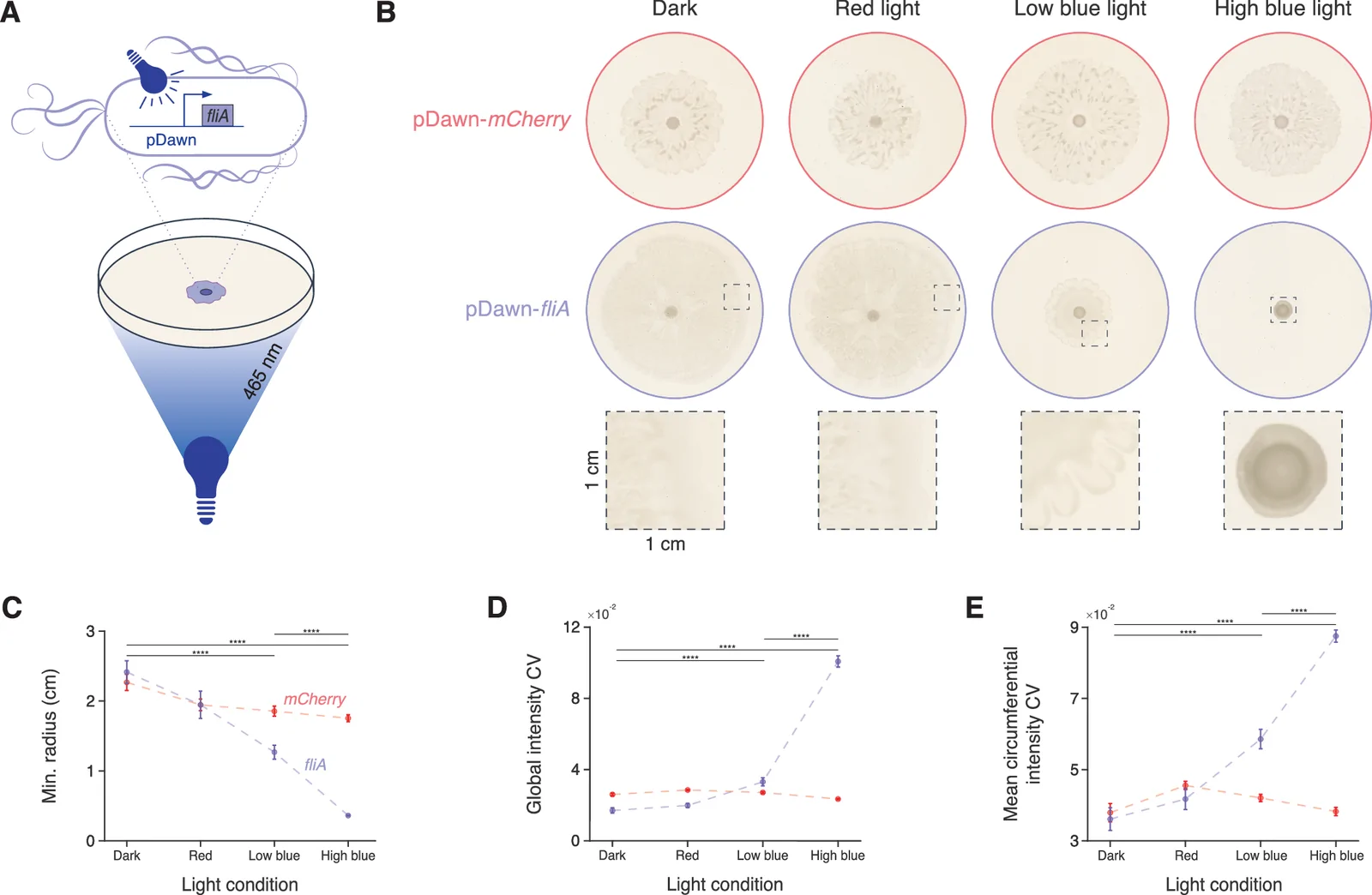
Blue-light–responsive swarm colonies. (**A**) Schematic of the pDawn-*fliA* system, in which blue light (465 nm) activates *fliA* overexpression in the MG1655hm background to modify swarming. (**B**) Representative images of 24-h swarm colonies formed by MG1655hm pDawn-*mCherry* (control) and pDawn-*fliA* on swarming medium under non-inducing (dark and red light) and inducing (low and high blue light) conditions. Low and high blue-light irradiances were 22.78 ± 1.28 and 74.75 ± 2.24 mW cm⁻², respectively; the dark condition measured 0.00 ± 0.00 mW cm⁻² (mean ± SEM). Insets highlight characteristic pDawn-*fliA* border morphologies. (**C**–**E**), Quantified features, extracted using binary colony segmentation masks, show gradual analog-like changes with increasing blue-light irradiance for pDawn-*fliA*, while remaining relatively constant for the pDawn-*mCherry* control. Points represent the mean of *n* = 11–12 independent biological replicates (swarm colonies) collected across ≥3 independent experiments per condition performed on different days. Error bars represent SEM. Asterisks denote significant pairwise differences among illumination conditions within the pDawn-*fliA* group only, determined by one-way ANOVA with Tukey–Kramer post hoc tests (*P* ≤ 0.01). Exact *P* values for all pairwise comparisons in both strains are reported in Appendix Table S8. **C** Minimum colony radius calculated from polar-coordinate colony masks by identifying the shortest distance from the inoculation point to the colony edge across angular columns. **D** Global colony intensity CV computed from pixel intensities within the segmented colony region, where higher CV reflects greater internal heterogeneity in colony density. **E** Mean circumferential intensity CV, reflecting asymmetry in internal density or global shape, computed from polar-coordinate images on a row-wise basis and averaged across circumferential rows. Source data are available online for this figure.

To validate light responsiveness, we first placed *mCherry* under the control of pDawn’s λ pR promoter, which blue light activates through a derepression cascade involving a chimeric two-component system. When exposed to blue light in liquid culture, MG1655hm harboring pDawn-*mCherry* produced a duration-dependent increase in fluorescence, with fold changes rising from ~5 after 1 h of illumination to ~64 after 24 h of illumination (Appendix Fig. S5A). We next replaced *mCherry* with *fliA*—which in the IPTG-inducible system produced a strong graded morphological response and enabled accurate decoding of inducer levels—to test whether it could similarly record varying levels of blue light (Fig. 6A).

Under non-inducing dark and red-light conditions, 24-h pDawn-*fliA* colonies were expansive but lacked the fine branching structures and pronounced radial striations of the *mCherry* control (Fig. 6B). Rather, pDawn-*fliA* formed smoother, clover-like morphologies with speckled lobes, suggesting mild leakiness of light-regulated *fliA* expression. With increasing blue-light irradiance, pDawn-*fliA* colonies became correspondingly smaller and denser, consistent with the trends observed for pLac-*fliA* under increasing IPTG induction. Under low blue light, pDawn-*fliA* colonies resembled the 1 mM IPTG pLac-*fliA* phenotype, exhibiting its characteristic undulating, lobate border. Under high blue light, pDawn-*fliA* colonies were slightly more compact than those of pLac-*fliA* at 10 mM IPTG, though they lacked the latter’s distinctive, sharply serrated peripheral structures. This difference was consistent with the stronger liquid-culture induction of pDawn-*mCherry* compared to pLac-*gfp*. The stronger gene expression in the pDawn strains may reflect the combined effects of high-intensity blue light and the reported high strength of the λ pR promoter, or other elements of the pDawn system.

To quantitatively assess blue-light–induced colony changes, we analyzed the pDawn image dataset using our endpoint image analysis pipeline unmodified—from colony segmentation to feature extraction and analysis—demonstrating its utility and generalizability (Appendix Fig. S5B). Representative colony features captured how pDawn-*fliA* (and not pDawn-*mCherry*) recorded blue-light irradiance in an analog manner. For example, minimum colony radius decreased progressively with increasing blue-light intensity (Fig. 6C). Global intensity CV increased with blue light, consistent with enhanced density contrast between regions near the inoculum and those closer to the swarm front (Fig. 6D). Mean circumferential intensity CV likewise increased with illumination intensity, indicating measurable changes in density variation along concentric circumferences throughout the colony (Fig. 6E). Together, these results show that programmable, swarming *E. coli* can record graded optogenetic activation of *fliA* into distinct, quantifiable swarm morphologies, extending the platform to an orthogonal input modality.

## Discussion

Leveraging the genetic tractability and swarming behavior of *E. coli*, we have presented a programmable visual system that records external information and communicates it through macroscale colony patterns. The selected *E. coli* K-12 MG1655 variant anchors the framework in a canonical model that is readily adoptable and adaptable within the synthetic biology community (Pontrelli et al, 2018; Browning et al, 2023). Our swarming system illustrates how the engineering of emergent microbial behaviors can draw on insights from regulatory networks underlying collective motility for practical applications. By swapping simple swarming-gene cassettes, we generated strains with analog- and binary-like spatiotemporal responses, tailoring colony outputs to specific recording and decoding needs.

As a preliminary test of functional modularity, we replaced the IPTG-inducible *lac* operon with the chimeric blue-light-inducible pDawn system. Because optogenetic systems have been applied to control bacterial behaviors with high spatial and temporal precision in applications ranging from microbial therapeutics to engineered living materials, our results suggest that swarming-based spatial recording—and more broadly, collective behaviors—could be incorporated into such platforms to provide an additional macroscale readout of cellular responses (Jin and Riedel-Kruse, 2018; Magaraci et al, 2014; Yang et al, 2020; Wang et al, 2021). Successful swarm-based recording of both chemical and optogenetic inputs highlights the flexibility and potential of the platform to interface with diverse signals of environmental, clinical, and materials relevance (Doshi et al, 2023; Liu et al, 2022; Chen et al, 2021). The availability of well-characterized and application-specific *E. coli* sensor circuits, combined with our swarming platform, thus offers a new spatial readout strategy for biosensing and, more broadly, precise control of cellular behaviors.

Complementing the flexibility of the swarm-based recording platform, our framework supports multiple computational decoding strategies. Regression models rely on explicit morphological descriptors, linking individual colony features to predictive performance and providing interpretable insight into how regulatory perturbations shape swarm morphologies. Such interpretable features could inform future efforts to engineer swarm phenotypes through targeted genetic circuit design. In contrast, deep-learning models learn predictive representations directly from colony images without predefined feature extraction, potentially simplifying deployment in automated imaging pipelines or mobile platforms. Deep-learning models may also generalize more readily across variations in imaging conditions, although they typically require larger datasets and greater computational resources for training. Extending our framework to continuous prediction across intermediate or previously unseen input levels represents an important direction for future work and could further expand the quantitative capabilities of swarm-based information decoding. Together, the various computational approaches involve trade-offs between interpretability, flexibility, and scalability; feature-based and deep-learning models can therefore play complementary roles in biological information-decoding systems.

Compared with traditional electronic and analytical sensors, swarm-based biosensing offers unique capabilities and constraints. Electronic sensors typically provide rapid and continuous measurements, whereas swarm colony development occurs over longer timescales. While this slower response limits real-time detection, it creates the potential for colony morphology to integrate environmental inputs over the course of growth, producing a cumulative record of the conditions encountered during development. The system could be adapted for faster readouts by tuning growth conditions (e.g., lower agar density or increased surfactants), and for threshold-based ON/OFF readouts through circuit design, with more quantitative decoding possible over longer timescales. Importantly, swarming generates centimeter-scale colony patterns that are durable and visually distinct, making them interpretable and accessible using only standard Petri dishes and simple imaging devices rather than specialized analytical instrumentation. In certain scenarios, swarming readouts could be monitored in situ through direct or remote imaging without requiring sample collection and transfer to a centralized analytical facility. In addition, the large populations of cells contributing to swarm pattern formation—often organized in radially symmetric expansions—provide repeated measurements across space, in contrast to single-point measurements, which may enhance robustness and signal-to-noise in decoded outputs.

Beyond the inherent strengths of swarming, the unparalleled breadth of genetic tools in *E. coli* opens avenues to expand our platform’s capabilities. Further circuit engineering—such as tuning promoters and ribosome binding sites or chromosomally integrating constructs—combined with predictive computational design could refine input sensitivity and dynamic range (Tica et al, 2024; Tyson et al, 1999; Macklin et al, 2020; Wang et al, 2020; LaFleur et al, 2022). Logic frameworks enable opportunities for multiplexed biosensing (Nielsen et al, 2016; Jones et al, 2022; Meyer et al, 2019); chromoproteins, knockdowns, knockouts, or other swarming-related genes could expand outputs for visualization and quantification (Liljeruhm et al, 2018; Kitagawa et al, 2005; Inoue et al, 2007). The hallmarks of swarming—its robustness, scalability, and emergent macroscale morphologies—underpin its potential for broader application, from remote environmental monitoring and education to the self-assembly of functional materials (Chemla et al, 2025; Huang et al, 2018; Rodrigo-Navarro et al, 2021). By rendering complex collective behaviors visible and tractable, engineered *E. coli* swarming provides both a framework for studying emergent biological phenomena and a platform for programmable information recording.

## Methods

**Table:** Reagents and tools table

Reagent/resource	Reference or source	Identifier or catalog number
**Experimental models**		
MG1655 hypermotile (hm)	Coli Genetic Stock Center	8237
Mach1 Competent Cells	Thermo Fisher Scientific	C862003
**Recombinant DNA**		
pLac-*gfp*	Doshi et al, (2023)	N/A
pLac-*lrp*	This study	N/A
pLac-*fliA*	This study	N/A
pLac-*rpoS*	This study	N/A
pLac-*cheZ*	This study	N/A
pDawn-*sfGFP*	Addgene	107741
pDawn-*mCherry*	This study	N/A
pDawn-*fliA*	This study	N/A
**Antibodies**		
**Oligonucleotides and other sequence-based reagents**		
Gibson assembly primers	This study	Appendix Table S2
**Chemicals, Enzymes and other reagents**		
LB broth (Lennox)	Fisher Scientific	BP9722-500
LB agar (Lennox)	Thermo Fisher Scientific	22700025
D-glucose (dextrose), anhydrous	Fisher Scientific	D16-500
Agar (Eiken)	GERBU	EMJ00
IPTG	Thermo Fisher Scientific	FERR0392
Kanamycin sulfate	MilliporeSigma	PHR1487-500MG
EDTA (0.5 M, pH 8.0)	MilliporeSigma	324504-500 ML
Sodium chloride	MilliporeSigma	RDD041-500G
Lysozyme (from chicken egg white)	MilliporeSigma	L6876-5G
*N*-Lauroylsarcosine sodium salt solution	MilliporeSigma	L7414-10ML
Phenol solution	MilliporeSigma	P4682-100ML
Phenol:Chloroform:iso-amyl alcohol	MilliporeSigma	P1944-400ML
Sodium acetate (3 M, pH 5.2)	MilliporeSigma	567422-100 ML
Glycerol (molecular biology grade)	Thermo Fisher Scientific	BP229-1
SOC medium	Thermo Fisher Scientific	15544034
DNase/RNase-free water	Thermo Fisher Scientific	10977015
Q5 Hot Start High-Fidelity 2X Master Mix	New England Biolabs	M0494S
NEBuilder HiFi DNA Assembly Master Mix	New England Biolabs	E2621S
**Software**		
Epson Scan II	Epson	v6.6.45
Vsensor (mobile application)	VABIRA	v1.2.6
Adobe Photoshop	Adobe	v27.2.0–27.5.0
Adobe Premiere Pro	Adobe	v25.6.4
AppleScript (custom code)	This study	N/A
i-control	Tecan	v2.0.10.0
Python	Python Software Foundation	N/A
TensorFlow	Google	v2.15.0
PyTorch	PyTorch Foundation	v2.8
torchvision	PyTorch Foundation	v0.23
MATLAB	MathWorks	R2020a–R2024b
tfswin	Python Package Index	v3.4.0
**Other**		
Petri dishes (100 mm × 15 mm)	VWR	25384-094
Biosafety cabinet (Class II, Type A2)	Labconco	302411101
LED grow panel (red, 660 nm, 14 W)	HQRP	N/A
LED grow panel (blue, 465 nm, 14 W)	HQRP	N/A
Black foam board	Royal Brites	N/A
Blue-light irradiance meter (VBR-Blue)	VABIRA	VBR-Blue
Scanner (Epson Perfection V850 Pro)	Epson	B11B224201
Scanner (Epson Perfection V19 II)	Epson	J371B
Incubator	VWR	89511-420
NanoDrop One spectrophotometer	Thermo Fisher Scientific	13-400-518
MicroPulser electroporator	Bio-Rad	1652100
Tecan Infinite 200 PRO M Plex	Tecan	30190085
QIAprep Spin Miniprep Kit	Qiagen	27106

### Experimental procedures

#### Bacterial strains, storage, and growth conditions

*E. coli* MG1655(motile) (Coli Genetic Stock Center; CGSC #8237) was used as the host swarming strain and is referred to as hypermotile MG1655 (MG1655hm) in this study. This strain is an isolate of MG1655 (CGSC #6300) that was selected and verified for motility. Previous studies have reported that CGSC #8237 and other motile *E. coli* K-12 derivatives carry an IS5-element insertion upstream of the *flhD* promoter, which activates transcription of the *flhDC* master regulator of flagellar biosynthesis and motility, thereby increasing swarming motility (Barker et al, 2004; Pleška et al, 2021). Additional MG1655 strains used in preliminary optimization experiments (parent, Δ*lacI*, and Z1; Appendix Fig. S1A) were obtained from laboratory stocks. *E. coli* Mach1 (Thermo Fisher) was used for cloning IPTG-inducible swarming plasmids.

Strains were stored in 25% glycerol at −80 °C. For liquid culture, strains were grown in Luria–Bertani (LB) broth. For solid culture (i.e., for isolation of clonal, non-motile colonies), strains were grown on LB agar. For swarming experiments, strains were inoculated onto semi-solid media comprised of 0.45% (w/v) Eiken agar, 0.5% anhydrous D-glucose (Thermo Fisher), and 2% LB broth, with minor changes in optimization experiments as noted. All LB broth and LB agar used in this study were Lennox (Thermo Fisher). Media were supplemented with IPTG and 50 μg mL^−1^ kanamycin as needed. For experiments involving the blue-light-inducible pDawn system, liquid cultures were grown under dark conditions by wrapping culture tubes in aluminum foil to prevent unintended light induction. Details on specific culturing protocols are below.

#### Electrocompetent cell preparation

MG1655hm and Mach1 cells were made electrocompetent as follows. Cells from −80 °C glycerol stocks were inoculated into 2 mL LB broth and grown at 37 °C with shaking overnight (around 16 h). The fresh overnight culture was subcultured 1:100 in 50 mL LB broth and grown at 30 °C with shaking for around 2.5 h, or until cells reached logarithmic growth phase, indicated by an optical density at 600 nm (OD_600_) of 0.3–0.8. Growth was halted by incubation of the culture on ice for 15 min. Cells were then pelleted via centrifugation at 4 °C and 3000×*g* for 10 min. After decanting the supernatant, the pellet was resuspended in 50 mL ice-cold filter-sterilized water and centrifuged again under the same conditions. This washing process was repeated twice more. Following the fourth round of centrifuging and decanting, the pellet was resuspended in 220 μL 10% glycerol. Lastly, 50 μL aliquots were stored at −80 °C. The serological pipettes, micropipette tips, conical centrifuge tubes, and filter-sterilized water used for washing were pre-chilled at −20 °C. The 10% glycerol was pre-chilled at 4 °C. The microcentrifuge tubes and micropipette tips used for aliquoting were pre-chilled at −80 °C.

#### DNA preparation

Plasmid DNA was purified from 5 mL bacterial overnight cultures using the QIAprep Spin Miniprep Kit and its accompanying protocol (Qiagen). Elution of plasmid DNA was done in UltraPure™ DNase/RNase-Free Distilled Water (Thermo Fisher). MG1655 genomic DNA was extracted using the following protocol adapted from standard bacterial DNA extraction methods. A fresh 2 mL overnight culture of MG1655 was transferred to an Eppendorf DNA LoBind^®^ Tube and centrifuged at 4 °C and 3000×*g* for 5 min. The supernatant was decanted, and the pellet was resuspended in 500 μL lysis buffer (50 mM EDTA, 0.1 M NaCl, and pH 7.5). Freshly prepared 12 mg mL^−1^ lysozyme solution was added (100 μL), followed by incubation in a 37 °C water bath for 10 min and treatment with 38 μL 20% Sarcosyl (*N*-Lauroylsarcosine sodium salt solution). The lysate was then subjected to two sequential extractions—first with 500 μL phenol solution, second with 500 μL phenol:chloroform:iso-amyl alcohol (125:24:1)—and centrifugation at 4 °C and 17,000×*g* for 10 min. The aqueous phase was collected, mixed with 50 µL 3 M sodium acetate (pH 5.2) and 1 mL 95% ethanol, and centrifuged at 4 °C and 21,100×*g* for 5 min. The pellet was similarly washed with 500 μL 70% ethanol, air-dried, and resuspended in 200 µL nuclease-free water. DNA purity and concentration were measured using a NanoDrop spectrophotometer. Short- and long-term storage of DNA preparations were at 4 °C and −20 °C, respectively.

#### Genetic circuits

IPTG-inducible swarming plasmids were derived from pZE24, a high-copy plasmid (ColE1 origin of replication) with a kanamycin resistance cassette and the IPTG-inducible promoter pLac (Lutz, 1997). The vector was previously modified for pLac-control of *gfp* and constitutive overexpression of the LacI repressor (Doshi et al, 2023). The resulting pLac-*gfp* pConst-*lacIQ* (referred to as pLac-*gfp* in this study) was the control plasmid and backbone for IPTG-inducible swarming plasmids. Swarming-related gene sequences (*cheZ*, *lrp*, *fliA*, and *rpoS*) were obtained from the *E. coli* K-12 MG1655 complete genome sequence (Appendix Table S1; Data ref: NCBI Reference Sequence: NC_000913.3; Blattner et al, 1997). These genes were amplified from MG1655 genomic DNA via polymerase chain reaction with custom-designed Gibson primers (Eton Bioscience, Inc.) and Q5^®^ Hot Start High-Fidelity 2X Master Mix (New England Biolab, NEB; Appendix Table S2). The backbone of pLac-*gfp* was similarly amplified, removing *gfp*. Swarming genes were inserted into the backbone via Gibson Assembly (NEBuilder^®^ HiFi DNA Assembly Master Mix), creating a set of pLac-[*swarming gene*] plasmids. After cloning plasmids into Mach1 electrocompetent cells (following the electroporation protocol below), select clones were grown in liquid culture at 37 °C with shaking overnight. From these overnight cultures, −80 °C glycerol stocks were stored, and plasmids were prepared and verified via whole-plasmid sequencing (Plasmidsaurus).

Blue-light-inducible plasmids were derived from pDawn-*sfGFP*, a gift from Ingmar Riedel-Kruse (Addgene plasmid #107741; Jin and Riedel-Kruse, 2018). Like the IPTG-inducible pLac plasmids used in this study, pDawn-*sfGFP* carries a ColE1-derived origin of replication and confers kanamycin resistance. Using GenScript’s Premium Gene Synthesis service, *sfGFP* was replaced with *mCherry* and *fliA* via CloneEZ® cloning in *E. coli* TOP10 competent cells. The original vector (pDawn-*sfGFP*) and the synthesized plasmids (pDawn-*mCherry* and pDawn-*fliA*) were verified by full-length Nanopore sequencing (GenScript).

#### Electroporation

Plasmid DNA was introduced into electrocompetent *E. coli* cells as follows. Fifty-microliter aliquots of electrocompetent cells were thawed on ice for 10 min. DNA was added to the thawed cells (exactly 2 μL Gibson Assembly product per Mach1 aliquot; ~80–100 ng plasmid DNA in a volume of around 0.2–0.7 μL per MG1655hm aliquot). The mixture of cells and DNA was transferred to a pre-chilled 0.1 cm–gap electroporation cuvette and pulsed with a Bio-Rad MicroPulser set to the E1/bacteria program (1.8 kV). Electroporated cells were recovered in 1 mL prewarmed SOC (super optimal broth with catabolite repression) medium (Thermo Fisher) and incubated at 37 °C with shaking for 1 h. Cells were pelleted via centrifugation at 4 °C and 3000×*g* for 10 min. A portion of the supernatant was decanted (750 μL for Mach1; around 200–500 μL for MG1655hm), and cells were resuspended in the remaining volume. Resuspended cells were plated on prewarmed LB agar plates with kanamycin and incubated at 37 °C for 16–24 h. Single colonies were inoculated into 2 mL LB broth with kanamycin and grown at 37 °C with shaking overnight. Fresh overnight cultures were stored in 25% glycerol at −80 °C.

#### Liquid-culture induction assays

The induction-response data analyzed in Appendix Figs. S1B,D and S5A were collected in 96-well plates (Corning™ 96-Well Clear Bottom Black Polystyrene Microplate) with a Tecan Infinite 200 PRO® M Plex using the i-control™ software version 2.0.10.0. For the microplate-reader experiment presented in Appendix Fig. S1B,D, fresh 2 mL overnight cultures of MG1655hm wild type and pLac-*gfp* were diluted 100-fold with LB broth in a 96-well plate. Specifically, 1 μL of each culture was inoculated into triplicate wells, each containing 99 μL LB broth supplemented with kanamycin and IPTG as necessary. The plate was incubated in the Tecan at 37 °C for 18 h. Absorbance at 600 nm (OD_600_) and GFP fluorescence (excitation/emission: 488/520 nm) were measured every 10 min following 300 s of orbital shaking (4.5 mm amplitude).

To measure mCherry expression in liquid culture in response to varying durations of blue-light induction (Appendix Fig. S5A), a protocol from Ohlendorf et al was modified as follows (Ohlendorf et al, 2012). Fresh overnight cultures of MG1655hm pDawn-*mCherry* and pDawn-*fliA* were subcultured 1:100 in 40 mL LB with kanamycin and grown in the dark at 37 °C with shaking for around 2 h, or until cells reached logarithmic growth phase (OD_600_ = 0.3–0.8). Cultures were then distributed into 2 mL aliquots and exposed to blue light at 37 °C by placement above a 465 nm LED panel (74.75 ± 2.24 mW cm^−2^, mean ± SEM; see “pDawn swarming assays” below for setup details). After 0, 1, 2, 3, 4, and 24 h illumination, aliquots were returned to non-inducing dark conditions and grown at 37 °C with shaking for a total culture duration of 24 h. Cultures were then transferred in triplicate to a 96-well plate, from which OD_600_ and mCherry fluorescence (excitation/emission: 561/610 nm) were measured with the Tecan.

#### Swarming assays

Cells from −80 °C glycerol stocks were inoculated into 2 mL LB broth supplemented with kanamycin as needed. The liquid cultures were incubated at 37 °C with shaking overnight, then maintained at room temperature throughout the following experimental setup. The swarming assay protocol was adapted from published methods (Doshi et al, 2023; Bhattacharyya et al, 2023; Partridge et al, 2019); standard conditions for this study were optimized by varying host strain, medium formulation and preparation, and incubation conditions.

Swarming medium consisting of 0.45% Eiken agar, 0.5% D-glucose, and 2% LB broth was freshly prepared, cooled to 55 °C with stirring, then supplemented with kanamycin and IPTG as appropriate. In a Class II, Type A2 Biosafety Cabinet (BSL-II), 20 mL of the solution was poured into each Petri dish (100 mm diameter × 15 mm height). Uncovered agar dishes were left to solidify in the cabinet for 90 min. Toward the end of agar solidification, the OD_600_ of each overnight bacterial culture was measured and normalized to 1.0 by dilution in LB broth. Two microliters of the diluted culture were inoculated onto the center of each solidified agar dish and left to dry uncovered for 15 min in the BSL-II cabinet. Finally, dishes were incubated upside down with lids on (except in time-lapse assays as described below) at 37 °C for 24 h. The relative humidity of the incubator was maintained around 75%.

#### pDawn swarming assays

Swarming assays involving the blue-light–inducible pDawn system were prepared and conducted as above, with additional illumination conditions applied during 37 °C incubation. Freshly inoculated Petri dishes were placed on a metallic, perforated incubator shelf ~9.5 inches above an HQRP LED Plant Grow Panel Lamp System (12” × 12” panel with 225 single-wavelength LEDs, 14 W total output). Illumination reached the dishes through the shelf perforations, gaps between the shelf and incubator walls, and scattering from the metallic interior of the incubator. For blue-light induction, a blue LED panel (465 nm wavelength) was used.

High and low blue-light conditions were defined by the measured blue-light irradiance at the dish surface, quantified using a blue-light–specific irradiance meter sensitive to wavelengths in the 400–500 nm range (VBR-Blue Blue Light Meter). High blue-light conditions corresponded to direct illumination at the fixed ~9.5-in distance (74.75 ± 2.24 mW cm^−2^, mean ± SEM), whereas low blue-light conditions were achieved by attenuating incident light using opaque shielding placed between the LED panel and the incubator shelf, thereby reducing irradiance while maintaining identical incubation geometry and temperature conditions (22.78 ± 1.28 mW cm^−2^).

To provide non-inducing control conditions, cultures were incubated either in the dark or under red-light illumination using the same HQRP LED panel system equipped with 660 nm LEDs, which mimics potential thermal effects from LED operation while avoiding blue-light activation. Dark conditions measured 0.00 ± 0.00 mW cm^−2^. Red-light illumination fell outside the spectral sensitivity of the irradiance sensor and was therefore treated as a non-inducing condition. LED panels remained on continuously for the full 24-h incubation period.

#### Swarm-colony imaging

After incubation at 37 °C for the specified duration, Petri dishes were removed from the incubator and individually imaged with an Epson Perfection V850 Pro Photo Scanner at ambient temperature and lighting (i.e., on the benchtop). Dishes were placed on the scanner glass colony-side up with lids off. Using the Epson Scan II application, a 3.54” × 3.54” field of view was centered tightly around each dish. Individual scans were acquired at 48-bit color depth and 400 dots per inch (dpi) with high scanning quality and saved as TIFF files.

#### Time-lapse swarming assays

Swarming dishes were prepared as described above. After inoculums dried, uncovered dishes (six at a time) were set inoculum-side up on the scanner glass of a small scanner placed inside the 37 °C incubator (Epson Perfection V19 II Color Photo and Document Flatbed Scanner). The scanner cover was removed, and the exposed dishes were covered with 11” × 14” black foam board to minimize reflections from the metallic incubator walls during scanning. A custom AppleScript, previously written for the benchtop Epson Perfection V800 and V850 Pro (Doshi et al, 2023), was adapted to program the V19 II to scan dishes every 10 min for 24 h (user-definable settings) at 37 °C. Time-lapse scans were acquired at 24-bit color depth and 400 dpi with high scanning quality and saved as TIFF files.

For the localized IPTG-perturbation experiment, time-lapse acquisition was performed as above, except that scans were acquired at 300 dpi. Approximately 12 h after the start of time-lapse acquisition, 2 µL of 1 M IPTG was manually pipetted onto the agar immediately adjacent to the colony perimeter, just outside the expanding swarm front. Imaging continued uninterrupted under identical scanning conditions for the duration of the experiment.

### Computational analysis

All image datasets were collected across multiple independent experiments conducted over several months, with colonies grown on different days and Petri dishes under consistent protocols. No statistical methods were used to predetermine sample sizes; Petri dishes were typically generated in groups of three or more biological replicates per condition, and the number of independent swarm colonies analyzed is indicated in the corresponding figure legends. Accordingly, model training, validation, and test dataset splits contain samples spanning multiple experimental days, providing inherent biological variability. Unless otherwise noted, measurements were taken once from each independent sample (colony dish or liquid culture), with no repeated measurements from the same sample. No covariates were tested in any analyses. No blinding was performed, as colonies were imaged under standardized conditions and quantitative analyses were conducted using code-based methods with only minimal user input (e.g., occasional colony mask refinement). Data were excluded only when Petri dishes showed clear technical artifacts (e.g., uneven antibiotic or inducer distribution or uneven agar solidification) that could confound feature extraction; these criteria were based on technical quality and applied consistently across experiments, with all remaining data included in the analyses. *P* values were annotated throughout as ns (*P* > 0.01), * (*P* ≤ 0.01), ** (*P* ≤ 10^−^³), *** (*P* ≤ 10^−^⁴), or **** (*P* ≤ 10^−^⁵).

Image processing, feature extraction, statistical analyses, and plotting were done in MATLAB R2020a–R2024b (MathWorks) using custom-written, built-in, and open-source functions. Deep-learning classification models were implemented in Python using TensorFlow (v2.15.0) with the tfswin package (v3.4.0) (Shkarupa, 2022) for single-strain models, and PyTorch (v2.8) with the torchvision library (v0.23) for the unified multiplexed models. Associated learning curves were plotted in Python, and all other visualizations were generated in MATLAB.

#### Liquid-culture induction analysis

The fluorescence analyses presented in Appendix Figs. S1B and S5A were conducted as follows. Raw OD_600_, GFP, and mCherry readings from the Tecan plate reader were first background-subtracted using media-only blank wells. Any resulting values ≤ 0 were replaced with a small offset (ε = 0.001) to enable downstream normalization and, for growth-rate analysis, log transformation. Preprocessed GFP and mCherry values were normalized to their corresponding preprocessed OD_600_ values. Autofluorescence was corrected by subtracting the mean normalized fluorescence of wild type and pDawn-*fliA* from the corresponding normalized fluorescence of pLac-*gfp* and pDawn-*mCherry* at each IPTG concentration and blue-light illumination duration, respectively. mCherry data are presented as such in Appendix Fig. S5A (left), whereas the finer-grained pLac-*gfp* time-course data were further preprocessed.

Specifically, the background-corrected fluorescence trajectories of pLac-*gfp* were clipped to exclude the first 3 h, during which fluorescence measurements were noisy, then smoothed using a moving average (window size = 5; Appendix Fig. S1B, top). Endpoint fluorescence was defined as the final time point of the smoothed trajectory (~18 h; Appendix Fig. S1B, middle). GFP and mCherry fold changes were calculated by normalizing each replicate’s endpoint fluorescence to the mean endpoint fluorescence of the non-induced replicates (Appendix Fig. S1B, bottom; Appendix Fig. S5A, right). To compare fluorescence responses across the different IPTG concentrations and illumination durations, one-way analysis of variance (ANOVA) was performed at a significance level (α) of 0.01. Tukey–Kramer post hoc tests (*multcompare*, α = 0.01) were performed to assess pairwise differences between induction conditions, with the resulting *P* values shown in the plots.

For the growth rate analysis presented in Appendix Fig. S1D, OD_600_ trajectories were preprocessed as described above, log-transformed, and smoothed using a moving average (window size = 5). Growth rate was defined as the maximum slope of the smoothed, log-transformed OD_600_ curve after 1 h, corresponding to the exponential growth phase. Relative growth rates were determined by normalizing each replicate’s growth rate to either (1) the mean growth rate of pLac-*gfp* at the same IPTG concentration (Appendix Fig. S1D, left), or (2) the strain’s own mean growth rate at 0 mM IPTG (Appendix Fig. S1D, right).

#### Colony segmentation

To facilitate feature extraction, a custom automated MATLAB script was written to approximate colony segmentation masks as follows. Original RGB scans of swarm dishes were converted to grayscale, and the dish rim was masked out using *createCirclesMask* (MATLAB File Exchange). The image contrast was then enhanced through contrast-limited adaptive histogram equalization (*adapthisteq*). From the enhanced grayscale image, a global threshold was computed using Otsu’s method via the *graythresh* function. The found threshold was then used with *imbinarize* to convert the rimless grayscale image to a binary image, creating an initial colony mask approximation. The colony segmentation was improved through a series of postprocessing operations involving connected component analysis, dilation, hole-filling, and erosion. Select colony masks were finalized via minor manual refinement in Adobe Photoshop as necessary.

#### Colony feature extraction

Another custom semi-automated MATLAB script was written to extract features from swarm colony images with the aid of colony masks, requiring minimal user interaction. In brief, original RGB colony images were converted to grayscale and lightly denoised with an adaptive 9 × 9-pixel low-pass Wiener filter. Denoised colonies were segmented with their final colony masks and converted to both the frequency domain and polar coordinates—transformations that required the user to interactively locate the inoculum center with a click of the mouse. To generate a colony’s Fourier (frequency) transform, the denoised colony image was first cropped with a 180 × 180-pixel box centered around the inoculum. The two-dimensional fast Fourier transform of the cropped image was computed (*fft2*), followed by centering the zero-frequency component (*fftshift*), suppressing the DC component, and taking the magnitude of the spectrum for analysis. For visualization in figures, the natural logarithm of the magnitude spectrum was computed and normalized to [0, 255].

To generate a colony’s polar transform, a previously implemented method was adapted (Doshi et al, 2023). Briefly, the denoised colony image and its corresponding binary mask (with the background agar and dish rim blacked out) were “unrolled” around the inoculum using a Cartesian-to-polar transformation, nearest neighbor interpolation, and re-mapping to a 1000 × 1000-pixel grid (or to a proportionally larger grid for pLac-*lrp* colonies that extended to the dish edge). In the resulting polar image, the inoculum center spans the top; traversing rows downward corresponds to radial projections, and traversing columns laterally corresponds to circumferential projections, with any masked regions shown in white. The polar representations reduced the computational burden of extracting certain features compared with doing so from Cartesian images.

From these three preprocessed colony representations (Cartesian, polar, and Fourier), an expansive list of features was automatically extracted using colony masks along with MATLAB’s built-in *regionprops* and additional custom-written functions as needed. For Fourier-domain features, extracted from the cropped 180 × 180-pixel transforms described above, frequency subsets were defined using disk-shaped masks: a radius of 18 pixels (10% of Fourier image side length) captured low frequencies, and a radius of 45 pixels (25%) captured low–intermediate frequencies. Magnitudes within each mask were summed and, when appropriate, normalized to the total spectral magnitude. Measurements with units of length and area were converted from pixels to centimeters based on the 400-dpi image resolution. For each strain–induction condition (i.e., IPTG for pLac strains; light condition for pDawn strains), feature means and standard errors of the mean (SEM) were calculated, and normality was assessed using the Anderson-Darling test (*adtest*, α = 0.01). Representative significant features were plotted as mean ± SEM.

#### Statistical analysis of colony features

*P* values indicating statistically significant differences between IPTG-dependent feature response curves were determined by fitting linear mixed-effects models (*fitlme*) for each feature within each pLac strain set: binary (pLac-*lrp* and its corresponding pLac-*gfp* control) and analog (pLac-*fliA* and pLac-*rpoS* with their corresponding pLac-*gfp* controls). In all models, pLac-*gfp* served as the reference strain. The model specification was Value ~ Strain * IPTG + *(1|UniqueRep)*, where *Strain*, *IPTG*, and their interaction were fixed-effects terms, and *UniqueRep* (independent biological replicate identity) was a random-effects term. Models included only these experimental factors of interest; no additional covariates were tested. Selection between raw and log_10_-transformed IPTG concentrations was based on minimizing the model’s Akaike Information Criterion. Across all analyzed features, raw IPTG values were optimal for binary-strain models, whereas log_10_-transformed IPTG values (offsetting 0 mM IPTG to 10^−2 ^mM) were optimal for analog-strain models. The overall significance of the Strain × IPTG interaction for each feature and strain set was determined by performing ANOVA on the fitted models (effectively, a two-way ANOVA with interaction). For the analog strains, post hoc pairwise comparisons of IPTG-dependent feature response curves were conducted by cyclically refitting the model with each strain as the reference, then testing the corresponding interaction coefficient using *coefTest*. For consistency, IPTG axes in all figures were plotted on a log scale, even when raw values were used for pLac-*lrp* models. For the pDawn system, statistical differences in colony features across strain ×  illumination groups were assessed using one-way ANOVA followed by Tukey–Kramer post hoc tests (α = 0.01).

#### Regression models

Feature selection and regression model fitting were performed independently for each engineered strain and its corresponding pLac-*gfp* control to account for strain-specific IPTG–phenotype relationships. Binary IPTG prediction models were implemented for the pLac-*lrp* strain and its pLac-*gfp* control, whereas multiclass (ordinal) IPTG prediction models were implemented separately for the analog strains pLac-*fliA* and pLac-*rpoS* and their respective controls. Within each strain–control pair, redundant or non-informative features were removed prior to model fitting (Datasets EV1 and EV2). The resulting strain-specific feature subsets were split into stratified, random training (70%) and test (30%) sets (Appendix Table S4). All possible combinations of 1–4 features were generated for model fitting.

For binary IPTG prediction, binomial generalized linear regression models were fit to pLac-*lrp* training combinations using a Jeffreys prior likelihood penalty (*fitglm*). For multiclass IPTG prediction, ordinal multinomial regression models were separately fit to pLac-*fliA* and pLac-*rpoS* training combinations (*fitmnr*). Various standard link functions were evaluated (logit, probit, comploglog, and loglog). Fitted models were evaluated on the held-out test sets (α = 0.01) using accuracy (fraction of correctly classified samples) and the area under the receiver operating characteristic curve (ROC–AUC), which quantifies the model’s ability to discriminate between classes across decision thresholds. AUC was computed using MATLAB’s *perfcurve* function for binary models and *multiClassAUC* (MathWorks File Exchange) for multiclass models. For binary models, class predictions were obtained using a probability threshold of 0.5. Models were screened for significant coefficients (*P* ≤ α) and absence of fitting warnings. For each engineered strain, the model achieving the highest test AUC—consistently using the probit link function—was selected as optimal and is presented alongside the performance of its corresponding pLac-*gfp* control model (Appendix Table S5).

#### Deep-learning models

The classification pipeline was adapted from a previously described implementation of a Transformer model, the SwinTransformerTiny224 (Doshi et al, 2023; Liu et al, 2021). For each classification task described below, 24-h endpoint colony images were randomly shuffled and stratified to preserve class proportions before being split into training, validation, and test sets (70/15/15%), then resized to 224 × 224 pixels using bicubic interpolation. All models used a SwinTransformerTiny224 backbone (eleven layers excluding the default classification head) appended with a new classification head consisting of a Global Average Pooling layer and a Dense output layer. The number of output units in the Dense layer was one for single-strain binary models, five for single-strain multiclass models, and nine for dual-strain multiclass models. Sigmoid and softmax activation were used for binary and multiclass problems, respectively.

Models were trained with a batch size of 2 using the Adam optimizer (epsilon = 1 × 10^−8^) and cross-entropy loss. Class weights were applied to address dataset imbalance, and early stopping (patience = 4) was used based on validation loss. ImageNet-initialized (“IN”) models were evaluated using fully fine-tuned (“FFT”) and partially fine-tuned (“PFT”) configurations. Fully fine-tuned models had all layers trainable, whereas partially fine-tuned models had the first three layers frozen. On-the-fly data augmentation (“aug”) included horizontal flips, zoom-ins (+2 to +10%), contrast adjustments (−0.4 to +0.4), rotations (±1% × 2π), and horizontal translations (±10%), with empty regions filled using reflect mode. Performance was evaluated using accuracy, Top-2 accuracy (fraction of images for which the correct class appears among the two highest-probability predicted classes), and ROC–AUC, with binary or categorical formulations applied as appropriate. AUC was computed using TensorFlow’s tf.keras.metrics.AUC for binary and five-class single-strain models, and as a macro-averaged one-vs-rest score (roc_auc_score, scikit-learn) for dual-strain nine-class models.

#### Single-strain classification models

Separate model instances were trained to classify IPTG concentration from multiclass pLac-*fliA*, pLac-*rpoS*, and pLac-*gfp* image datasets, and from binary pLac-*lrp* and pLac-*gfp* image datasets. The primary pLac-*fliA*, pLac-*rpoS*, and pLac-*gfp* datasets (as used in the feature and statistical-based analyses above) were expanded with images acquired in supplemental experiments under the same conditions (Appendix Table S6). Pooled pLac-*gfp* images, representing nine IPTG concentrations, were binned to match the class structures of the engineered swarming strains. For the multiclass pLac-*fliA* and pLac-*rpoS* controls, pLac-*gfp* images were binned into five classes: (1) 0, (2) 0.025–0.05, (3) 0.1–0.3, (4) 0.5–1, and (5) 2.5–10 mM IPTG. For the binary pLac-*lrp* control, pLac-*gfp* images were binned into (1) 0–0.05 and (2) 0.1–10 mM IPTG. Both randomly initialized (“baseline”) and ImageNet-initialized (“IN”) backbones were evaluated. The learning rate was set to 1 × 10^−5^. For pLac-*lrp*, pLac-*fliA*, and pLac-*rpoS*, the model with the lowest validation loss was taken as optimal (Appendix Table S7). These three optimal model configurations were then used to train, validate, and test three corresponding pLac-*gfp* control models.

#### Dual-strain joint classification models

To evaluate whether a single model could simultaneously decode engineered strain identity and IPTG concentration, SwinTransformerTiny224 models were trained on pooled 24-h endpoint colony images of pLac-*fliA* and pLac-*rpoS*. Images were aggregated into a nine-class dataset, with all 0 mM IPTG images merged into a single baseline class (“no inducer present”). The remaining eight classes corresponded to strain-specific IPTG concentrations (pLac-*fliA*: 0.1, 0.3, 1, and 10 mM; pLac-*rpoS*: 0.025, 0.1, 2.5, and 10 mM). Due to the increased class complexity, only ImageNet-initialized fully and partially fine-tuned models with data augmentation were evaluated (“IN_FFT_aug” and “IN_PFT_aug”). The learning and dropout rates were set to 3 × 10^−5^ and 0.15, respectively. The configuration achieving the lowest validation loss was selected as optimal (Appendix Table S7). A corresponding nine-class pLac-*gfp* control model was then trained and evaluated using this same optimal configuration, with nine IPTG classes (0, 0.025, 0.05, 0.1, 0.3, 0.5, 1, 2.5, and 10 mM).

#### Time-lapse feature analysis

A custom high-throughput, semi-automated MATLAB script was developed for temporal feature extraction. Sequential colony masks were generated frame by frame through the additive inclusion of areas of growth. Mask accuracy was refined using image-processing methods (particle filtration, dilation, hole-filling, and contrast-limited adaptive histogram equalization), with minor manual adjustments in Adobe Photoshop as needed. From finalized masks, feature extraction proceeded as for 24-h endpoint images, excluding polar-coordinate and Fourier analyses. Texture-based features were determined using gray-level co-occurrence matrices with an offset of [5,5].

Statistical analysis of time-lapse features was conducted as described above for analog strains in “Statistical analysis of colony features”. Time windows began at 5 h, when colonies reached centimeter-scale expansion. The exact statistical powers of two-sample, two-tailed *t*-tests (for pairwise feature comparisons of 0 and 10 mM IPTG at distinct timeframes) were calculated using the *extpowerStudent* function (MathWorks file exchange; α = 0.05), which employs the noncentral cumulative distribution function. Representative movies of raw time-lapse images were compiled in Adobe Premiere Pro (Movies EV1 and EV2). Snapshots from time-lapse sequences shown in Figs. 5A and EV5A were visually enhanced in Adobe Photoshop solely for presentation purposes, with no effect on analyses. Enhancements included a dust and scratches filter (radius = 5 pixels, threshold = 10 levels), reverse perceptual gradient mapping, and brightness/contrast adjustment (−37/+100).

#### Localized IPTG-perturbation analysis

A custom semi-automated MATLAB script was written to quantify directional changes in swarm colony development pre- and post-localized IPTG addition. Colony segmentation of time-lapse image sequences was performed as described above. For each swarm colony (biological replicate), a late time point (~22 h)—at which agar remained visible between the colony edge and dish rim—was used to define directional reference axes. The user interactively selected the inoculum center and extended a line from the inoculum through the site of IPTG addition. Using this reference direction, two angular wedge masks (50° each) were defined in polar coordinates centered at the inoculum: an IPTG-facing wedge and a diametrically opposite wedge (180° offset).

For time-resolved feature extraction, the wedge masks were applied to colony masks at each time point. Colony perimeters were identified using *bwperim*, and Euclidean distances from the inoculum center to perimeter pixels within each wedge were computed using *hypot*. The minimum radius was extracted for each wedge at each time point, and distances were converted from pixels to centimeters using the appropriate 300-dpi scaling factor. The difference in minimum radius between IPTG-facing and opposite wedges was calculated over time.

Analyses were restricted to ~3–22 h, the interval during which the inoculum initially became detectable, and the colony edge remained separable from the dish rim. For slope estimation only, radius-difference trajectories were smoothed using a moving mean (window size = 5). Linear fits (*polyfit*) were computed for pre-IPTG (3–6 h) and post-IPTG (18–21 h) windows, and the change in slope (post – pre) was calculated for each biological replicate. Statistical comparisons between strains were performed using a two-sample, two-tailed Welch’s *t*-test (*ttest2*, unequal variances) and a Wilcoxon rank-sum test (*ranksum*).

## Supplementary information

## Data Availability

The datasets and computer code produced in this study are available in the following databases: Endpoint swarm colony image datasets: Zenodo (https://doi.org/10.5281/zenodo.19612563). Time-lapse swarm colony image datasets: Zenodo (https://doi.org/10.5281/zenodo.19615229). Plate-reader data, processed datasets, model outputs, and analysis code: GitHub (https://github.com/daninolab/ecoli-swarming-recording) and Zenodo (https://doi.org/10.5281/zenodo.20629540). Strains and plasmids produced in this study are available from the corresponding author upon reasonable request. The source data of this paper are collected in the following database record: biostudies:S-SCDT-10_1038-S44320-026-00232-7.
