# Supplementary material for: Engineered E. coli swarming for binary and analog input recording
Source: Mol Syst Biol. 2026 Jul 16;22(9):1377–400. doi: 10.1038/s44320-026-00232-7 (PMC13538604; doi:10.1038/s44320-026-00232-7)
Supplement: Supplementary file 1 [file 44320_2026_232_MOESM13_ESM.pdf]

Expanded View Figures

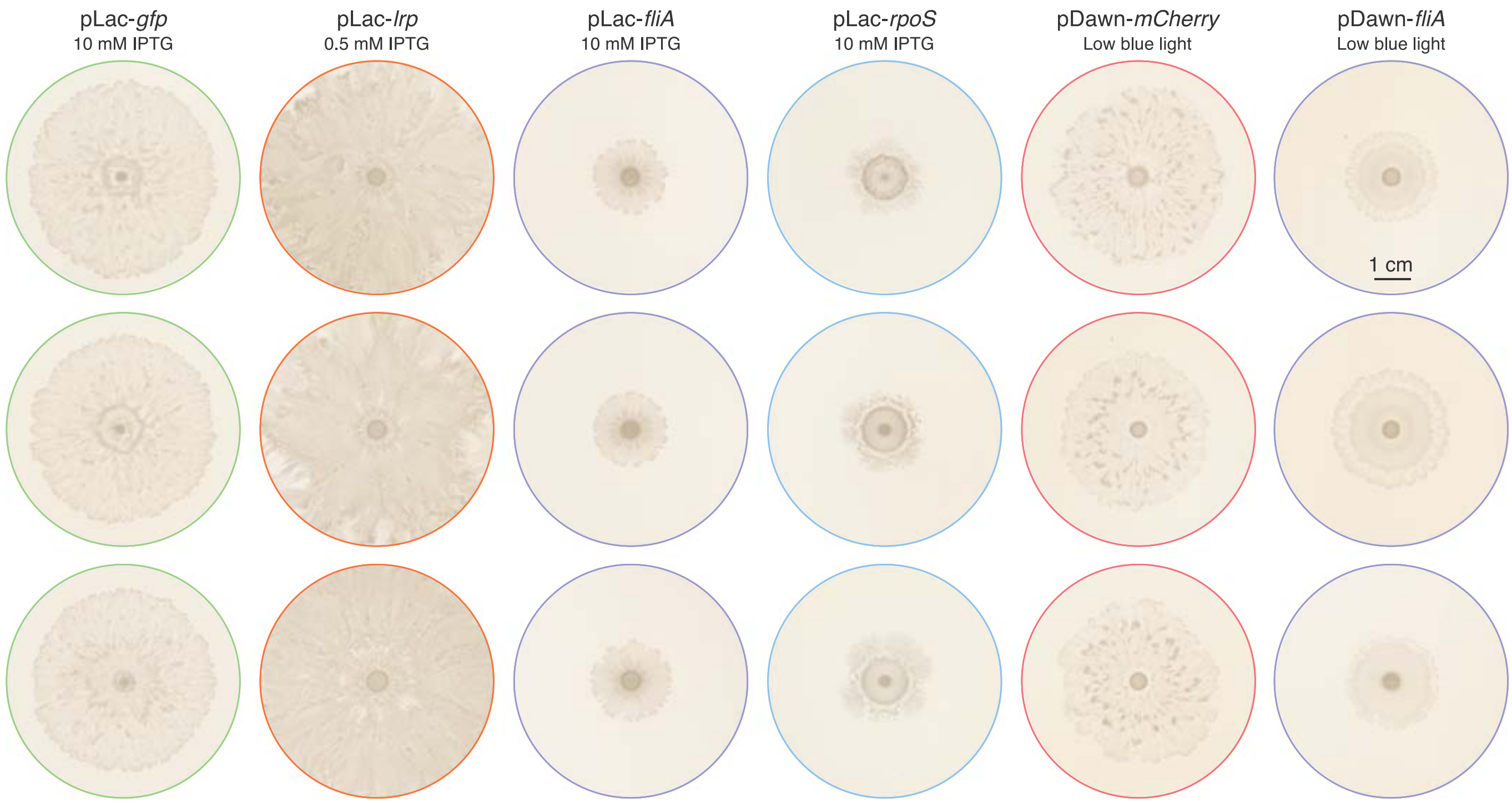

Figure EV1. Engineered swarm colonies exhibit reproducible morphologies across biological replicates and experimental days.

Representative replicate images of 24-h swarm colonies formed by engineered MG1655hm strains on swarming medium with select induction conditions (IPTG or blue light, as indicated). For each strain, a single induction condition was chosen from screening assays to illustrate characteristic morphologies. Across rows, three independent biological replicates are shown ($n = 3$), demonstrating reproducibility across experiments.

© The Author(s)

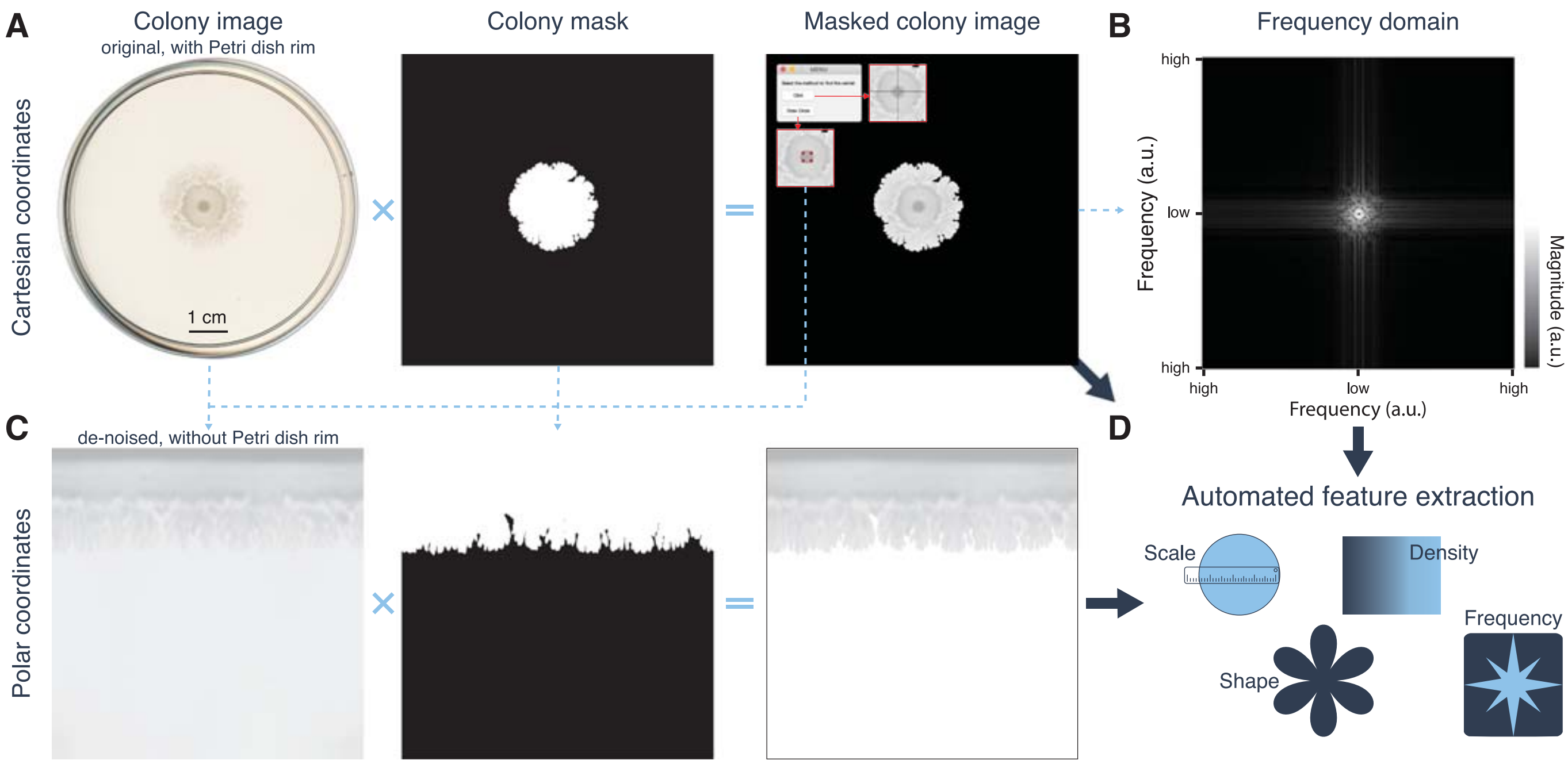

Figure EV2. Semi-automated computational pipeline for swarm pattern quantification.

(**A**) Swarm colonies in 100-mm Petri dishes were scanned at 400 dpi using a commercially available flatbed document scanner. Colony images were segmented with image-processing functions and minor manual refinement in Adobe Photoshop as needed. Masked colonies were transformed from the spatial to (**B**) frequency domain (via Fourier transform), and from Cartesian to (**C**) polar coordinates, with user input to locate the inoculum center. (**D**) From these three colony representations (Cartesian, Fourier, and polar), features of scale, density, shape, and frequency were extracted for downstream analysis. (Example shown: MG1655hm pLac-*rpoS* at 2.5 mM IPTG, 24 h).

 © The Author(s)

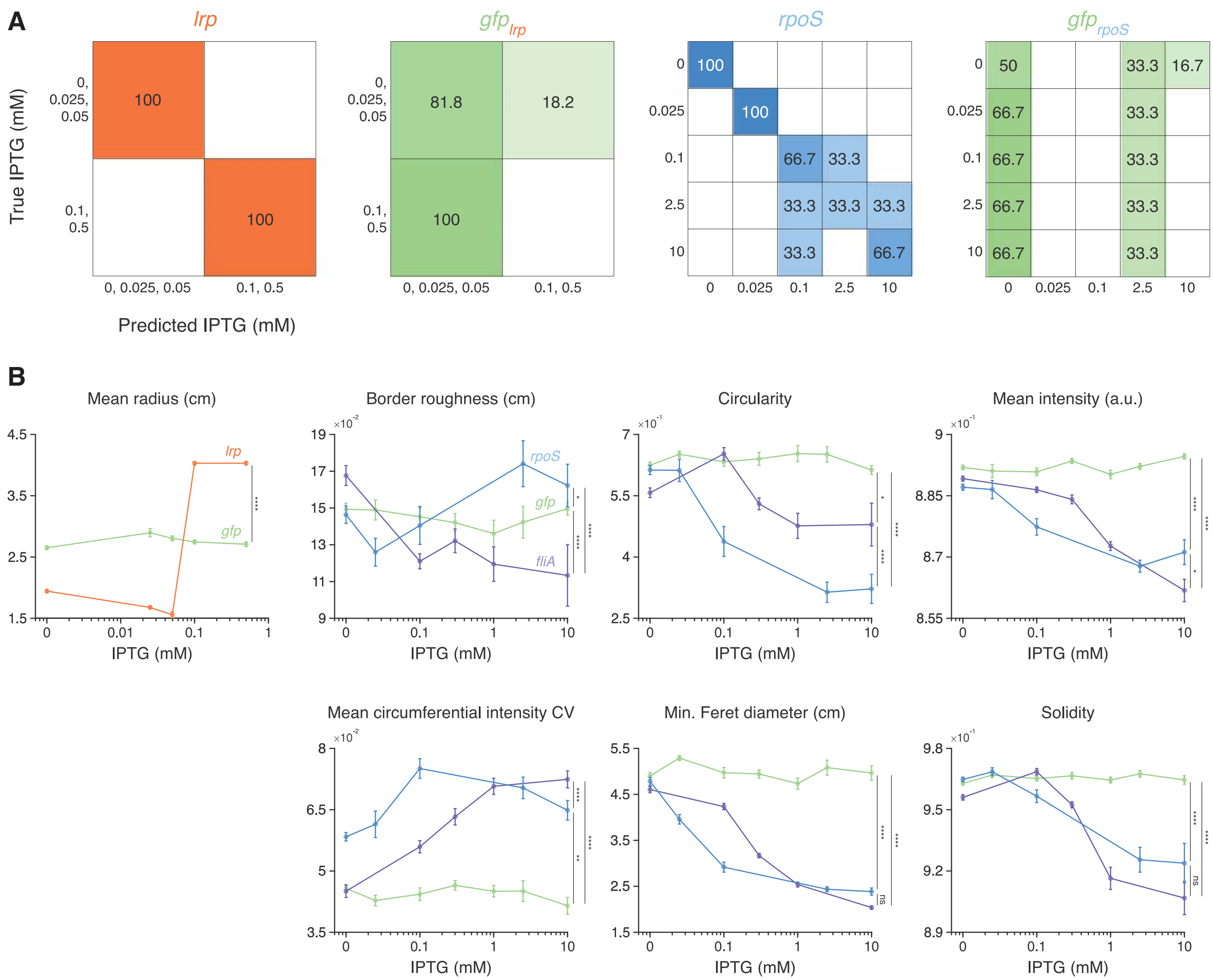

Figure EV3. Regression models decode IPTG from swarm pattern features.

(**A**) Test confusion matrices of regression models decoding IPTG concentration from 24-h colony features of pLac-*lrp*, pLac-*rpoS*, and their corresponding pLac-*gfp* control strains. Confusion matrices share the same axes; inner values indicate the percentage of test predictions within each true IPTG class. Confusion matrices for the pLac-*fliA* model and its corresponding pLac-*gfp* control model are shown in Fig. 4A. (**B**) Select IPTG-dependent trends of morphological and intensity-based features identified through strain-specific model fitting. The pLac-*lrp* model used mean radius; the pLac-*fliA* model used border roughness, circularity, and mean intensity; and the pLac-*rpoS* model used mean circumferential intensity CV, minimum Feret diameter, and solidity. Points represent the mean of independent biological replicates (swarm colonies): $n = 9$–18 for the pLac-*lrp/gfp* strain set, and $n = 9$–35 for the pLac-*fliA/rpoS/gfp* strain sets, collected across ≥3 independent experiments per condition performed on different days. Error bars represent SEM. Asterisks denote significant differences between the overall IPTG-response curves, determined using linear mixed-effects models with ANOVA for the pLac-*lrp/gfp* strain set and with post hoc comparisons for the pLac-*fliA/rpoS/gfp* strain sets (both based on the Strain × IPTG interaction; $P \leq 0.01$; see Methods, "Statistical analysis of colony features"). Exact *P* values are reported in Appendix Table S3.

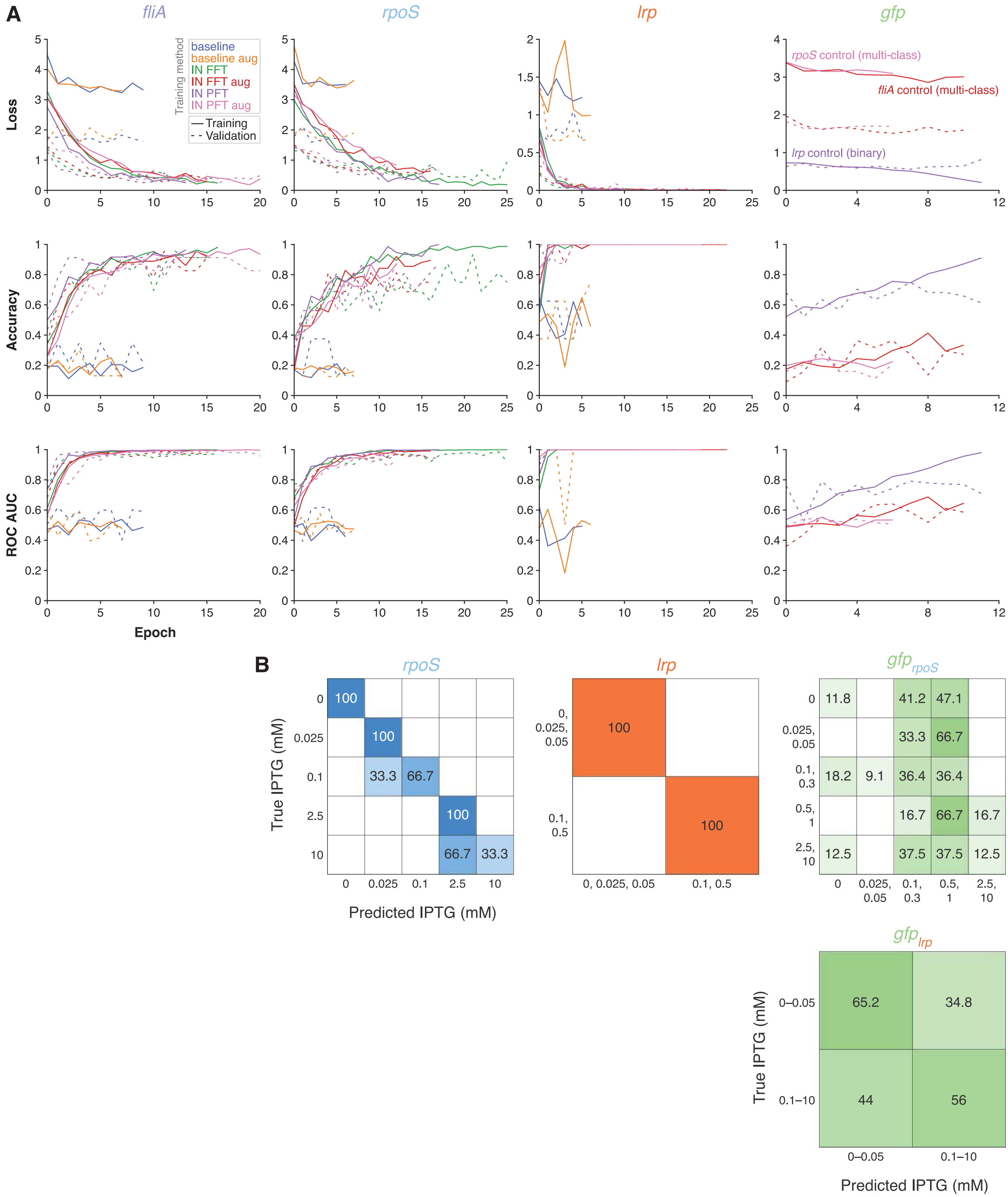
A
fliA
rpoS
lrp
gfp
Loss
Accuracy
ROC AUC
Epoch
Training method
baseline
baseline aug
IN FFT
IN FFT aug
IN PFT
IN PFT aug
— Training
- - Validation
rpoS control (multi-class)
fliA control (multi-class)
lrp control (binary)
B
rpoS
lrp
gfp rpoS
gfp lrp
True IPTG (mM)
Predicted IPTG (mM)
0
0.025
0.1
2.5
10
100
33.3
66.7
0, 0.025, 0.05
0.1, 0.5
0.025, 0.05
0.1, 0.3
0.5, 1
2.5, 10
11.8
41.2
47.1
18.2
9.1
36.4
16.7
12.5
37.5
0–0.05
0.1–10
65.2
34.8
44
56

 © The Author(s)

Figure EV4. Deep-learning models decode IPTG from swarm colony images.

(**A**) Training and validation learning curves (loss, accuracy, ROC–AUC) for SwinTransformer models trained on pLac-*fliA*, pLac-*rpoS*, pLac-*lrp*, and pLac-*gfp* image datasets. Model configurations included ImageNet pretraining (IN), full or partial fine-tuning (FFT, PFT), and random data augmentation (aug). Baseline models lacked ImageNet pretraining and fine-tuning. (**B**) Test confusion matrices of the optimal models for pLac-*rpoS* and pLac-*lrp*, defined as those achieving the lowest validation loss, and their corresponding pLac-*gfp* control models. The pLac-*gfp* models were trained and evaluated for multiclass IPTG classification (top; corresponding to the pLac-*rpoS* model configurations) and binary IPTG classification (bottom; corresponding to the pLac-*lrp* model configurations). Confusion matrices for the pLac-*fliA* model and its corresponding pLac-*gfp* control model are shown in Fig. 4C. Axes are shared across confusion matrices.

© The Author(s)

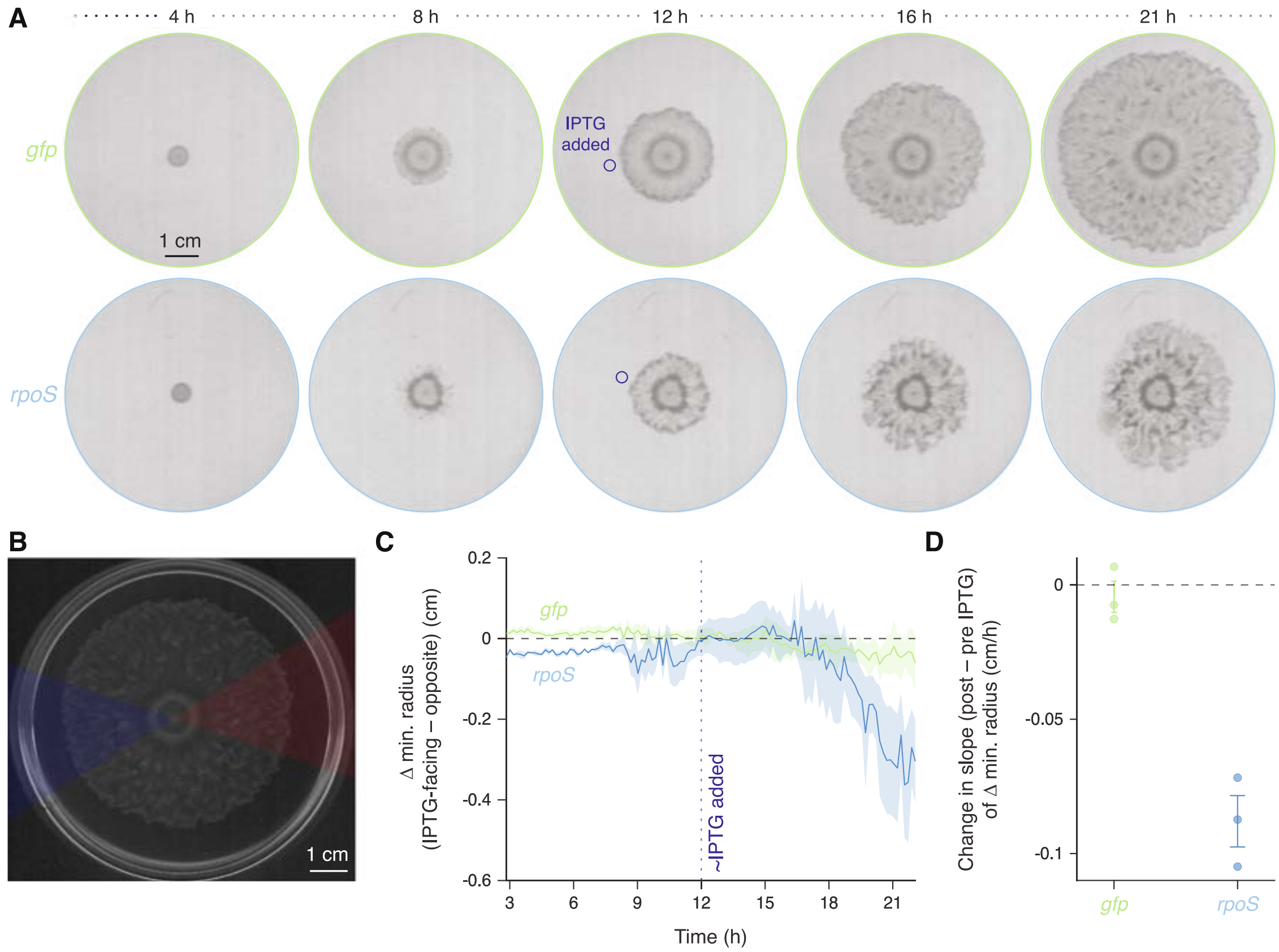

Figure EV5. Localized IPTG perturbation induces asymmetric swarm expansion.

(**A**) Four-hour snapshot images from representative time-lapse swarming assays. Shown are the control strain (pLac-*gfp*) and an analog strain (pLac-*rpoS*) on swarming medium. At ~12 h, 2 µL of 1 M IPTG was spotted just outside the expanding colony front. (**B**) Representative uncropped endpoint image of a pLac-*gfp* swarm colony (~22 h) with overlaid analysis masks. The blue wedge denotes the IPTG-facing region, and the red wedge denotes the opposite, untreated control region. (**C**) Time course of the difference in minimum colony radius between the IPTG-facing and opposite regions, with the timing of localized IPTG addition indicated. (**D**) Change in the slope of the minimum-radius difference post-IPTG relative to pre-IPTG. Two-sample, two-tailed Welch's *t*-test: $P = 3.5 \times 10^{-3}$; Wilcoxon rank-sum test: $P = 0.1$. In plots (**C**, **D**), lines represent the mean of $n = 3$ biological replicates; shaded regions and error bars represent SEM; points indicate individual biological replicates.

 © The Author(s)
