# Supplementary material for: Engineered E. coli swarming for binary and analog input recording
Source: Mol Syst Biol. 2026 Jul 16;22(9):1377–400. doi: 10.1038/s44320-026-00232-7 (PMC13538604; doi:10.1038/s44320-026-00232-7)
Supplement: Supplementary file 2 [file 44320_2026_232_MOESM1_ESM.pdf]

Appendix

Engineered *E. coli* swarming for binary and analog input recording

Table of Contents

Appendix Figures

Appendix Fig. S1 | Optimizing swarming conditions and characterizing pLac induction 3

Appendix Fig. S2 | MG1655hm pLac-*gfp* control swarm patterns 5

Appendix Fig. S3 | MG1655hm pLac-*cheZ* swarm patterns 6

Appendix Fig. S4 | Dual-strain deep-learning model for joint strain–IPTG decoding 7

Appendix Fig. S5 | Blue-light induction with the pDawn system 8

Appendix Tables

Appendix Table S1 | *E. coli* swarming-related and reporter genes used in inducible expression plasmids 9

Appendix Table S2 | Primers used for Gibson Assembly of IPTG-inducible pLac-[*swarming gene*] plasmids ... 10

Appendix Table S3 | Pairwise *P* values for overall IPTG–response curves of pLac strains (α = 0.01). 12

Appendix Table S4 | Image dataset sizes by pLac strain and IPTG for regression models 13

Appendix Table S5 | Final regression model parameters and performance metrics 14

Appendix Table S6 | Image dataset sizes by pLac strain and IPTG for SwinTransformer models 15

Appendix Table S7 | Test performance metrics for final SwinTransformer models 16

Appendix Table S8 | Pairwise *P* values for light-response features in pDawn strains (α = 0.01) 17

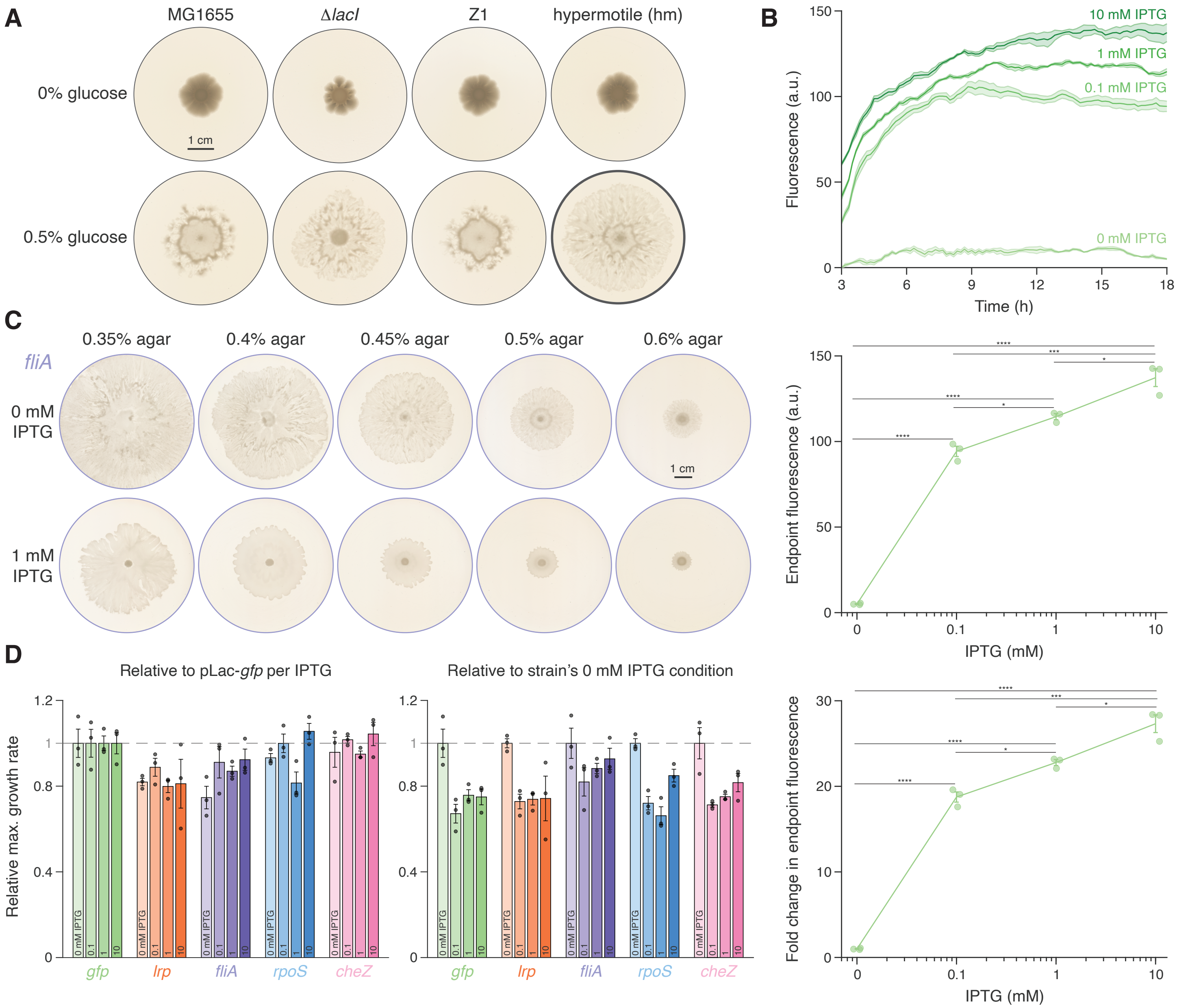

Appendix Figure S1 | Optimizing swarming conditions and characterizing pLac induction. A, Baseline swarming conditions were optimized by varying *E. coli* MG1655 strain backgrounds (parent, hypermotile "hm," Δ*lacI*, and Z1) and glucose supplementation in standard Petri dishes (100-mm diameter). Shown here, representative images of colonies after 24 hours of 37 °C incubation. The Δ*lacI* strain lacks the LacI repressor, while the Z1 strain carries a chromosomally integrated copy of *lacI* that drives high LacI expression. The finalized protocol used the hypermotile variant (MG1655hm) on 20 mL of swarming medium consisting of 0.45% Eiken agar (slightly less than the 0.5% shown), 0.5% glucose, and 2% Luria–Bertani (LB) broth. **B,** Fluorescence response of MG1655hm carrying the IPTG-inducible pLac-*gfp* plasmid across IPTG concentrations in a 96-well

kinetic plate-reader assay. Top: Fluorescence trajectories over time. Middle: Endpoint fluorescence (final time point, ~18 h). Bottom: Fold change in endpoint fluorescence relative to the 0 mM IPTG condition. Statistical comparisons were performed by one-way ANOVA with Tukey–Kramer post-hoc tests. Exact P values (from top to bottom on the middle and bottom plots) are: ****$P = 4.48 \times 10^{-9}$, ***$P = 4.74 \times 10^{-5}$, *$P = 3.79 \times 10^{-3}$, ****$P = 2.83 \times 10^{-8}$, *$P = 7.65 \times 10^{-3}$, ****$P = 1.65 \times 10^{-7}$. **C,** After verifying liquid-culture inducibility of pLac-*gfp* and cloning pLac-[*swarming gene*] plasmids, swarming conditions were further tuned by varying Eiken agar concentration with and without 1 mM IPTG. A concentration of 0.45% agar was chosen, as it preserved the uninduced MG1655hm swarm pattern while yielding the clearest IPTG-dependent differential in colony features. **D,** Relative maximum growth rates of MG1655hm pLac strains across IPTG concentrations. Growth rates were extracted from OD_{600} trajectories as described in Methods (*Liquid-culture induction analysis*) and normalized either to the mean growth rate of pLac-*gfp* at the same IPTG concentration (left) or the strain's own mean growth rate at 0 mM IPTG (right). In all plots (B**,** D), lines or bars represent the mean of n = 3 replicate wells; shaded regions and error bars represent the standard errors of the mean (SEM); points show individual replicate wells.

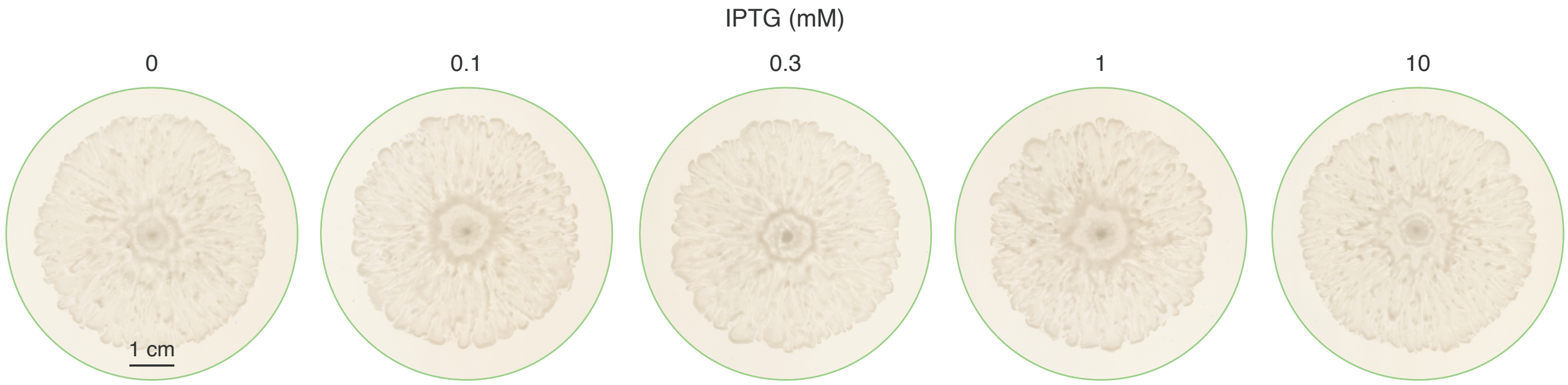

Appendix Figure S2 | MG1655hm pLac-*gfp* control swarm patterns. Representative images of 24-h colonies formed on swarming medium supplemented with the empirically selected IPTG concentrations used in the pLac-*fliA* experiments (Fig. 3A, top row). pLac-*gfp* control colonies were grown in parallel with the pLac-*fliA* experiments. *gfp* induction did not alter baseline swarm colony morphology across the tested IPTG range.

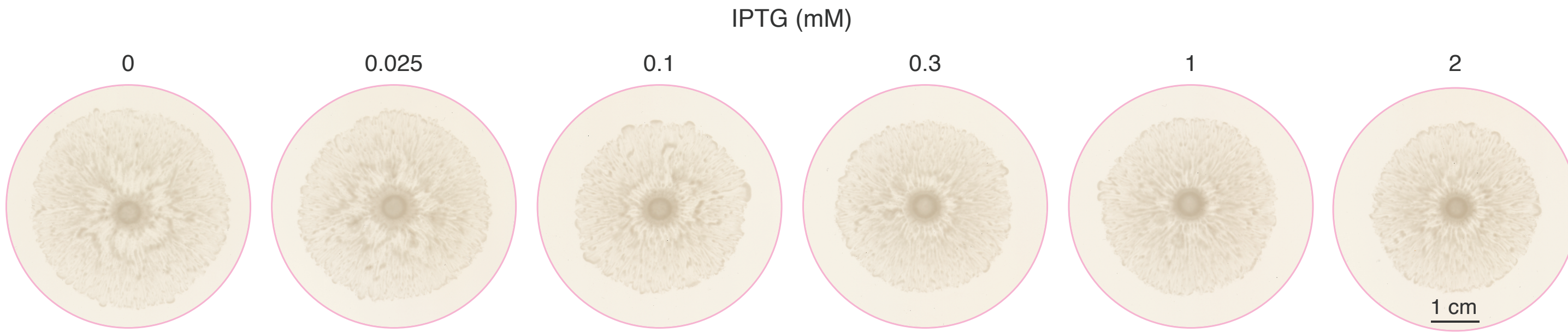

Appendix Figure S3 | MG1655hm pLac-*cheZ* swarm patterns. Representative images of 24-h colonies formed on swarming medium supplemented with a range of IPTG concentrations. *cheZ* overexpression did not notably alter baseline swarm colony morphology across the tested IPTG range.

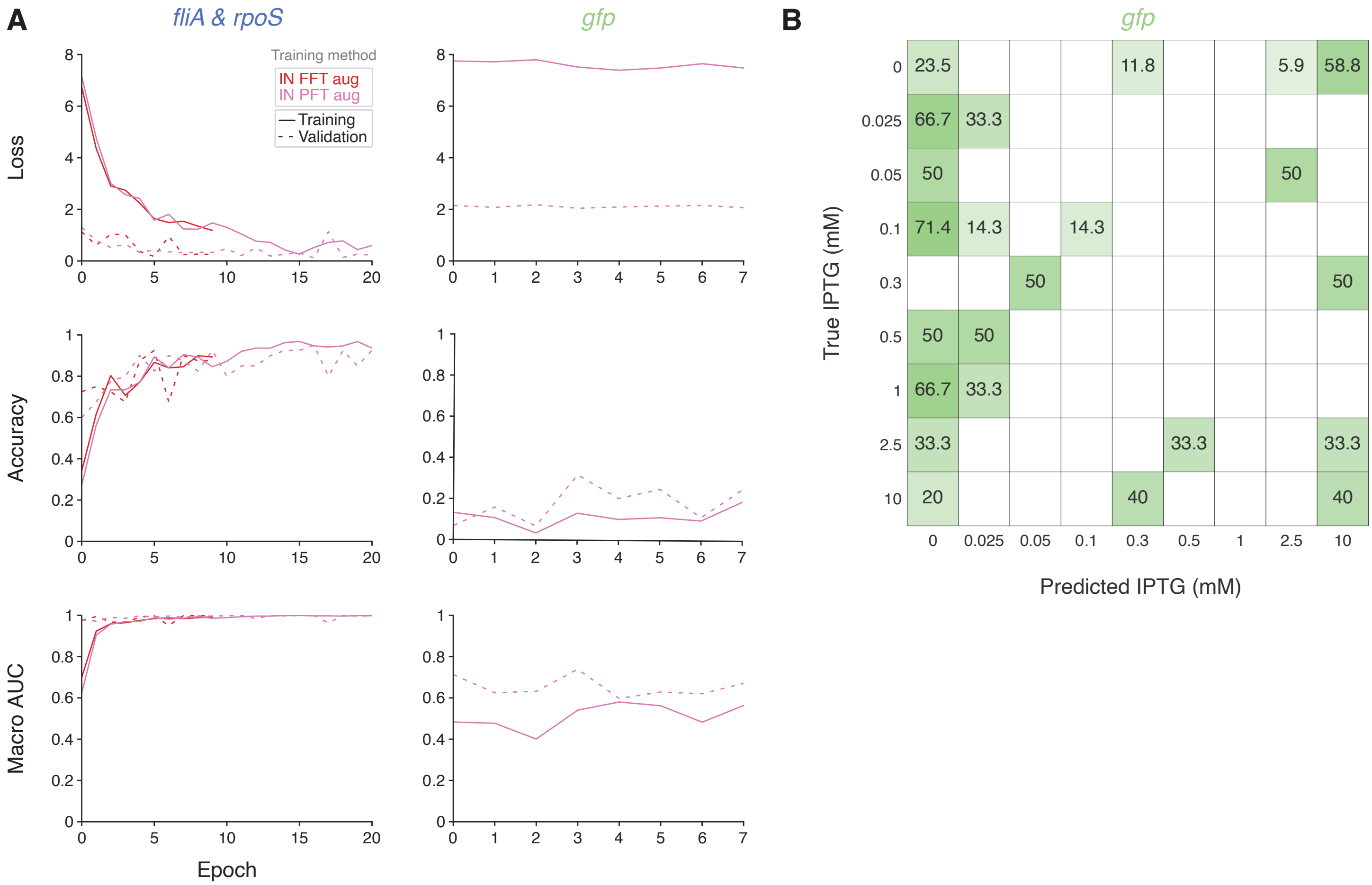

Appendix Figure S4 | Dual-strain deep-learning model for joint strain–IPTG decoding. A, Training and validation learning curves (loss, accuracy, macro-averaged ROC–AUC) for SwinTransformer models trained on nine-class pooled pLac-*fliA* and pLac-*rpoS* image datasets (left) and on the corresponding pLac-*gfp* control dataset (right). Model configurations included ImageNet pretraining (IN), full or partial fine-tuning (FFT, PFT), and random data augmentation (aug). **B,** Test confusion matrix of the nine-class pLac-*gfp* control model. Inner values indicate the percentage of test predictions within each true class. The confusion matrix for the joint pLac-*fliA*/*rpoS* model is shown in Fig. 4D.

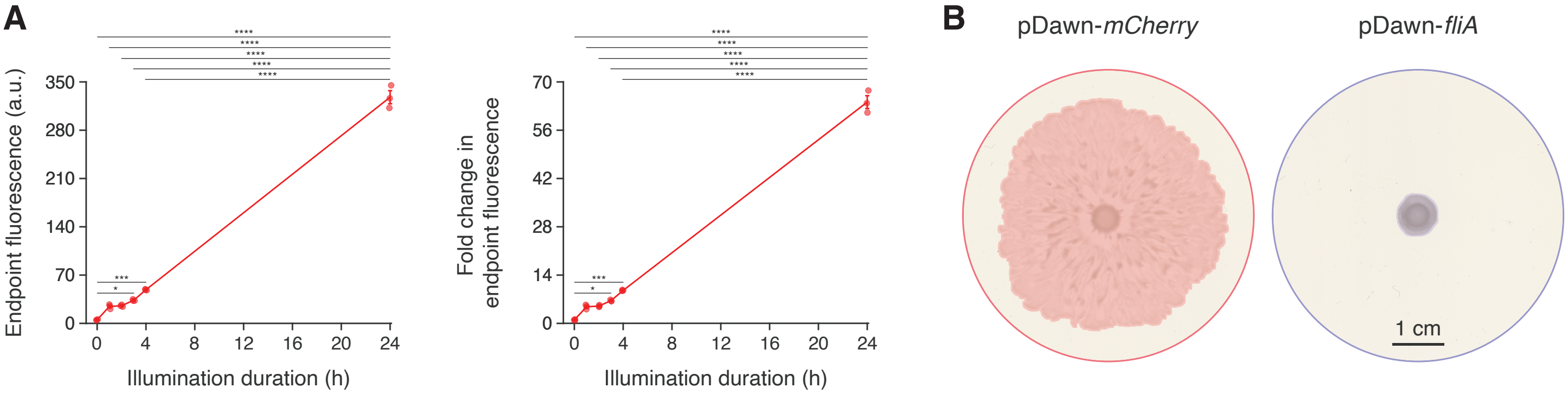

Appendix Figure S5 | Blue-light induction with the pDawn system. A, Blue-light induction of MG1655hm pDawn-*mCherry* in liquid culture. Left: endpoint fluorescence was measured after a fixed total culture time of 24 h following transient blue-light illumination applied for the indicated durations. Right: fold change is reported relative to the non-induced condition (0 h illumination). Blue-light irradiance corresponded to the high–blue-light condition defined in Fig. 6B. Points represent individual replicate wells (n = 3); lines connect the means; error bars represent SEM. Statistical comparisons were performed by one-way ANOVA with Tukey–Kramer post-hoc tests. ****$P = 2.24 \times 10^{-8}$ for all pairwise comparisons between 24-h illumination and shorter illumination durations; ****$P = 5.97 \times 10^{-5}$ for 0 vs. 4 h; *$P = 2.98 \times 10^{-3}$ for 0 vs. 3 h; all other pairwise differences were not significant (ns). **B,** Representative images of 24-h high–blue-light-induced colonies, shown raw in **Fig. 6B**, overlaid with false-color binary colony segmentation masks used for feature extraction.

Appendix Table S1 | *E. coli* swarming-related and reporter genes used in inducible expression plasmids.

Gene	Product	Sequence (5' to 3')
lrp	Leucine-responsive transcriptional regulatory protein	atggtagatagcaagaagcgccctggcaaagatctcgaccgtatcgatcgtaacattctt<br>aatgagttgcaaaaggatgggcgtatttctaacgtcgagctttctaaacgtgtgggactttc<br>cccaacgccgtgccttgagcgtgtgcgtcggctggaaagacaagggtttattcagggcta<br>tacggcgctgcttaacccccattatctggatgcatcacttctggtattcgttgagattactctg<br>aatcgtggcgcaccggatgtgtttgaacaattcaataccgctgtacaaaaacttgaagaa<br>attcaggagtgtcatttagtatccggtgatttcgactacctgttgaaaacacgcgtgccgga<br>tatgtcagcctaccgtaagttgctggggaaaccctgctgcgtctgcctggcgtcaatgac<br>acacggacatacgttgttatggaagaagtcaagcagagtaatcgtctggttattaagacg<br>cgctaa
fliA	Class II, flagellum-specific RNA polymerase sigma factor, σ^{28}	ttataacttacccagtttagtgcgtaaccgtttaatagcctggctgtgtaactgactgacccg<br>cgattccccgacctccagcaccgcgccaatctctttgagattcagctcttcctggtaataga<br>gggttaataccagtttttcgcgctccggcaacgtttcgatggcttccatcacccgctggcgc<br>agattactgtccagtagttgttgtagcgggttttctcgctgatgatcatcagtaaccagttcga<br>tgctatcgccgtgctcttcgcgccactcatcgtaggagaagagctggctgttattggtgtcg<br>agcaacatttggcgataatcggcaatatcgatccctaaacgttccgctacctcagtttccgt<br>ggcgttgcggccaagttcctgctccagttgccctattgcctgtgccacttcacgcgcgttgc<br>gtcgcacgctgcgcggcacccagtcacggctgcgaagttcatccagcatagcgccacg<br>gatacgctgcactgcgtaagttgtaaatgccgttccttgtagggcgtcatagcgttcgacgg<br>cattaagtaacccaatgccgcccgcctgtagcagatcgtcaagttccacgctcgcgggc<br>agtcgaacctgcaggcgcaatgcttcgtgacgcaccagcgggacataacgctgccaca<br>gcgagtgtttatccattacaccttcagcggtatagagtgaattcac
rpoS	Global stress regulator; RNA polymerase sigma factor, σ^{S}	ttactcgcggaacagcgcttcgatattcagcccctgcgtttgcaggatttcgcgcaaacgg<br>cgcaggccttcaacctgaatctggcgaacacgttcacgggtgaggccaatttcacgacct<br>acatcttccagtgttgccgcttcgtaccccagcaaaccgaatcgacgtgccagcacttca<br>cgctgtttggcgttcagctcgaacagccatttgacgatgctctgcttcatatcgtcatcttgcg<br>tggtatcttccggaccgttctctttttcatcggccaggatgtccagcaacgctttttcggaatc<br>accacccagcggggtgtctaccgaggtaatgcgctcgttaagacgaagcatacggctg<br>acgtcatcaactggcttatccagttgctctgcgatctcttccgcacttggttcatggtccagctt<br>atgggacaactcacgtgcggttcgcaggtaaacgttcagctccttttacgatgtgaatcggc<br>aaacgaatagtacgggtttggttcataatcgcccgttcaatcgtctggcgaatccaccagg<br>ttgcgtatgttgagaagcggaaaccacgttccgggtcaaacttctctaccgcgcggatca<br>gccccaggttgccctcttcgataaggtccagcaacgccagaccacgattgccataacgg<br>cgggcaatttttaccaccagacgcaagttactctcgatcatccggcggcgagaggcgac<br>atctccacgcagtgcgcgacgcgcaaaataaacttcttcttcggccgttaacagtggtga<br>ataaccaatctcaccaaggtaaagctgagtcgcgtccaacacacgctgtgtggctccctg<br>cgataacagttcctcttcggccaaatcgttatcactgggttcctgttctactaaggccttttcgt<br>caaaaacctcaactccgttctcatcaaattccgcatcttcatttaaatcatgaactttcagcgt<br>attctgactcat
cheZ	Phosphatase in chemosensory network	Tcaaaatccaagactatccaacaaatcgtccacctgatcctgactggctaccacaccgg<br>ctttgctggtatcgacctgaggtccattaagcaaactctggttttcacgttttggacgcgactc<br>ctgttccgggatgttttccaacagcaccatcagcaactggcgttcgatctcctgaatgacat<br>ccatcatccgcttaatgacctgcccggtgagatcctgaaaatcctgcgccatcatgatttcc<br>agcagttgcgcgttagtaaagctggtatgcgcgggtacatctgccagaaattgtcgtgtat<br>ctgttaccagttcacgggcgtcggcaaggtcaatcggatcggcaaaccagtcatcccaa<br>cgttgggttaacgcttttgctgatttctccatttgatcctgatgcggttgtgacgcctcaacact<br>gttcagcgcccgctccgcagcctgggcggtcatctgcacaacatagtacaaacgatcgc<br>gcgcatcggggatggcttccgccgcttcggcaatggcctgatccagccccagttcccgc<br>aaactgtcgcgcagcatacgcgtcaggctgccgatgcgcgcaatgatatcgccagctg<br>aatgctcgtcagcaggtttgattgatggttgcatcat

gfp	Green fluorescent protein	atgtctaaaggtgaagaattattcactggtgttgtcccaattttggttgaattagatggtgatgt taatggtcacaaattttctgtctccggtgaaggtgaaggtgatgctacttacggtaaattgac cttaaatttatttgtactactggtaaattgccagttccatggccaaccttagtcactactttaa cttatggtgttcaatgtttttctagatacccagatcatatgaaacaacatgactttttcaagtct gccatgccagaaggttatgttcaagaaagaactatttttttcaaagatgacggtaactaca agaccagagctgaagtcaagtttgaaggtgataccttagttaatagaatcgaattaaaag gtattgattttaaagaagatggtaacattttaggtcacaaattggaatacaactataactctc acaatgtttacatcatggctgacaaacaaaagaatggtatcaaagttaacttcaaaattag acacaacattgaagatggttctgttcaattagctgaccattatcaacaaaatactccaattg gtgatggtccagtcttgttaccagacaaccattacttatccactcaatctaaattatccaaag atccaaacgaaaagagagaccacatggtcttgttagaatttgttactgctgctggtattacc catggtatggatgaattgtacaaa
mCherry	mCherry fluorescent protein	atggtgagcaagggcgaggaggataacatggccatcatcaaggagttcatgcgcttca aggtgcacatggagggctccgtgaacggccacgagttcgagatcgagggcgagggc gagggccgcccctacgagggcacccagaccgccaagctgaaggtgaccaagggtg gcccccctgcccttcgcctgggacatcctgtcccctcagttcatgtacggctccaaggccta cgtgaagcaccccgccgacatccccgactacttgaagctgtccttccccgagggcttcaa gtgggagcgcgtgatgaacttcgaggacggcggcgtggtgaccgtgacccaggactcc tccttgcaggacggcgagttcatctacaaggtgaagctgcgcggcaccaacttcccctcc gacggccccgtaatgcagaagaagaccatgggctgggaggcctcctccgagcggatg taccccgaggacggcgccctgaagggcgagatcaagcagaggctgaagctgaagga cggcggccactacgacgctgaggtcaagaccacctacaaggccaagaagcccgtgc agctgcccggcgcctacaacgtcaacatcaagttggacatcacctcccacaacgagga ctacaccatcgtggaacagtacgaacgcgccgagggccgccactccaccggcggcat ggacgagctgtacaagtaa

Appendix Table S2 | Primers used for Gibson Assembly of IPTG-inducible pLac-*[swarming gene*] plasmids[1].

Name	Description	Sequence (5' to 3')
*lrp*_f_OH	Forward (f) primer for *lrp*	CACACAGAATTCATTAAAGAGGAGAAAGGTACCatggtagatagcaagaagcg
*lrp*_r_OH	Reverse (r) primer for *lrp*	CGTTTTATTTGATGCCTCTAGCACGCGTttagcgcgtcttaataaccag
*fliA*_f_OH	Forward primer for *fliA*	CACACAGAATTCATTAAAGAGGAGAAAGGTACCgtgaattcactctataccgc
fliA_r_OH	Reverse primer for *fliA*	CGTTTTATTTGATGCCTCTAGCACGCGTttataacttacccagtttagtgc
*rpoS*_f_OH	Forward primer for *rpoS*	CACACAGAATTCATTAAAGAGGAGAAAGGTACCatgagtcagaatacgc
*rpoS*_r_OH	Reverse primer for *rpoS*	CGTTTTATTTGATGCCTCTAGCACGCGTttactcgcggaacag
*cheZ*_f_OH	Forward primer for *cheZ*	CACACAGAATTCATTAAAGAGGAGAAAGGTACCatgatgcaaccatcaatcaaacctgc
*cheZ*_r_OH	Reverse primer for *cheZ*	CGTTTTATTTGATGCCTCTAGCACGCGTtcaaaatccaagactatccaacaaatcgtcc
lac_bb_f	Forward primer for pLac-*gfp* backbone (bb)	acgcgtgctagaggcatcaaataaaacg
lac_bb_r	Reverse primer for pLac-*gfp* backbone	ggtacctttctcctctttaatgaattctgtgtg

[1]Capitalized bases in primer sequences represent overhangs (OH).

Appendix Table S3 | Pairwise *P* values for overall IPTG–response curves of pLac strains (α = 0.01).

Figure	pLac strain comparison	Feature	*P* value	Significance
Fig. 2C	*lrp* vs. *gfp*	Colony area	1.33E-12	****
Fig. 2E	*lrp* vs. *gfp*	Mean circumferential CV	1.09E-08	****
Fig. 2E	*lrp* vs. *gfp*	Total low-frequency magnitudes	4.17E-04	**
Fig. 3C	*fliA* vs. *gfp*	Mean radius	2.95E-31	****
Fig. 3C	*rpoS* vs. *gfp*	Mean radius	1.70E-27	****
Fig. 3C	*fliA* vs. *rpoS*	Mean radius	8.66E-02	ns
Fig. 3D	*fliA* vs. *gfp*	Low–intermediate frequency weight	3.97E-10	****
Fig. 3D	*rpoS* vs. *gfp*	Low–intermediate frequency weight	3.11E-01	ns
Fig. 3D	*fliA* vs. *rpoS*	Low–intermediate frequency weight	1.56E-10	****
Fig. 3E	*fliA* vs. *gfp*	Eccentricity	3.78E-02	ns
Fig. 3E	*rpoS* vs. *gfp*	Eccentricity	8.09E-04	**
Fig. 3E	*fliA* vs. *rpoS*	Eccentricity	3.99E-06	****
Fig. 3F	*fliA* vs. *gfp*	Maximum intensity	1.64E-06	****
Fig. 3F	*rpoS* vs. *gfp*	Maximum intensity	6.39E-01	ns
Fig. 3F	*fliA* vs. *rpoS*	Maximum intensity	8.12E-05	***
Fig. 5B	*fliA* vs. *gfp*	Colony expansion rate	1.18E-13	****
Fig. 5B	*rpoS* vs. *gfp*	Colony expansion rate	2.39E-04	**
Fig. 5B	*fliA* vs. *rpoS*	Colony expansion rate	3.31E-06	****
Fig. 5B	*fliA* vs. *gfp*	Local textural uniformity	4.34E-14	****
Fig. 5B	*rpoS* vs. *gfp*	Local textural uniformity	7.47E-06	***
Fig. 5B	*fliA* vs. *rpoS*	Local textural uniformity	3.91E-05	****
Fig. EV3B	*lrp* vs. *gfp*	Mean radius	6.34E-12	****
Fig. EV3B	*fliA* vs. *gfp*	Border roughness	7.34E-06	****
Fig. EV3B	*rpoS* vs. *gfp*	Border roughness	2.24E-03	*
Fig. EV3B	*fliA* vs. *rpoS*	Border roughness	1.64E-10	****
Fig. EV3B	*fliA* vs. *gfp*	Circularity	3.36E-03	*
Fig. EV3B	*rpoS* vs. *gfp*	Circularity	8.49E-21	****
Fig. EV3B	*fliA* vs. *rpoS*	Circularity	3.82E-09	****
Fig. EV3B	*fliA* vs. *gfp*	Mean intensity	2.82E-29	****
Fig. EV3B	*rpoS* vs. *gfp*	Mean intensity	1.77E-20	****
Fig. EV3B	*fliA* vs. *rpoS*	Mean intensity	2.30E-03	*
Fig. EV3B	*fliA* vs. *gfp*	Mean circumferential CV	1.98E-20	****
Fig. EV3B	*rpoS* vs. *gfp*	Mean circumferential CV	3.19E-04	**
Fig. EV3B	*fliA* vs. *rpoS*	Mean circumferential CV	3.93E-09	****
Fig. EV3B	*fliA* vs. *gfp*	Minimum Feret diameter	1.46E-30	****
Fig. EV3B	*rpoS* vs. *gfp*	Minimum Feret diameter	1.71E-27	****
Fig. EV3B	*fliA* vs. *rpoS*	Minimum Feret diameter	1.29E-01	ns
Fig. EV3B	*fliA* vs. *gfp*	Solidity	2.82E-18	****
Fig. EV3B	*rpoS* vs. *gfp*	Solidity	7.75E-17	****
Fig. EV3B	*fliA* vs. *rpoS*	Solidity	3.62E-01	ns

Appendix Table S4 | Image dataset sizes by pLac strain and IPTG for regression models[2].

	Binomial		Multinomial			
**IPTG (mM)**	***lrp***	***gfp* (*lrp*)**	***fliA***	***gfp* (*fliA*)**	***rpoS***	***gfp* (*rpoS*)**
0	18	18	18	17	18	18
0.025	9	9	n/a	n/a	9	9
0.05	9	9	n/a	n/a	n/a	n/a
0.1	9	9	9	9	9	9
0.3	n/a	n/a	9	9	n/a	n/a
0.5	9	9	n/a	n/a	n/a	n/a
1	n/a	n/a	9	9	n/a	n/a
2.5	n/a	n/a	n/a	n/a	9	9
10	n/a	n/a	9	9	9	9

[2]Values indicate the number of independent biological replicates (colony images) from which features were extracted.

Appendix Table S5 | Final regression model parameters and performance metrics.

	Binomial		Multinomial			
	lrp	*gfp*	*fliA*	*gfp*	*rpoS*	*gfp*
Feature Name #1	Mean radius		Border roughness		Mean circ. intensity CV	
Estimated Coefficient #1	1.81	1.52	871.20	39.46	246.84	127.42
P value #1	3.59E-05	3.99E-01	8.72E-04	8.24E-01	3.81E-03	1.15E-02
Feature Name #2	n/a	n/a	Circularity		Min. feret diameter	
Estimated Coefficient #2	n/a	n/a	31.17	-1.03	12.83	2.34
P value #2	n/a	n/a	4.68E-04	8.78E-01	1.83E-03	1.29E-03
Feature Name #3	n/a	n/a	Mean intensity		Solidity	
Estimated Coefficient #3	n/a	n/a	198.84	-86.34	-119.77	35.96
P value #3	n/a	n/a	2.48E-03	1.54E-01	7.51E-03	3.47E-01
pseudo R^2	1.00	0.02	0.69	0.02	0.80	0.14
Train ROC–AUC	1.00	0.50	0.96	0.60	0.98	0.72
Test ROC–AUC	1.00	0.41	1.00	0.67	0.96	0.46

Appendix Table S6 | Image dataset sizes by pLac strain and IPTG for SwinTransformer models[3].

IPTG (mM)	*fliA*	*rpoS*	*lrp*	*gfp*
0	57	41	18	111
0.025	n/a	18	9	24
0.05	n/a	n/a	9	9
0.1	18	15	9	58
0.3	24	n/a	n/a	18
0.5	n/a	n/a	9	16
1	35	n/a	n/a	20
2.5	n/a	20	n/a	15
10	21	15	n/a	32

[3]Values indicate the number of independent biological replicates (colony images) included per strain and IPTG condition.

Appendix Table S7 | Test performance metrics for final SwinTransformer models[4].

		Five-class				Nine-class		Binary	
		fliA	*gfp*	*rpos*	*gfp*	*fliA/rpoS*	*gfp*	*lrp*	*gfp*
	**Training method**	IN FFT aug		IN PFT aug		IN PFT aug		IN PFT	
**Test metric**	**Accuracy**	0.9167	0.3333	0.8235	0.2292	0.9268	0.1818	1.000	0.6042
	**Top-2 Accuracy**	0.9583	0.5208	0.8824	0.4583	0.9756	0.4091	n/a	n/a
	**ROC–AUC**	0.9941	0.6324	0.8949	0.5836	0.9986	0.7009	1.000	0.5722

[4]Training method abbreviations: IN = ImageNet initialization; FFT = full fine-tuning; PFT = partial fine-tuning; aug = data augmentation.

Appendix Table S8 | Pairwise *P* values for light-response features in pDawn strains (α = 0.01)[5].

Feature	Group 1	Group 2	*P* value	Significance
Minimum radius	pDawn-*fliA* (dark)	pDawn-*fliA* (red)	7.42E-02	ns
Minimum radius	pDawn-*fliA* (dark)	pDawn-*fliA* (low blue)	5.44E-09	****
Minimum radius	pDawn-*fliA* (dark)	pDawn-*fliA* (high blue)	4.14E-21	****
Minimum radius	pDawn-*fliA* (red)	pDawn-*fliA* (low blue)	1.75E-03	**
Minimum radius	pDawn-*fliA* (red)	pDawn-*fliA* (high blue)	1.32E-14	****
Minimum radius	pDawn-*fliA* (low blue)	pDawn-*fliA* (high blue)	4.35E-06	****
Minimum radius	pDawn-*mCherry* (dark)	pDawn-*mCherry* (red)	4.39E-01	ns
Minimum radius	pDawn-*mCherry* (dark)	pDawn-*mCherry* (low blue)	1.69E-01	ns
Minimum radius	pDawn-*mCherry* (dark)	pDawn-*mCherry* (high blue)	2.87E-02	ns
Minimum radius	pDawn-*mCherry* (red)	pDawn-*mCherry* (low blue)	9.99E-01	ns
Minimum radius	pDawn-*mCherry* (red)	pDawn-*mCherry* (high blue)	9.20E-01	ns
Minimum radius	pDawn-*mCherry* (low blue)	pDawn-*mCherry* (high blue)	9.98E-01	ns
Minimum radius	pDawn-*fliA* (dark)	pDawn-*mCherry* (dark)	9.80E-01	ns
Minimum radius	pDawn-*fliA* (red)	pDawn-*mCherry* (red)	1.00E+00	ns
Minimum radius	pDawn-*fliA* (low blue)	pDawn-*mCherry* (low blue)	1.15E-02	ns
Minimum radius	pDawn-*fliA* (high blue)	pDawn-*mCherry* (high blue)	1.57E-12	****
Global intensity CV	pDawn-*fliA* (dark)	pDawn-*fliA* (red)	9.16E-01	ns
Global intensity CV	pDawn-*fliA* (dark)	pDawn-*fliA* (low blue)	8.52E-09	****
Global intensity CV	pDawn-*fliA* (dark)	pDawn-*fliA* (high blue)	0	****
Global intensity CV	pDawn-*fliA* (red)	pDawn-*fliA* (low blue)	3.58E-06	****
Global intensity CV	pDawn-*fliA* (red)	pDawn-*fliA* (high blue)	0	****
Global intensity CV	pDawn-*fliA* (low blue)	pDawn-*fliA* (high blue)	0	****
Global intensity CV	pDawn-*mCherry* (dark)	pDawn-*mCherry* (red)	9.44E-01	ns
Global intensity CV	pDawn-*mCherry* (dark)	pDawn-*mCherry* (low blue)	9.99E-01	ns
Global intensity CV	pDawn-*mCherry* (dark)	pDawn-*mCherry* (high blue)	9.47E-01	ns
Global intensity CV	pDawn-*mCherry* (red)	pDawn-*mCherry* (low blue)	9.99E-01	ns
Global intensity CV	pDawn-*mCherry* (red)	pDawn-*mCherry* (high blue)	3.16E-01	ns
Global intensity CV	pDawn-*mCherry* (low blue)	pDawn-*mCherry* (high blue)	7.35E-01	ns
Global intensity CV	pDawn-*fliA* (dark)	pDawn-*mCherry* (dark)	2.65E-03	**
Global intensity CV	pDawn-*fliA* (red)	pDawn-*mCherry* (red)	5.41E-03	**
Global intensity CV	pDawn-*fliA* (low blue)	pDawn-*mCherry* (low blue)	1.75E-01	ns
Global intensity CV	pDawn-*fliA* (high blue)	pDawn-*mCherry* (high blue)	0	****
Mean circumferential CV	pDawn-*fliA* (dark)	pDawn-*fliA* (red)	6.31E-01	ns
Mean circumferential CV	pDawn-*fliA* (dark)	pDawn-*fliA* (low blue)	8.45E-09	****
Mean circumferential CV	pDawn-*fliA* (dark)	pDawn-*fliA* (high blue)	8.05E-28	****
Mean circumferential CV	pDawn-*fliA* (red)	pDawn-*fliA* (low blue)	3.21E-05	****
Mean circumferential CV	pDawn-*fliA* (red)	pDawn-*fliA* (high blue)	1.29E-23	****
Mean circumferential CV	pDawn-*fliA* (low blue)	pDawn-*fliA* (high blue)	5.64E-13	****
Mean circumferential CV	pDawn-*mCherry* (dark)	pDawn-*mCherry* (red)	2.21E-01	ns
Mean circumferential CV	pDawn-*mCherry* (dark)	pDawn-*mCherry* (low blue)	8.93E-01	ns
Mean circumferential CV	pDawn-*mCherry* (dark)	pDawn-*mCherry* (high blue)	9.99E-01	ns

Mean circumferential CV	pDawn-*mCherry* (red)	pDawn-*mCherry* (low blue)	9.53E-01	ns
Mean circumferential CV	pDawn-*mCherry* (red)	pDawn-*mCherry* (high blue)	2.68E-01	ns
Mean circumferential CV	pDawn-*mCherry* (low blue)	pDawn-*mCherry* (high blue)	9.27E-01	ns
Mean circumferential CV	pDawn-*fliA* (dark)	pDawn-*mCherry* (dark)	9.99E-01	ns
Mean circumferential CV	pDawn-*fliA* (red)	pDawn-*mCherry* (red)	9.27E-01	ns
Mean circumferential CV	pDawn-*fliA* (low blue)	pDawn-*mCherry* (low blue)	4.74E-05	****
Mean circumferential CV	pDawn-*fliA* (high blue)	pDawn-*mCherry* (high blue)	1.92E-26	****

[5]*P* values reported as 0 indicate values below the numerical precision of the statistical computation.
