# Supplementary material for: Engineered E. coli swarming for binary and analog input recording
Source: Mol Syst Biol. 2026 Jul 16;22(9):1377–400. doi: 10.1038/s44320-026-00232-7 (PMC13538604; doi:10.1038/s44320-026-00232-7)
Supplement: Supplementary file 3 [file 44320_2026_232_MOESM2_ESM.pdf]

Engineered E. coli swarming for binary and analog input recording

Marian Shaw, Sadhya Garg, Julia Kirby, Samuel Schmidgall, Filippo Liguori, Anjali Doshi, Tao Tu, and Tal Danino

Corresponding author(s): Tal Danino (tal.danino@columbia.edu)

Review Timeline:

Submission Date:	7th Oct 25
Editorial Decision:	21st Nov 25
Revision Received:	21st Apr 26
Editorial Decision:	28th May 26
Revision Received:	10th Jun 26
Accepted:	25th Jun 26

Editor: Poonam Bheda

Transaction Report:

(Note: With the exception of the correction of typographical or spelling errors that could be a source of ambiguity, letters and reports are not edited. Depending on transfer agreements, referee reports obtained elsewhere may or may not be included in this compilation. Referee reports are anonymous unless the Referee chooses to sign their reports.)

21st Nov 2025

Manuscript Number: MSB-2025-13399-T
Title: Engineered E. coli swarming for binary and analog input recording

Dear Dr. Danino,

Thank you for the submission of your manuscript to Molecular Systems Biology. We have now received feedback from the three reviewers who agreed to evaluate your manuscript. As you will see from the reports below, the referees acknowledge the interest of the study and are overall supportive of your work; however they also comment on multiple aspects of the manuscript that should be strengthened in a revision.

I think that the recommendations of the reviewers are rather clear and I therefore do not see the need to repeat the comments listed below. All issues raised would need to be satisfactorily addressed. Please let me know in case you would like to discuss in further detail any of the any of the reviewer comments or your proposed revisions, I would be happy to schedule a call.

When submitting your revised manuscript, please carefully review the instructions that follow below. We perform an initial quality control of all revised manuscripts before re-review; failure to include requested items will delay the evaluation of your revision.

We require:

1) A .docx formatted version of the manuscript text (including legends for main figures, EV figures and tables). Please make sure that the changes are highlighted to be clearly visible. Alternatively you may choose to submit your manuscript as a LaTeX file.

2) Individual production quality figure files as .eps, .tif, .jpg (one file per figure). For guidance, download the 'Figure Guide PDF' (https://www.embopress.org/page/journal/17574684/authorguide#figureformat).

3) At EMBO Press we ask authors to provide source data for the main figures. Our source data coordinator will contact you to discuss which figure panels we would need source data for and will also provide you with helpful tips on how to upload and organize the files.

4) A .docx formatted letter INCLUDING the reviewers' reports and your detailed point-by-point responses to their comments. As part of the EMBO Press transparent editorial process, the point-by-point response is part of the Peer Review File (PRF), which will be published alongside your paper.

5) A complete author checklist, which you can download from our author guidelines (https://www.embopress.org/page/journal/17574684/authorguide#submissionofrevisions). Please insert information in the checklist that is also reflected in the manuscript. The completed author checklist will also be part of the PRF.

6) Please note that all corresponding authors are required to supply an ORCID ID for their name upon submission of a revised manuscript.

7) It is mandatory to include a 'Data Availability' section after the Materials and Methods. Before submitting your revision, primary datasets produced in this study need to be deposited in an appropriate public database, and the accession numbers and database listed under 'Data Availability'. Please remember to provide a reviewer password if the datasets are not yet public (see https://www.embopress.org/page/journal/17574684/authorguide#dataavailability).

In case you have no data that requires deposition in a public database, please state so in this section as follows: "This study includes no data deposited in external repositories". Note that the Data Availability Section is restricted to new primary data that are part of this study.

8) All Materials and Methods need to be described in the main text using our 'Structured Methods' format, which is required for all research articles. According to this format, the Methods section includes a Reagents and Tools Table (listing key reagents, experimental models, software and relevant equipment and including their sources and relevant identifiers) followed by a Methods and Protocols section describing the methods using a step-by-step protocol format. The aim is to facilitate adoption of the methodologies across labs. Please upload the Reagents and Tools table as a separate document when submitting your revised manuscript. More information on how to adhere to this format as well as a downloadable template (.docx) for the Reagents and Tools Table can be found in our author guidelines:
https://www.embopress.org/page/journal/17444292/authorguide#structuredmethods

An example of a Method paper with Structured Methods can be found here:

https://www.embopress.org/doi/10.15252/msb.20178071.

9) For data quantification: please specify the name of the statistical test used to generate error bars and p-values, the number (n) of independent experiments (specify technical or biological replicates) underlying each data point and the test used to calculate p-values in each figure legend. The figure legends should contain a basic description of n, p-values and the test applied. Graphs must include a description of the bars and the error bars (s.d., s.e.m.). Please provide exact p-values (in either the figure or figure legend).

10) Our journal encourages inclusion of *data citations in the reference list* to directly cite datasets that were re-used and obtained from public databases. Data citations in the article text are distinct from normal bibliographical citations and should directly link to the database records from which the data can be accessed. In the main text, data citations are formatted as follows: "Data ref: Smith et al, 2001" or "Data ref: NCBI Sequence Read Archive PRJNA342805, 2017". In the Reference list, data citations must be labeled with "[DATASET]". A data reference must provide the database name, accession number/identifiers and a resolvable link to the landing page from which the data can be accessed at the end of the reference. Further instructions are available at .

11) We replaced Supplementary Information with Expanded View (EV) Figures and Tables that are collapsible/expandable online. EV Figures should be cited as 'Figure EV1, Figure EV2" etc... in the text and their respective legends should be included in the main text after the legends of regular figures.
- For the figures that you do NOT wish to display as Expanded View figures, they should be bundled together with their legends in a single PDF file called *Appendix*, which should start with a short Table of Content. Appendix figures should be referred to in the main text as: "Appendix Figure S1, Appendix Figure S2" etc.
- Additional Tables/Datasets should be labeled and referred to as Table EV1, Dataset EV1, etc. Legends should be provided in a separate tab in case of .xls files. Alternatively, the legend can be supplied as a separate text file (README) and zipped together with the Table/Dataset file.
See detailed instructions here:
https://www.embopress.org/page/journal/17574684/authorguide#expandedview

12) Author contributions: CRediT has replaced the traditional author contributions section because it offers a systematic machine-readable author contributions format that allows for more effective research assessment. Please remove the Authors Contributions from the manuscript and use the free text boxes beneath each contributing author's name in our system to add specific details on the author's contribution. More information is available in our guide to authors.

13) Disclosure statement and competing interests: We updated our journal's competing interests policy in January 2022 and request authors to consider both actual and perceived competing interests. Please review the policy https://www.embopress.org/competing-interests and update your competing interests if necessary.

14) Every published paper now includes a 'Synopsis' to further enhance discoverability. Synopses are displayed on the journal webpage and are freely accessible to all readers. They include a short stand first (maximum of 300 characters, including space) as well as 2-5 one-sentences bullet points that summarizes the paper. Please write the bullet points to summarize the key NEW findings. They should be designed to be complementary to the abstract - i.e. not repeat the same text. We encourage inclusion of key acronyms and quantitative information (maximum of 30 words / bullet point). Please use the passive voice. Please attach these in a separate file or send them by email, we will incorporate them accordingly.

Please also suggest a striking image or visual abstract to illustrate your article as a PNG file 550 px wide x 300-600 px high.

Please note that these would be the final versions and changes during proofing are usually not allowed.

15) As part of the EMBO Publications transparent editorial process initiative (see our policy here: https://www.embopress.org/transparent-process#Review_Process), Molecular Systems Biology will publish online a Peer Review File (PRF) to accompany accepted manuscripts.
In the event of acceptance, this file will be published in conjunction with your paper and will include the anonymous referee reports, your point-by-point response and all pertinent correspondence relating to the manuscript. Let us know whether you agree with the publication of the PRF and as here, if you want to remove or not any figures from it prior to publication.
Please note that the Author checklist will be published at the end of the PRF.

Molecular Systems Biology has a "scooping protection" policy, whereby similar findings that are published by others during review or revision are not a criterion for rejection. Should you decide to submit a revised version, I do ask that you get in touch after three months if you have not completed it, to update us on the status.

Yours sincerely,

Poonam Bheda, PhD

Senior Scientific Editor
Molecular Systems Biology

Reviewer #1:

SUMMARY
In this work, Shaw et al. developed a platform to encode information in macroscopic spatial patterns generated on semi-solid agar plates by swarming E. coli, and subsequently explored various strategies to decode the information from said patterns. The authors controlled swarming in E. coli via a chemical inducer (IPTG) and optogenetic input (blue light), by overexpressing 4 different genes involved in motility. This generated colonies whose characteristics (both endpoint features and dynamic growth) encoded the input in a binary or analog fashion. Regression models and deep learning neural network models were developed to decode information from established patterns and showed high accuracy. This work provides a valuable addition to the synthetic biology toolbox to control motility in E. coli and showcases a proof of concept of how macroscopic spatial patterns can be used as environmental biosensors.

GENERAL REMARKS
-The experimental design and the resulting data are sound.

-The work is conceptually very similar to an article the group has published before: 'Engineered bacterial swarm patterns as spatial records of environmental inputs (Doshi et al 2023)'. In particular the principle of encoding and decoding information using macroscopic bacterial swarm patterns, as well as the decoding strategies used, are well described in the existing publication. The most significant advancement in this work is the use of a model organism instead of the less popular Proteus mirabilis, as well as the expansion of the toolbox to control motility (by exploring 4 different genes and 2 different control strategies, i.e. chemical inducers and optogenetics)

-Controlling swarming via optogenetics by overexpressing motility-related genes complements nicely existing examples of light-dependent motility (Light-powering Escherichia coli with proteorhodopsin, Walter et al 2007; Dynamic density shaping of photokinetic E. coli, Frangipane et al. 2018).

-The work will be useful for researchers working on bacterial motility, spatial patterns and information encoding/decoding, and will be interesting for the community of synthetic biologists.

-We appreciated the clarity and thoroughness of the text. We were particularly impressed by the level of detail with which methods are described.

MAJOR POINTS
-The authors give no explanations of the mechanisms of how expression of the 4 genes influence the pattern. It is completely treated as a black box. We understand that understanding every detail is probably outside of the scope, but there is already a lot known about E. coli motility and connecting the work presented in this manuscript with this body of literature seems essential. Where the patterns cannot be explained, at least some hypotheses to explain the observed effects of overexpression should be formulated.

To give you some specific points to be explained:
-What is the genotype of MG1655hm? What are its mutations and the mechanism to make it hypermotile?

-fliA and cheZ induction results are intriguing and counter intuitive: How is it possible that expressing lrp leads to colony expansion and fliA represses expansion if both are supposed to increase motility? Why did cheZ induction not have any effect on motility, while it previously was shown to have an effect (https://doi.org/10.1371/journal.pone.0196999, Virgile et al. 2018). Could these unexpected results be related to the MG1655hm strain?

-Line 99: "cheZ overexpression slightly decreased colony size in both uninduced and induced states but did not alter colony morphology." Why would the uninduced state change colony size? Is the construct leaky and already overexpressed in the off state?

-A brief explanation about by which mechanism rpoS could be affecting colony characteristics (such as asymetry and wispy branches) would be interesting.

-Supplementary Figure 1a: add an explanation of the biological mechanism that links glucose to swarming motility. Also clarify which are the genetic and morphologic differences between the hypermotile strain and the parent strain: did you identify mutated genes? At the microscope, does the hypermotile strain appear long, or hyperflagellated, or does it aggregate more?

-We are surprised that blue light did not have a considerable negative effect on the motility with MG1655hm pDawn-mCherry (under blue light). According to this article (https://doi.org/10.1128/jb.00762-18), blue light should decrease swimming capacity of E. coli. Is this not observed because of the experimental setup (swarming on agar, or insufficient light intensity)? Or is this because of the hypermotile strain? A simple experiment with MG1655 + pDawn-mCherry (WT, not the hm strain) under blue light could answer this question.

- It is unclear which are the potential applications of the system presented and the advantages of biological information encoding/decoding compared to traditional analogic or digital strategies. In 256-260, the authors envision the system to work as an environmental biosensor: assuming it can be engineered to detect airborne and soluble signals of interest (e.g. radiation and heavy metals, as suggested), the system would still need a camera/scanner equipped with one of the image-decoding tools to provide an effective readout, countering the argument of bio-based solutions. Or, swarm colonies would need to be physically transferred from the area of interest to an image-processing site, which is not different than collecting samples and analyzing them with e.g. mass spectrometry. The authors should briefly compare the swarm-based biosensor and traditional analogic/digital sensors and briefly comment on the trade-offs (e.g. speed, cheapness, ease of access, information amount...)

-In 175-210, the authors explore how the system encodes the input dynamically, as the colonies grow, and how timelapses allow to capture the initial conditions. Nevertheless, only scenarios where the input remains constant throughout the entire colony growth are shown. It would be important to show how the pattern changes for inputs that change dynamically (e.g. fluctuating levels of light, or increasing/decreasing concentrations of IPTG) as the colony grows. This would be representative of real case scenarios where exposure to the input is transient or the input fluctuates over time. [Estimated additional time: 1-2 months]

-It is unclear whether the tools developed are 'predictive' of conditions they have not been trained on. The author should comment on the capacity of their tools (regression models and deep learning neural network models) to interpret conditions beyond the 'training set' (e.g. an IPTG concentration intermediate between 2 concentrations tested, or a concentration 10 times larger than the highest concentration tested).

-The final Results paragraph about chromoproteins (241-245) is not particularly convincing. Chromoproteins are well known, and the work does not provide relevant addition (e.g. engineering, optimization...). Arguably, combining 3 different chromoproteins under 3 different promoters would allow to record 3 different input signals in regular colonies, independently from swarming. Does this section add something relevant to the paper? We would suggest to completely remove it from the manuscript and invest more time in the other points mentioned here in the "Major points" section.

-In Supplementary Figure 7, "...fold change relative to the non-induced (0 h) condition..."
It seems a bit unfair to compare the fluorescence of a 24h culture to a 0h, since the number of cells is much lower in 0h and only the absolute fluorescence value was taken into account, not normalized to OD600.
Comparing pDawn-mCherry with and without light, both after 24h incubation would give you a more reliable fold-change of your circuit. Also, since your motility experiments are done in agar plate, if possible, it would be preferrable that pDawn and IPTG (Supplementary Figure 1b) characterization are done in agar plate as well, instead of liquid culture.

MINOR POINTS
MAIN TEXT
-31, 65-67, 70-72) "Escherichia coli has not been engineered for robust control of swarming. [...] To date, most engineered bacterial patterning systems have relied on pre-patterned templates, quorum sensing, or swimming motility, approaches that frequently produce transient or microscale morphologies. [...] Here we leverage Escherichia coli-a genetically tractable chassis whose swarming and patterning behaviors have not been harnessed as biotechnological tools" This statement feels a bit exaggerated and minimizes the successes in the engineering of E. coli swarming and motility. Many milestones have been achieved, including coupling of swimming motility to light, microscopic patterns, macroscopic patterns with single and multiple strains. The author cites the appropriate works (references 9-17) but do not adequately acknowledge their contribution to the SynBio field. The same could be said for patterning, as E. coli has been largely used for countless patterns, at the micro and macro scale, leveraging different strategies.

-81-82) "regular, visually distinct, and resolved with low-cost, flatbed imaging" -> not clear what regular and visually distinct mean. Radially symmetric, homogeneous, smooth? Distinct from what? Maybe 'discernible to the naked eye' or 'distinguishable by the naked eye' are more appropriate? For low-cost flatbed imaging, refer to Methods, Swarming assays section.

-82) "Screening MG1655 and genetic variants identified" -> missing subject. Either 'Screening, we identified...', or 'A screening allowed us to identify...' . Also, 'MG1655 strain and its genetic variants' might be more appropriate.

-162) "SwinTransformer" -> move reference 32 next to the tool name and briefly clarify what it is (e.g. the hierarchical vision transformer SwinTransformer, or the Microsoft image manipulation tool SwinTransformer)

-169) "accuracies of 92% and 82%, top-2 accuracies of 96% and 88%, and AUCs of 0.99 and 0.89, respectively." -> while the conveyed message is clear, the reader might not be familiar with the definitions of accuracy, top-2 accuracy and AUC metrics.

Consider adding a brief definition in the Methods or including a reference.

-232-233) "With pDawn, stronger gene expression could be due to combined effects of high-intensity blue light and the reported high strength of the λ pR promoter (or other elements of the pDawn system)". -> Have you tried to reduce blue light intensity, or to use periodic exposure (e.g. 1min ON - 3min OFF) instead of continuous illumination?

-276) "E. coli Mach1" -> add (ThermoFisher) if appropriate

-388) "an incubator shelf" -> it is unclear how the light goes through the shelf. Is it a transparent shelf?

-757-758) why did you use 10mM IPTG for some strains and 2mM for others? Clarify or include in supplementary?

-773) "false-color colony masks" -> it unclear what a colony mask represents here and how it is used for the analysis. Simply refer to Methods, section Colony segmentation, or to Supplementary Figure 2a

-781-782) "Representative masked polar-coordinate colony images at 0.05 mM IPTG are shown." -> specify in the caption what the dotted line and the two arrows represent in the lrp image (Fig 2d).

-Are the features observed by the neural network model the same as the ones the authors used for the regression model?

-It would be better if you could measure the light intensity given in a commonly used unit, like W/m2 or mW/cm2.

-The community would benefit most from your work if you provide all the constructs of this work on Addgene.

-It is nice that the methods are very detailed. However, in case space is an issue, standard methods like preparation of competent cells and electroporation could be removed. Most lab have those methods already established and unless we are mistaken, doing it slightly different should not prevent reproducibility of your results.

-A suggestion for future work: You could test the light-inducible motility in an analog rather than a binary manner. This way, you could infer the light intensity from the colony size. Next, you could engineer additional strains that respond to green and red light. In your experiments, you would inoculate three colonies on the same plate, each one responsive to a different light color. This setup would allow you to determine the light composition and intensities based on the relative sizes of the three colonies.

-In an attempt to promote sustainable research practices, we encourage setting ultralow temperature freezers at -70{degree sign}C instead of -80{degree sign}C.

SUPPLEMENTARY MATERIAL
-Supplementary Figure 1b: reconsider if it is appropriate to confront the Total fluorescence quantified as area under each trajectory (which represents a response over time), instead of the endpoint fluorescence intensity. For example, a trajectory that spikes to 500a.u. at 9h and then falls to 0a.u. at 18h would have the highest Total fluorescence, but the lowest endpoint fluorescence. If the rationale of the experiment is to choose the ideal IPTG concentration to see differences in endpoint patterns, the endpoint fluorescence intensity should be used to quantify fold-change.
-Supplementary Figure 3: consider combining with Figure 3 in the main text. Show just one row of pLac-gfp control swarm pattern in Fig 3a, and add the corresponding colony mask in Fig 3b. This would avoid switching back and forth from Supplementary Information.
-Supplementary Figure 7a: clarify whether 'Fluorescence trajectory' refer to the area under each trajectory as in Supplementary Figure 1b

Reviewer #2:

Summary
This paper explores how E. coli swarming patterns can be engineered and interpreted as information-encoding devices. The authors construct a hypermotile E. coli strain (MG1655hm) and introduce IPTG-inducible overexpression circuits for several motility-related genes (lrp, fliA, and rpoS). Varying IPTG levels produces distinct macroscopic patterns: a sharp, threshold-like (binary) response for lrp and gradual (analog) responses for fliA and rpoS. Using quantitative image features (colony area, Fourier spectra, eccentricity, etc.), the authors train classifiers and deep learning models to decode IPTG concentration from colony morphology. They further analyze temporal dynamics using a flatbed scanner for high-resolution time-lapse imaging, and they demonstrate modularity by replacing IPTG induction with a blue-light-inducible fliA circuit.

Overall, the paper presents an elegant integration of genetic engineering, quantitative imaging, and machine learning. The experimental setup is creative and the data are analyzed rigorously through well-documented pipelines. Figures are well organized (though a bit faint and hard to read on my computer), and the authors convincingly demonstrate that engineered

swarming can record and decode environmental inputs.

However, several aspects could be better clarified and elaborated as detailed below.

Comments

1. The authors emphasize that using E. coli provides a more genetically tractable chassis compared to Proteus mirabilis, featured in their previous Nat. Chem Bio paper (Doshi et al., 2023). However, the conceptual advance here is not fully articulated. The manuscript would benefit from explicitly explaining what new principles or capabilities emerge from this transition, e.g., the ability to tune pattern morphologies via specific regulatory genes (lrp, fliA, rpoS), to modulate swarming hierarchically, or to couple to optogenetic inputs. Also, the authors should note some related publications that demonstrate the use of phenotypic traits (growth/gene expression) to decode input signals, including: Duncker et al, BioRxiv 2025, Engineering microbial consortia for distributed signal processing

2. The decoding accuracies (AUC up to 1.0) appear impressive but possibly optimistic given modest sample sizes (n = 9-35 colonies per condition). Although data augmentation and held-out testing are described, the paper should discuss potential overfitting and model generalization across biological replicates and experimental days. Demonstrating decoding of an independent dataset or cross-day validation would substantially increase confidence in the robustness of the classifiers.

3. With the rationale of using temporal features to better support decoding, it would have been nice to see how expansion rate or textural uniformity improves AUC of -rpos, given the caveat that there is little room for improvement of -lrp and -fliA since the prediction performance from spatial features alone is quite high already.

4. The pDawn-fliA experiments successfully illustrate modularity in input sensing but remain preliminary. The demonstration is limited to a binary comparison (red vs. blue light) without a corresponding decoding analysis or analog gradient. As written, this section feels disconnected from the rest of the paper. Expanding this part to include a quantifiable light-dose response or prediction of illumination time from colony patterns would make it more cohesive.

5. The decoding tasks were trained separately for each engineered strain. It would strengthen the generality of the approach to test whether a single model can classify both IPTG concentration and strain identity. This would demonstrate that the learned morphological features reflect underlying biological structure rather than dataset-specific cues-an important step toward scaling to multiplexed sensing applications.

6. I did not understand why having a digital-like response for the lrp strain, wherein the patterns seem similar till a certain threshold and similar after that threshold is leading to a better classification performance. The analog behavior(fliA and rpoS strains), wherein the change is more gradual and conditions can be seemingly distinguished by the naked eye has a lower performance. This is reflected in both the machine learning and deep learning pipelines.

7. Figures 2 and 3 use distinct sets of features for binary and analog strains. While the Supplementary Tables list these, a short statement in the main text explaining that feature sets were optimized separately for each strain would reduce confusion.

8. Panels (c-h) of Figure 4, focusing on spatial and temporal feature analysis, are conceptually separate from the machine- and deep-learning components in panels (a-b). The paper would flow more clearly if these were placed in a separate figure or moved to the Supplementary section.

9. For their inference pipeline, the authors also used a deep-learning based approach to bypass human-guided extraction of features. I very much like the approach as I in principle agree with the rationale - the model should be able to learn relevant features (whether or not they're human interpretable) that are important for prediction. Overall, it appears that they achieved comparable performances. I think it's valuable to discuss a bit more the pros and cons of each approach.

10. I wish they had deposited the data and codes to a public repository. Regardless, I'd encourage the authors to do so at least when the paper is accepted for publication.

Other minor suggestions.

Line 83: Provide a citation or note for the origin or construction of the hypermotile E. coli strain.

Line 101: Clarify how fliA overexpression, which enhances flagellar gene expression, mechanistically leads to reduced swarming. Are swimming and swarming oppositely regulated in this context?

Line 110: Include representative replicate colony images (e.g., in Supplementary Information) to show the reproducibility of the observed morphologies.
Line 126: Explain why different IPTG concentration ranges were used for fliA and rpoS experiments-was this based on empirical optimization or sensitivity differences?
Line 224: Add a brief rationale for choosing fliA as the gene for optogenetic control.
Line 244: Move speculative discussion about future chromoprotein applications to the Discussion section.

Reviewer #3:

This work introduces a synthetic biology platform that employs engineered swarming motility of E. coli to spatially record chemical and optical inputs as visual-scale colony morphologies. By modulating key motility regulators, the authors establish a 'visual language' of patterns that they aim to decode using a computational pipeline integrating texture analysis and deep learning.

I like, in theory, the idea of modulating the swarm behavior of bacteria using genetic switches and dials. I like the promise of the work, additionally. I have no considerable issues with the design, performance or portrayal of the work.

Specific points:
In fig3, images of the GFP control should be included in the main figure, especially given that the quantitative descriptions in the plot below includes them.

General comments:
This work establishes a fundamental ability to engineer macroscopic swarm patterns, but it essentially operates as a technical proof-of-concept. To demonstrate true utility, the authors need to show the system working in a relevant application beyond simple induction assays (such as metal sensing as authors exemplify in Discussion section).

There is a notable inconsistency in the depth of analysis between the chemical and optical recording sections. While the chemical recording utilizes robust, multi-dimensional feature extraction, the optical recording is left to primarily rely on 'minimum colony radius.'
This is understandably due to the difference in the nature of the promoters. However, this kind of biological 'quirk' is also what elevates the need of demonstration of applicability and scalability over the demonstration of concept.
This reduction to a single metric makes the optical system more susceptible to experimental artifacts and less distinct than the high-dimensional 'visual language' established earlier in the paper. A consistent application of the computational pipeline to the optical data is needed to validate the system's robustness.
The work could have benefited from experimenting with varying light intensities or periodic duty cycles. Without tunability, I fail to perceive the utility of light based sensors - a very crude parallel could be exposure to anything that inhibits swarming thus eliciting a binary response.

The inclusion of the chromoprotein data (pg 9, supp. fig 8) feels disconnected from the manuscript's primary focus on morphological recording. The sudden introduction of color as an output channel, even though described by the authors themselves as 'preliminary' and 'briefly explored', lacks the depth of characterization seen in the swarming experiments. Because these colored strains are not integrated into the computational decoding pipeline, this section appears as an add-on that dilutes the cohesion of the main story rather than enhancing the proof-of-concept.

Swarming itself offers unique advantages: it produces macroscale readouts on timescales comparable to fluorescent reporters" - in the discussion section sounds like an overstatement. Swarming, technically, is a growth-dependent process resulting in a macroscopic endpoint (typically 24 hours), (even if one would restrict the observations to colony expansion, it is still a long process), whereas fluorescent reporters can potentially enable real-time dynamic monitoring (for e.g. doi = 10.1038/s41592-022-01597-x). The limited 'early detection' data provided for IPTG induction also, is insufficient to claim temporal equivalence with fluorescent methods.

Concluding, the work should include demonstration of applicability beyond the proof of concept, such as an environmental sensing task, and must optimize the optical circuit to achieve the claimed analog recording capability, which currently appears limited by leakiness and a reliance on only binary inputs.

Response to Reviewers
Manuscript Number: MSB-2025-13399-T
Title: *Engineered E. coli swarming for binary and analog input recording*

Reviewer #1:

SUMMARY
In this work, Shaw et al. developed a platform to encode information in macroscopic spatial patterns generated on semi-solid agar plates by swarming E. coli, and subsequently explored various strategies to decode the information from said patterns. The authors controlled swarming in E. coli via a chemical inducer (IPTG) and optogenetic input (blue light), by overexpressing 4 different genes involved in motility. This generated colonies whose characteristics (both endpoint features and dynamic growth) encoded the input in a binary or analog fashion. Regression models and deep learning neural network models were developed to decode information from established patterns and showed high accuracy. This work provides a valuable addition to the synthetic biology toolbox to control motility in E. coli and showcases a proof of concept of how macroscopic spatial patterns can be used as environmental biosensors.

We thank the reviewer for this clear and thoughtful summary of our work. We are encouraged that the reviewer recognizes the potential of our platform as a spatial biosensing strategy. As detailed below, we have further strengthened the manuscript by expanding optical recording capabilities, exploring spatiotemporal recording, and introducing multiplexed decoding across strains and input concentrations.

GENERAL REMARKS
-The experimental design and the resulting data are sound.

We thank the reviewer for this positive assessment.

-The work is conceptually very similar to an article the group has published before: 'Engineered bacterial swarm patterns as spatial records of environmental inputs (Doshi et al 2023)'. In particular the principle of encoding and decoding information using macroscopic bacterial swarm patterns, as well as the decoding strategies used, are well described in the existing publication. The most significant advancement in this work is the use of a model organism instead of the less popular Proteus mirabilis, as well as the expansion of the toolbox to control motility (by exploring 4 different genes and 2 different control strategies, i.e. chemical inducers and optogenetics)

We appreciate the reviewer’s comparison and agree that this work extends our earlier framework by implementing it in a widely used model organism and by incorporating an expanded set of inducible motility-related genes under both chemical and optogenetic control. In addition, this study introduces both binary- and analog-like modes of recording, along with expanded computational decoding approaches, enabling more flexible and scalable information readout from swarm morphologies.

-Controlling swarming via optogenetics by overexpressing motility-related genes complements nicely existing examples of light-dependent motility (Light-powering Escherichia coli with proteorhodopsin, Walter et al 2007; Dynamic density shaping of photokinetic E. coli, Frangipane et al. 2018).

We thank the reviewer for this observation and agree that these studies provided valuable context and inspiration for our optogenetic control of swarming. We now cite these works in the Results section.

-The work will be useful for researchers working on bacterial motility, spatial patterns and information encoding/decoding, and will be interesting for the community of synthetic biologists.

We appreciate the reviewer's positive assessment and the potential utility of the work is clear.

-We appreciated the clarity and thoroughness of the text. We were particularly impressed by the level of detail with which methods are described.

We thank the reviewer for these encouraging comments and are pleased that the clarity and level of detail in our methods were appreciated.

MAJOR POINTS

-The authors give no explanations of the mechanisms of how expression of the 4 genes influence the pattern. It is completely treated as a black box. We understand that understanding every detail is probably outside of the scope, but there is already a lot known about E. coli motility and connecting the work presented in this manuscript with this body of literature seems essential. Where the patterns cannot be explained, at least some hypotheses to explain the observed effects of overexpression should be formulated.

We thank the reviewer for this helpful suggestion—we agree that linking the observed swarm phenotypes to the established *E. coli* motility regulatory network strengthens the interpretation of the results. In the revised Results section, we have expanded the paragraph introducing the motility network (Figure 1B), providing additional mechanistic context through more detailed description and clarification of prior literature already cited in the original manuscript. Specifically, we clarify the Class I–III structure of the flagellar transcriptional cascade; we describe *lrp* as encoding the leucine-responsive regulatory protein that activates *flhDC*, the Class I master regulator of flagellar gene expression; and we specify that *fliA* encodes the flagellum-specific sigma factor σ^{28} that activates transcription of Class III genes for flagellar assembly and chemotaxis. We also describe *rpoS* as the global stress-response sigma factor that represses flagellar gene expression, and we expand the description of the chemotaxis gene *cheZ* to note that it encodes a phosphatase that promotes forward runs by reducing tumble bias through rapid signal termination. Finally, we have added hypotheses linking these regulatory roles to the observed swarm phenotypes when we introduce Figure 1C, including additional supporting references alongside prior literature cited above.

To give you some specific points to be explained:

-What is the genotype of MG1655hm? What are its mutations and the mechanism to make it hypermotile?

We thank the reviewer for requesting clarification on the genotype, mutations, and motility mechanisms of the hypermotile strain (MG1655hm). In the revised Methods and Protocols section (*Bacterial strains, storage, and growth conditions*), we have added the following information describing the strain's genetic background. MG1655hm corresponds to "MG1655(motile)" from the Coli Genetic Stock Center (CGSC; strain #8237). It is an isolate of MG1655 (CGSC #6300) that was selected and verified for motility. Previous studies have reported that CGSC #8237 and other motile *E. coli* K-12 derivatives carry an IS5-element insertion upstream of the *flhD* promoter, which activates the *flhDC* master motility regulon and increases swarming motility (Barker et al., 2004; Pleška et al., 2021). We have also clarified the strain identifier when first introducing MG1655hm in the Results section ("MG1655hm; CGSC #8237").

-fliA and cheZ induction results are intriguing and counter intuitive: How is it possible that expressing lrp leads to colony expansion and fliA represses expansion if both are supposed to increase motility? Why did cheZ induction not have any effect on motility, while it previously was shown to have an effect (https://doi.org/10.1371/journal.pone.0196999, Virgile et al. 2018). Could these unexpected results be related to the MG1655hm strain?

We thank the reviewer for raising these points and questions. Although both *lrp* and *fliA* are associated with activation of motility-related genes, they act at different levels of the flagellar regulatory hierarchy. *lrp* functions upstream by activating *flhDC*, the master regulator that broadly initiates the flagellar transcriptional cascade, which is consistent with the increased swarm expansion observed upon *lrp* induction. *fliA*, on the other hand, encodes the flagellum-specific sigma factor σ^{28} that activates transcription of late flagellar assembly and chemotaxis genes. Overexpression of *fliA* may therefore drive strong activation of these downstream motility genes independently of upstream regulatory coordination. Because swarming requires coordinated regulation across the motility cascade, excessive activation of late flagellar genes may disrupt this balance or increase the energetic burden associated with flagellar biosynthesis (Fitzgerald et al., 2014), ultimately limiting colony expansion, as we observed upon *fliA* induction in MG1655hm.

Regarding *cheZ*, Virgile et al. (2018) examined CheZ-dependent changes in swimming behavior. Their experiments were performed in a Δ*cheZ* background complemented with inducible *cheZ* expression and were evaluated under low-agar motility conditions (0.25% agar), which support swimming rather than canonical swarming. In contrast, our experiments measure collective swarming on semi-solid surfaces in a hypermotile MG1655 background (MG1655hm) that already contains endogenous *cheZ*. Previous work has shown that motility depends on maintaining an optimal tumble bias controlled in part by CheZ levels, and that increasing CheZ expression beyond this optimal range can reduce motility or swarm expansion (Partridge et al., 2019; Virgile et al., 2018). Because MG1655hm already expresses *cheZ*, additional overexpression may therefore provide limited benefit and may even mildly reduce colony expansion by pushing tumble bias outside the range optimal for coordinated surface migration, as observed in our pLac-*cheZ* swarming assays (Appendix Figure S3). The slightly reduced colony size observed in the uninduced condition is consistent with mild basal leakiness of the pLac promoter, which would produce low-level *cheZ* overexpression even in the absence of IPTG. Overall, *cheZ* induction produced only modest changes in colony size. We have clarified these interpretations of *lrp*, *fliA*, and *cheZ* overexpression in the revised Results section alongside Figure 1B,C.

As an additional supporting analysis, and focusing on *lrp* due to its clear enhancement of swarm expansion, we performed complementary swimming assays under quasi-two-dimensional microscopy conditions (Reviewer Figure 1). We observed a significant increase in swimming speed in MG1655hm pLac-*lrp* upon IPTG induction, unlike in the wild-type and pLac-*gfp* control strains under identical conditions. While swimming and swarming represent distinct modes of motility, these results indicate that *lrp* induction is associated with increased motility in both single-cell and collective contexts. This correspondence also suggests that, in some cases, microscopy-based swimming assays could serve as a complementary approach to characterize motility phenotypes and guide circuit design prior to more extensive characterization using macroscale swarm-based readouts.

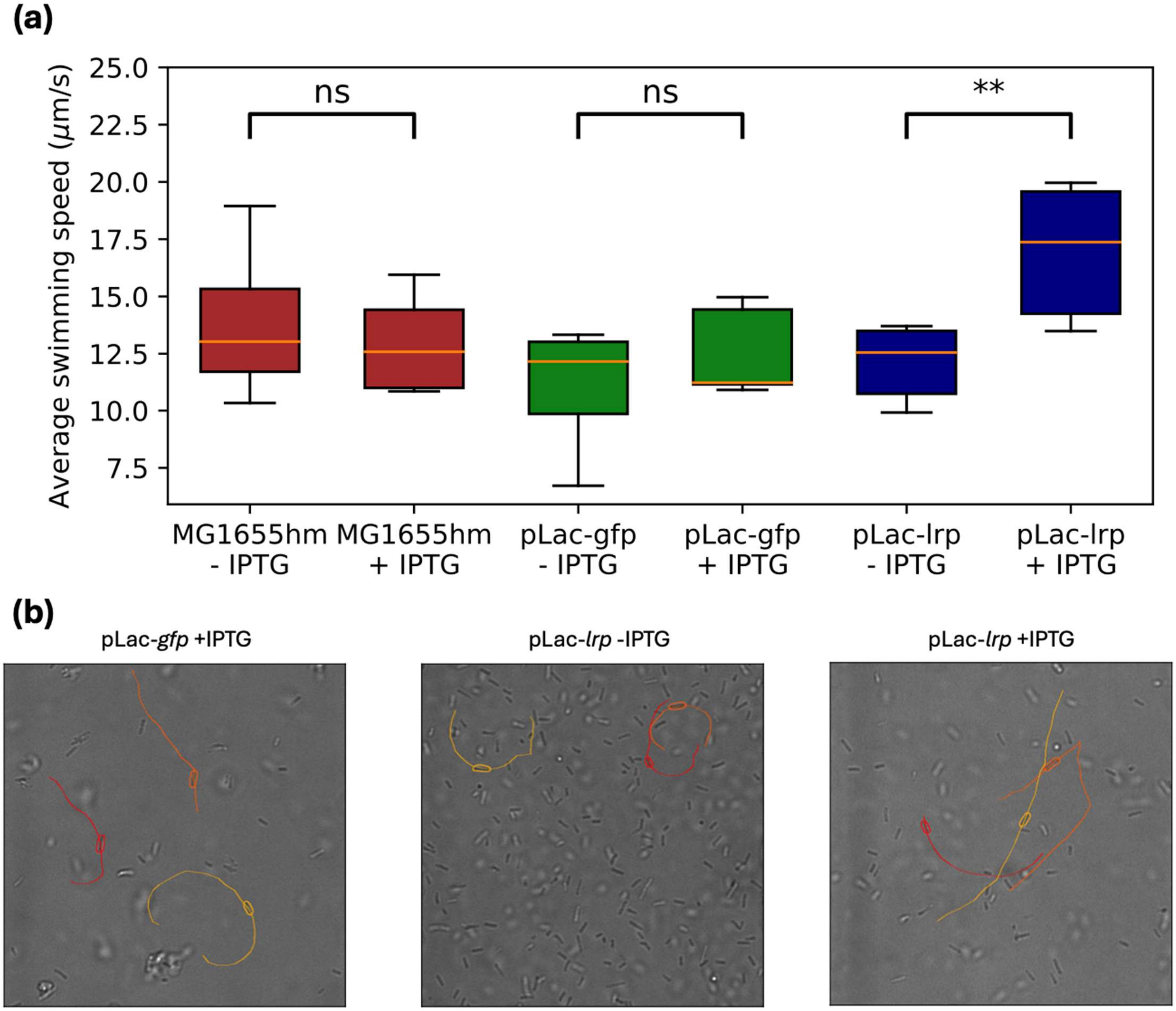

Reviewer Figure 1 | *lrp* induction increases single-cell swimming speed. (a) MG1655hm wild-type, pLac-*gfp*, and pLac-*lrp* strains were subcultured with or without IPTG (1 mM) and transferred to quasi-two-dimensional microscopy conditions. Cells were imaged by time-lapse brightfield microscopy (0.1 s intervals). Images were analyzed using custom Python code, with segmentation performed using a custom-trained Cellpose 2.0 model. For each condition, six cells were tracked for ≥10 frames, and swimming speeds were computed from the time evolution of centroid positions. Induction with IPTG significantly increased swimming speed in MG1655hm pLac-*lrp*, but not in the wild-type or pLac-*gfp* control strains. Boxplots show the distribution of cell velocities (n = 6 cells per condition). Two-sample t-test ($P < 0.01$; ns, not significant). **(b)** Representative microscopy images of MG1655hm pLac-*gfp* with IPTG (left), pLac-*lrp* without IPTG (center), and pLac-*lrp* with IPTG (right). Each example frame is overlaid with segmentations of three tracked cells and their corresponding trajectories used for analysis.

-Line 99: "cheZ overexpression slightly decreased colony size in both uninduced and induced states but did not alter colony morphology." Why would the uninduced state change colony size? Is the construct leaky and already overexpressed in the off state?

We thank the reviewer for this observation and these questions. The reduced pLac-*cheZ* colony size observed in the uninduced condition is consistent with mild basal leakiness of the pLac promoter, which can produce low-level *cheZ* expression even in the absence of IPTG. In our system, *cheZ* is overexpressed in a hypermotile MG1655 background (MG1655hm) that already contains endogenous *cheZ*. Previous work has shown that motility depends on maintaining an optimal tumble bias controlled in part by CheZ levels, and that increasing CheZ expression beyond this optimal range can reduce motility or swarm expansion (Partridge et al., 2019; Virgile et al., 2018). Thus, even modest increases in CheZ expression due to basal promoter leakiness could slightly reduce colony expansion by shifting tumble bias outside the range optimal for coordinated swarming. We have clarified this interpretation in the revised Results section (Figure 1C).

-A brief explanation about by which mechanism rpoS could be affecting colony characteristics (such as asymmetry and wispy branches) would be interesting.

We thank the reviewer for this suggestion. We have added a brief mechanistic explanation linking *rpoS* overexpression to the observed swarming phenotype in the revised Results section alongside Figure 1B,C. *rpoS* encodes the global stress-response sigma factor σ^S, which represses the flagellar gene-expression pathway under stress conditions. Repression of this transcriptional hierarchy can limit coordinated outward swarm expansion, potentially destabilizing the advancing colony front and producing shorter, sparse protrusions at the colony edge, which may explain the wispy branching morphology observed upon *rpoS* induction in MG1655hm.

-Supplementary Figure 1a: add an explanation of the biological mechanism that links glucose to swarming motility. Also clarify which are the genetic and morphologic differences between the hypermotile strain and the parent strain: did you identify mutated genes? At the microscope, does the hypermotile strain appear long, or hyperflagellated, or does it aggregate more?

We thank the reviewer for these helpful suggestions and questions, which we have clarified in the revised manuscript. First, we have now briefly described the role of glucose in supporting swarming motility when we parenthetically reference Appendix Figure S1A in the revised Results. Specifically, we note that glucose supports flagella-driven swarming motility in *E. coli* by fueling cellular energy metabolism and the production of surface-wetting factors required for colony expansion (Inoue et al., 2006)

Second, the hypermotile host strain which we refer to as MG1655hm corresponds to the “MG1655(motile)” strain from the Yale Coli Genetic Stock Center (CGSC; strain #8237), as we note in the revised Methods and Protocols. This strain is an isolate of the parent MG1655 strain (CGSC #6300) that was selected and verified for enhanced motility. Previous studies have reported that CGSC #8237 and other motile *E. coli* K-12 derivatives carry an insertion sequence element (IS5) upstream of the *flhD* promoter, which activates transcription of the *flhDC* master regulator of flagellar biosynthesis and motility and thereby increases swarming motility (Pleška et al., 2021; Barker et al., 2004).

Regarding cellular morphology at the microscale, surface-induced differentiation of *E. coli* into swarming cells involves a 2–3x increase in peritrichous flagella. Cells actively swarming outward at the colony periphery are often elongated (5-20 µm long, up from 2-4 µm), multinucleate, and devoid of septa (Harshey and Matsuyama, *PNAS* 1994; PMID: 8078935). Swarming bacteria can also move collectively in transient groups called rafts, although such aggregation is not required for swarming and may arise passively from physical interactions among neighboring cells (Kearns, 2010). To clarify these morphological features for readers, we

have slightly revised the Introduction to explicitly refer to cell elongation and hyperflagellation as key characteristics of swarming cells.

-We are surprised that blue light did not have a considerable negative effect on the motility with MG1655hm pDawn-mCherry (under blue light). According to this article (https://doi.org/10.1128/jb.00762-18), blue light should decrease swimming capacity of E. coli. Is this not observed because of the experimental setup (swarming on agar, or insufficient light intensity)? Or is this because of the hypermotile strain? A simple experiment with MG1655 + pDawn-mCherry (WT, not the hm strain) under blue light could answer this question.

We thank the reviewer for this thoughtful comment and for highlighting the study by Perlova et al., which reported blue-light–dependent modulation of *E. coli* swimming behavior. Importantly, that work examined single-cell swimming in liquid, where blue light acts as a chemotactic stimulus that alters run–tumble dynamics. In contrast, our experiments measure swarming, a multicellular mode of surface motility on semi-solid agar that depends on physiological differentiation, surfactant production, and coordinated group movement.

Partridge et al. (*mBio* 2019) showed that, during swarming, *E. coli* remodels its chemotaxis signaling pathway, resulting in altered run–tumble dynamics characterized by extended runs, reduced tumble bias, and increased speeds optimized for colony expansion. In addition, our MG1655hm host strain, like other motile *E. coli* K-12 derivatives, carries an IS5-element insertion upstream of the *flhD* promoter that elevates *flhDC* expression and swarming motility (Barker et al., 2004; Pleška et al., 2021). Together, these differences in chemotaxis signaling and motility regulation in swarming populations could buffer moderate blue-light–dependent perturbations that are more apparent in single-cell swimming assays.

Consistent with the reviewer's expectation, our quantitative analysis does show a slight decrease in colony expansion for the MG1655hm pDawn-*mCherry* control strain under increasing blue-light intensity (Revised Figure 6C). However, these differences were not statistically significant under our experimental conditions and were minor compared with the pronounced colony-scale morphological changes observed in MG1655hm pDawn-*fliA*, which we use to record the presence of blue light.

Regarding illumination, we validated the same high–blue-light condition used in the swarming assays in liquid culture. This condition produced robust induction of pDawn-driven *mCherry* expression, confirming that the colonies received sufficient blue-light exposure to activate the optogenetic system (Appendix Figure S5).

- It is unclear which are the potential applications of the system presented and the advantages of biological information encoding/decoding compared to traditional analogic or digital strategies. In 256-260, the authors envision the system to work as an environmental biosensor: assuming it can be engineered to detect airborne and soluble signals of interest (e.g. radiation and heavy metals, as suggested), the system would still need a camera/scanner equipped with one of the image-decoding tools to provide an effective readout, countering the argument of bio-based solutions. Or, swarm colonies would need to be physically transferred from the area of interest to an image-processing site, which is not different than collecting samples and analyzing them with e.g. mass spectrometry. The authors should briefly compare the swarm-based biosensor and traditional analogic/digital sensors and briefly comment on the trade-offs (e.g. speed, cheapness, ease of access, information amount...)

We thank the reviewer for this thoughtful perspective on potential applications and comparisons with traditional sensors, which we now further address in the revised Discussion. Specifically, we note that conventional sensors provide rapid and continuous measurements, whereas swarm colony development occurs over longer timescales. Although this speed may limit real-time detection, it creates the potential to integrate environmental inputs over the course of colony growth, producing a cumulative, visual record of conditions. The system could be adapted for faster readouts by tuning growth conditions (*e.g.*, lower agar density or increased surfactants), and for threshold-based ON/OFF readouts through circuit design, with more quantitative decoding possible over

longer timescales. In addition, the large populations of cells contributing to swarm pattern formation —often organized in radially symmetric expansions—effectively provide repeated measurements across space, in contrast to single-point measurements, which may enhance robustness and signal-to-noise in decoded outputs.

We highlight that swarming generates durable, centimeter-scale colony patterns that are visually distinct and readily observable without specialized instrumentation. This enables the use of simple platforms such as Petri dishes and basic imaging devices, facilitating interpretability and accessibility. We envision that these features could enable in situ monitoring through direct or remote imaging, without requiring sample collection and transfer to centralized analytical facilities.

Overall, this swarm-based platform is intended to complement, rather than replace, conventional electronic or analytical sensors. While it introduces trade-offs—including slower response times and the need for image-based decoding—it offers a biologically integrated mode of information storage that may be advantageous in low-resource or field-deployable sensing contexts. Beyond environmental biosensing, we also note potential applications in therapeutics and engineered living materials.

-In 175-210, the authors explore how the system encodes the input dynamically, as the colonies grow, and how timelapses allow to capture the initial conditions. Nevertheless, only scenarios where the input remains constant throughout the entire colony growth are shown. It would be important to show how the pattern changes for inputs that change dynamically (e.g. fluctuating levels of light, or increasing/decreasing concentrations of IPTG) as the colony grows. This would be representative of real case scenarios where exposure to the input is transient or the input fluctuates over time. [Estimated additional time: 1-2 months]

We thank the reviewer for this important suggestion to explore dynamically varying inputs during colony growth. **We have added a new experiment in which we introduced IPTG during swarm expansion as a transient, spatially localized perturbation (~12 h after inoculation; Revised Figure EV5).** In this setting, the input conditions changed over space and time, and the resulting colony morphologies reflected both the timing and location of the input perturbation. We observed asymmetric changes in radial expansion in the pLac-*rpoS* strain, whereas the control pLac-*gfp* strain showed minimal deviation. These results demonstrate that engineered swarm colonies can record temporally and spatially varying inputs during growth, extending beyond static induction conditions and supporting the applicability of this platform to dynamically changing environmental signals.

-It is unclear whether the tools developed are 'predictive' of conditions they have not been trained on. The author should comment on the capacity of their tools (regression models and deep learning neural network models) to interpret conditions beyond the 'training set' (e.g. an IPTG concentration intermediate between 2 concentrations tested, or a concentration 10 times larger than the highest concentration tested).

We thank the reviewer for this important question regarding the predictive capacity of our models beyond the training set. In this study, we trained and evaluated both regression and deep learning models on discrete input conditions (*i.e.*, defined IPTG concentrations) that we could generate in the laboratory. Accordingly, model performance reflects decoding within this experimentally sampled range, and we did not explicitly evaluate interpolation between or extrapolation beyond the tested conditions.

For the regression models, we used continuous morphological features as inputs, which may support interpolation across input levels in the future. For the deep-learning models, we trained classifiers on discrete IPTG concentration labels and therefore did not design them to predict intermediate or out-of-range conditions. However, deep-learning frameworks can be adapted for continuous prediction (Doshi et al., 2023), and future approaches such as generative image models may help expand training coverage beyond experimentally accessible conditions. In the revised Discussion section, we note that extending our computational decoding

approaches to continuous prediction across intermediate or previously unseen input levels represents an important direction for future work.

-The final Results paragraph about chromoproteins (241-245) is not particularly convincing. Chromoproteins are well known, and the work does not provide relevant addition (e.g. engineering, optimization...). Arguably, combining 3 different chromoproteins under 3 different promoters would allow to record 3 different input signals in regular colonies, independently from swarming. Does this section add something relevant to the paper? We would suggest to completely remove it from the manuscript and invest more time in the other points mentioned here in the "Major points" section.

We thank the reviewer for this helpful suggestion. We agree that the chromoprotein section, as previously presented, was preliminary and did not substantially contribute to the manuscript's central focus on swarm-based information recording and computational decoding. Accordingly, we have removed this paragraph and the corresponding supplementary figure from the revised Results section. To retain the broader conceptual point without interrupting the main narrative, we have kept a brief mention in the revised Discussion of chromoproteins as a potential future avenue for expanding visual output channels.

-In Supplementary Figure 7, "...fold change relative to the non-induced (0 h) condition..."
It seems a bit unfair to compare the fluorescence of a 24h culture to a 0h, since the number of cells is much lower in 0h and only the absolute fluorescence value was taken into account, not normalized to OD600.
Comparing pDawn-mCherry with and without light, both after 24h incubation would give you a more reliable fold-change of your circuit. Also, since your motility experiments are done in agar plate, if possible, it would be preferrable that pDawn and IPTG (Supplementary Figure 1b) characterization are done in agar plate as well, instead of liquid culture.

For endpoint fluorescence analysis: We thank the reviewer for raising these points and apologize for any confusion. In the experiment presented in Appendix Figure S5a (previously Supplementary Figure 7a), all cultures were measured at the same experimental endpoint following a fixed 24-h incubation period; the "0 h" condition refers to the absence of blue-light exposure, not to a shorter culture duration.

Cultures were exposed to blue light for 0, 1, 2, 3, 4, or 24 h and were then grown in the dark for the remainder of the 24-h period (*e.g.*, 1 h illumination followed by 23 h in the dark; 2 h illumination followed by 22 h in the dark; 3 h illumination followed by 21 h in the dark; 4 h illumination followed by 20 h in the dark). Cultures receiving 0 h illumination were grown entirely in the dark, whereas cultures receiving 24 h illumination were exposed to blue light for the full growth duration prior to measurement. Fluorescence and OD_{600} were measured simultaneously for all samples at the common 24-h endpoint. This protocol is described in detail in Methods and Protocols (*Liquid-culture induction assays*). We have revised the caption of Appendix Figure S5 to explicitly clarify the fixed total culture time and the meaning of the 0 h illumination condition.

Additionally, endpoint fluorescence values shown in the left plot of Appendix Figure S5 were first normalized to OD_{600}, thereby accounting for differences in cell density across conditions. The fold changes shown in the right plot were calculated from these OD_{600}-normalized endpoint fluorescence values relative to the 0 h illumination condition. These preprocessing and normalization steps are also described in detail in Methods and Protocols (*Liquid-culture induction analysis*).

For liquid vs. agar characterization: We thank the reviewer for this thoughtful suggestion. The purpose of Appendix Figures S1B,D and S5A (previously Supplementary Figures 1b,d and 7a) was to verify that the IPTG- and blue-light–inducible promoters behaved as expected across induction levels. Liquid-culture assays provided a standardized and quantitative framework to readily assess promoter activity prior to incorporating these regulatory elements into swarming circuits.

Importantly, fluorescence is not used as a final readout of the swarming system. The strains that generate the information-recorded patterns (pLac-*lrp*, -*fliA*, -*rpoS*, and pDawn-*fliA*) do not encode fluorescent reporters; all recorded information in this work arises from altered colony morphology rather than fluorescence intensity. As such, agar-based fluorescence measurements would not directly inform interpretation of the swarming phenotypes.

A related consideration is whether overexpression of any protein—including a fluorescent reporter—could itself perturb colony formation. We tested this directly using the pLac-*gfp* and pDawn-*mCherry* circuits, which encode fluorescent proteins under the same promoter architectures as the experimental swarming circuits. Across the IPTG and illumination conditions used in subsequent swarming assays, *gfp* and *mCherry* induction did not appreciably alter qualitative swarm morphology or quantitative image features (Figures 2, 3, 6; Appendix Figure S2). Similarly, induction of the chemotaxis gene *cheZ* did not notably alter colony morphology. These results indicate that overexpression alone does not measurably perturb swarm pattern formation at the macroscale; rather, modulation of specific swarming-related regulators is required. Consequently, fluorescent protein circuits (pLac-*gfp* and pDawn-*mCherry*) serve as appropriate matched controls for inducible expression in the corresponding swarming strains. While agar-based fluorescence imaging can be informative when fluorescence is the primary output, prior work showed only minimal, if any, differences in agar-based fluorescence across induction levels under blue-light transillumination (Doshi et al., 2023; Extended Data Figure 2b), consistent with our observations in MG1655hm pLac-*gfp* and pDawn-*mCherry* control strains.

In summary, fluorescence here served primarily to validate promoter behavior and to provide matched control circuits for the information-recording swarming strains that do not contain fluorescent reporters. We thus believe that additional agar-based fluorescence assays would be unlikely to substantially change the interpretation of the results or the core contribution of this work, namely the recording and decoding of input signals through macroscale colony morphology. We agree, however, that incorporating and quantifying fluorescent or chromoprotein reporters in agar-based contexts could be valuable in future work, particularly for enabling multiplexed or visually enhanced readouts alongside morphological changes.

MINOR POINTS

MAIN TEXT

-31, 65-67, 70-72) "Escherichia coli has not been engineered for robust control of swarming. [...] To date, most engineered bacterial patterning systems have relied on pre-patterned templates, quorum sensing, or swimming motility, approaches that frequently produce transient or microscale morphologies. [...] Here we leverage Escherichia coli-a genetically tractable chassis whose swarming and patterning behaviors have not been harnessed as biotechnological tools" This statement feels a bit exaggerated and minimizes the successes in the engineering of E. coli swarming and motility. Many milestones have been achieved, including coupling of swimming motility to light, microscopic patterns, macroscopic patterns with single and multiple strains. The author cites the appropriate works (references 9-17) but do not adequately acknowledge their contribution to the SynBio field. The same could be said for patterning, as E. coli has been largely used for countless patterns, at the micro and macro scale, leveraging different strategies.

We thank the reviewer for this thoughtful comment and agree that the original wording in the Abstract and Introduction may have understated the substantial progress made in engineering *E. coli* motility and patterning. In the revised Introduction, we now more explicitly acknowledge the diverse strategies and achievements in prior work for engineering spatial organization in microbial systems, including the use of optogenetics, quorum sensing, and swimming motility. We then position bacterial swarming, a distinct mode of collective surface motility, as a complementary strategy that builds on these advances. While swimming motility and patterning in *E. coli* have been extensively engineered across a range of spatial scales and approaches, the use of genetically programmable swarming as a biosensing and recording modality in *E. coli* remains comparatively underexplored. Accordingly, we have revised the relevant text to more accurately reflect this positioning.

-81-82) "regular, visually distinct, and resolved with low-cost, flatbed imaging" -> not clear what regular and visually distinct mean. Radially symmetric, homogeneous, smooth? Distinct from what? Maybe 'discernible to the naked eye' or 'distinguishable by the naked eye' are more appropriate? For low-cost flatbed imaging, refer to Methods, Swarming assays section.

We thank the reviewer for this helpful suggestion. We have revised the text to clarify what we mean by "an ideal baseline pattern," using more specific language ("consistent macroscopic morphology" and "readily distinguishable by eye"). Details of the flatbed imaging setup are provided in the revised Methods and Protocols, *Swarm-colony imaging*.

-82) "Screening MG1655 and genetic variants identified" -> missing subject. Either 'Screening, we identified...', or 'A screening allowed us to identify...' . Also, 'MG1655 strain and its genetic variants' might be more appropriate.

We thank the reviewer for this helpful comment. We have revised the sentence to improve the wording, now stating: "Screening the MG1655 strain and its genetic variants, we identified a hypermotile isolate (MG1655hm; CGSC #8237) that formed centimeter-scale, radially symmetric colonies on semi-solid agar with glucose."

-162) "SwinTransformer" -> move reference 32 next to the tool name and briefly clarify what it is (e.g. the hierarchical vision transformer SwinTransformer, or the Microsoft image manipulation tool SwinTransformer)

We thank the reviewer for this suggestion. In the revised Results, we have moved the citation immediately after "SwinTransformer" and briefly clarified that SwinTransformer is a hierarchical vision transformer architecture.

-169) "accuracies of 92% and 82%, top-2 accuracies of 96% and 88%, and AUCs of 0.99 and 0.89, respectively." -> while the conveyed message is clear, the reader might not be familiar with the definitions of accuracy, top-2 accuracy and AUC metrics. Consider adding a brief definition in the Methods or including a reference.

We thank the reviewer for this suggestion. In the revised Methods and Protocols (*Computational Analysis*), we have added brief definitions of accuracy and ROC–AUC where they first appear in the *Regression models* section, and of Top-2 accuracy where it first appears in the *Deep-learning models* section.

-232-233) "With pDawn, stronger gene expression could be due to combined effects of high-intensity blue light and the reported high strength of the λ pR promoter (or other elements of the pDawn system)". -> Have you tried to reduce blue light intensity, or to use periodic exposure (e.g. 1min ON - 3min OFF) instead of continuous illumination?

We thank the reviewer for this helpful question. **In response, we have added new experiments examining both low- and high-intensity blue-light conditions, alongside non-inducing dark and red-light controls (Revised Figure 6).** Across these conditions, increasing blue-light intensity produced graded changes in pDawn-*fliA* colony morphology, consistent with intensity-dependent control of gene expression. Under lower intensity blue light, pDawn-*fliA* formed intermediate phenotypes similar to those observed in pLac-*fliA* under high IPTG induction, whereas higher intensity blue light resulted in more compact colonies, as shown in the original manuscript. We agree that temporal modulation of illumination (*e.g.*, periodic exposure) represents an interesting direction for future work.

-276) "E. coli Mach1" -> add (ThermoFisher) if appropriate

We thank the reviewer for requesting this helpful detail. We have now specified Mach1's source as Thermo Fisher in the revised Methods and Protocols, *Bacterial strains, storage, and growth conditions*.

-388) "an incubator shelf" -> it is unclear how the light goes through the shelf. Is it a transparent shelf?

We thank the reviewer for requesting clarification. The incubator shelf was a standard metallic, perforated shelf. Illumination reached the Petri dishes through the shelf perforations, gaps between the shelf and incubator walls, and scattering from the metallic interior of the incubator. We have now clarified this in the revised Methods and Protocols, *pDawn swarming assays*.

-757-758) why did you use 10mM IPTG for some strains and 2mM for others? Clarify or include in supplementary?

We thank the reviewer for raising this point. In Figure 1C, we aimed to show representative high-induction phenotypes that were clearly distinguishable from the 0 mM IPTG condition. For the pLac-*fliA* and pLac-*rpoS* strains, 10 mM IPTG yielded a strong deviation from the baseline pattern. For the pLac-*lrp* strain, however, we observed that induction at 0.1, 0.5, and 2 mM IPTG produced similarly expanded swarm morphologies that fully covered the Petri dish, indicating that the phenotype had plateaued within this range. We therefore selected the 2 mM IPTG condition as a representative high-induction image for pLac-*lrp* in Figure 1C. In contrast, pLac-*cheZ* colonies did not exhibit discernible morphological changes relative to baseline across the IPTG concentrations tested, and the 2 mM condition was chosen as a representative example. We have clarified this strain-specific rationale directly in the revised Figure 1C legend.

-773) "false-color colony masks" -> it unclear what a colony mask represents here and how it is used for the analysis. Simply refer to Methods, section Colony segmentation, or to Supplementary Figure 2a

We thank the reviewer for this clarification request. In the revised legends for Figure 2B (pLac-*lrp*), Figure 3B (pLac-*fliA* and pLac-*rpoS*), and Appendix Figure S5B (pDawn), we have expanded the description to read "Representative false-color binary colony segmentation masks." In addition, following the sentence "All colony images and corresponding masks were used for analysis" in the Figure 2B and 3B legends, we have added the parenthetical note "(see Fig. EV2 and Methods and Protocols, *Colony segmentation* and *Colony feature extraction*)."

-781-782) "Representative masked polar-coordinate colony images at 0.05 mM IPTG are shown." -> specify in the caption what the dotted line and the two arrows represent in the lrp image (Fig 2d).

We appreciate the reviewer's suggestion. We have updated the Figure 2D and Figure 3C legends to explicitly define the visual annotations in the representative polar-coordinate colony images, now clarifying the directions along which pixel intensities and colony distances are sampled, as well as how values are aggregated and averaged.

-Are the features observed by the neural network model the same as the ones the authors used for the regression model?

We thank the reviewer for this question. The regression models operate on handcrafted morphological features extracted from preprocessed and segmented colony images, whereas the SwinTransformer models learn directly

from the colony images. To clarify this distinction, we have revised the Results to now explicitly state that the SwinTransformer models classify IPTG concentration directly from colony images, bypassing the handcrafted feature extraction required for the regression models.

-It would be better if you could measure the light intensity given in a commonly used unit, like W/m2 or mW/cm2.

We thank the reviewer for this suggestion. In the revised manuscript, we now report blue-light irradiance in standard radiometric units of mW cm^{-2} (Revised Figure 6B legend; Methods and Protocols *Liquid-culture induction assays* and *pDawn swarming assays*).

-The community would benefit most from your work if you provide all the constructs of this work on Addgene.

We appreciate and agree with the reviewer's suggestion. Addgene deposition of the constructs generated in this study is an ongoing effort in our lab. In the meantime, strains and plasmids are available from the authors upon request, as we now state in the revised Data Availability statement. If deposition is completed by the time of publication, we will update the Data Availability statement to include the corresponding Addgene accession numbers.

-It is nice that the methods are very detailed. However, in case space is an issue, standard methods like preparation of competent cells and electroporation could be removed. Most lab have those methods already established and unless we are mistaken, doing it slightly different should not prevent reproducibility of your results.

We appreciate the reviewer's suggestion. For now, we have retained step-by-step protocols in accordance with the journal's Structured Methods format. If requested by the editors, we can streamline this section by removing standard procedures (*e.g.*, electrocompetent cell preparation and electroporation), which follow widely used protocols and are not essential for reproducing the core swarming results.

-A suggestion for future work: You could test the light-inducible motility in an analog rather than a binary manner. This way, you could infer the light intensity from the colony size. Next, you could engineer additional strains that respond to green and red light. In your experiments, you would inoculate three colonies on the same plate, each one responsive to a different light color. This setup would allow you to determine the light composition and intensities based on the relative sizes of the three colonies.

We thank the reviewer for this thoughtful suggestion. **In response, we have now extended our optogenetic swarming assays to include both low- and high-intensity blue-light conditions (Revised Figure 6).** Our qualitative and quantitative results show that pDawn-*fliA* colonies exhibit graded, intensity-dependent changes in morphology, demonstrating that swarm phenotypes can record light input in an analog manner, consistent with our results for chemical inputs (IPTG).

We agree that expanding this framework to multiplexed sensing—such as engineering strains responsive to additional wavelengths (*e.g.*, green and red light)—represents an exciting direction for future work. Indeed, we highlight multiplexing as a future direction in the Discussion. **In addition, our added dual-strain deep-learning model (Revised Figure 5D) demonstrates a related capability for distinguishing inputs across multiple conditions and strain identities**, suggesting a path toward more advanced multiplexed sensing strategies.

-In an attempt to promote sustainable research practices, we encourage setting ultralow temperature freezers at -70{degree sign}C instead of -80{degree sign}C.

We thank the reviewer for this recommendation and appreciate the emphasis on sustainable research practices.

SUPPLEMENTARY MATERIAL

-Supplementary Figure 1b: reconsider if it is appropriate to confront the Total fluorescence quantified as area under each trajectory (which represents a response over time), instead of the endpoint fluorescence intensity. For example, a trajectory that spikes to 500a.u. at 9h and then falls to 0a.u. at 18h would have the highest Total fluorescence, but the lowest endpoint fluorescence. If the rationale of the experiment is to choose the ideal IPTG concentration to see differences in endpoint patterns, the endpoint fluorescence intensity should be used to quantify fold-change.

We thank the reviewer for this helpful suggestion and agree that endpoint fluorescence is the most appropriate metric for this experiment, as its purpose was to identify IPTG concentrations that yielded distinguishable endpoint swarm patterns. Accordingly, we have revised Appendix Figure S1B (previously Supplementary Figure 1b) such that the middle plot now shows endpoint fluorescence (final time point, ~18 h), and the bottom plot shows fold change in endpoint fluorescence relative to the 0 mM IPTG condition. We have also updated the corresponding descriptions in the revised Methods and Protocols (*Liquid-culture induction analysis*) and Results (*Programming E. coli swarming*) to reflect this change.

-Supplementary Figure 3: consider combining with Figure 3 in the main text. Show just one row of pLac-gfp control swarm pattern in Fig 3a, and add the corresponding colony mask in Fig 3b. This would avoid switching back and forth from Supplementary Information.

We thank the reviewer for this suggestion. In the revised manuscript, we have incorporated pLac-*gfp* control images directly into Figure 3A, reducing the need to switch between the main text and the Appendix. Specifically, revised Figure 3A now includes representative images of pLac-*gfp* swarm colonies grown in parallel with the pLac-*rpoS* experiments and corresponding to the quantitative analyses shown below, enabling direct visual comparison within the main figure.

-Supplementary Figure 7a: clarify whether 'Fluorescence trajectory' refer to the area under each trajectory as in Supplementary Figure 1b

We thank the reviewer for highlighting this distinction. The phrase “fluorescence trajectory” in the previous Supplementary Figure 7a legend may have caused confusion. We have therefore revised the legend and y-axis label (now Appendix Figure S5) to explicitly specify endpoint fluorescence, measured after a fixed total culture time of 24 h following transient blue-light illumination applied for the indicated durations. As noted above, we have likewise revised Appendix Figure S1 to present endpoint fluorescence, ensuring consistent quantification and terminology across figures.

Reviewer #2:

Summary

This paper explores how E. coli swarming patterns can be engineered and interpreted as information-encoding devices. The authors construct a hypermotile E. coli strain (MG1655hm) and introduce IPTG-inducible overexpression circuits for several motility-related genes (lrp, fliA, and rpoS). Varying IPTG levels produces distinct macroscopic patterns: a sharp, threshold-like (binary) response for lrp and gradual (analog) responses for fliA and rpoS. Using quantitative image features (colony area, Fourier spectra, eccentricity, etc.), the authors train classifiers and deep learning models to decode IPTG concentration from colony morphology. They further analyze temporal dynamics using a flatbed scanner for high-resolution time-lapse imaging, and they demonstrate modularity by replacing IPTG induction with a blue-light-inducible fliA circuit.

Overall, the paper presents an elegant integration of genetic engineering, quantitative imaging, and machine learning. The experimental setup is creative and the data are analyzed rigorously through well-documented pipelines. Figures are well organized (though a bit faint and hard to read on my computer), and the authors convincingly demonstrate that engineered swarming can record and decode environmental inputs.

However, several aspects could be better clarified and elaborated as detailed below.

We thank the reviewer for this thoughtful and positive summary of our work. We are encouraged that the reviewer recognizes the strengths of our integrated experimental and computational approach. As detailed below, we have clarified and improved several aspects of the manuscript in response to the reviewer's comments.

Comments

1. The authors emphasize that using E. coli provides a more genetically tractable chassis compared to Proteus mirabilis, featured in their previous Nat. Chem Bio paper (Doshi et al., 2023). However, the conceptual advance here is not fully articulated. The manuscript would benefit from explicitly explaining what new principles or capabilities emerge from this transition, e.g., the ability to tune pattern morphologies via specific regulatory genes (lrp, fliA, rpoS), to modulate swarming hierarchically, or to couple to optogenetic inputs. Also, the authors should note some related publications that demonstrate the use of phenotypic traits (growth/gene expression) to decode input signals, including: Duncker et al, BioRxiv 2025, Engineering microbial consortia for distributed signal processing

We thank the reviewer for raising these important points and agree that the conceptual advances of this work can be more clearly articulated. In the revised manuscript, we have expanded the Introduction to now more explicitly highlight the capabilities enabled by using a genetically tractable chassis with a well-developed synthetic biology toolkit. These include modular genetic control, compatibility with multiple input modalities (chemical and optogenetic), and support for both binary and analog modes of information recording. We also emphasize our expanded use of flexible and scalable computational pipelines for decoding swarm phenotypes across strains, input conditions, and in both space and time.

Additionally, we have expanded the Introduction to better situate our *E. coli* swarming platform within the broader landscape of biological information storage. Specifically, we have extended our discussion beyond DNA-based recorders to now include phenotypic recording systems (*e.g.*, gene-expression dynamics and spatial patterns), referencing relevant studies including Duncker et al. (2025). Within this context, we now position swarm morphology as an alternative phenotypic readout that captures environmental inputs across space and time in large populations, thereby enabling intuitive, macroscale outputs. Together, these revisions more clearly define the contribution of this work as establishing a modular, extensible platform for recording and decoding environmental information in swarming *E. coli*.

2. The decoding accuracies (AUC up to 1.0) appear impressive but possibly optimistic given modest sample sizes (n = 9-35 colonies per condition). Although data augmentation and held-out testing are described, the paper should discuss potential overfitting and model generalization across biological replicates and experimental days. Demonstrating decoding of an independent dataset or cross-day validation would substantially increase confidence in the robustness of the classifiers.

We thank the reviewer for raising these important points regarding sample size, potential overfitting, and model generalization. The *n* = 9–35 colonies per condition reported in feature-based analyses of MG1655hm pLac-*fliA*, pLac-*rpoS*, and their pLac-*gfp* controls correspond to the biological replicates plotted in Figures 3 and EV3. The regression models were fitted to and evaluated on these feature datasets (Appendix Table S4). In contrast, the deep-learning models were trained on expanded image datasets that incorporated additional colonies beyond those used in the feature-based analyses, as such models benefit from larger dataset sizes (Appendix Table S6). As now clarified in the revised Methods and Protocols (*Computational Analysis*), all image datasets used in all analyses were collected across multiple independent experiments conducted over several months under consistent protocols. Accordingly, model training, validation, and test dataset splits contained shuffled samples spanning multiple experimental days, providing inherent biological variability.

In the revised Methods and Protocols we also detail the computational steps taken to mitigate potential overfitting and to support model generalization. For deep-learning models, we used stratified data splitting, pretraining, fine-tuning, data augmentation, early stopping, and held-out test sets. For feature-based regression models, we used low-complexity models with a limited number of predefined image features and evaluated performance on held-out test sets, reducing the risk of overfitting to individual replicates. We agree that evaluation on a fully independent dataset or explicit cross-day validation would further strengthen confidence in model robustness. While our current datasets already span multiple experimental days, we view more formal cross-day validation as a valuable direction for future work and thank the reviewer for this suggestion.

3. With the rationale of using temporal features to better support decoding, it would have been nice to see how expansion rate or textural uniformity improves AUC of -rpos, given the caveat that there is little room for improvement of -lrp and -fliA since the prediction performance from spatial features alone is quite high already.

We thank the reviewer for this suggestion. Temporal features such as expansion rate and colony uniformity indeed provide informative descriptors during colony growth and are particularly useful for enabling earlier decoding of IPTG inputs. In our current framework, however, the regression models were designed to operate on endpoint spatial features derived from 24-h colony images, whereas the temporal measurements were obtained from separate time-lapse experiments and analyzed through a distinct pipeline. We thus did not directly evaluate whether incorporating temporal features would improve AUC within the current regression framework. By the 24-h endpoint, spatial colony features already support strong decoding performance (*e.g.*, AUC ~0.96 for pLac-*rpoS* in the regression analysis) Consistent with this, a newly added unified deep-learning model trained on pooled pLac-*fliA* and pLac-*rpoS* colony images achieved 93% accuracy, 98% Top-2 accuracy, and a macro-averaged AUC of 0.9986 across the nine-class classification task. These high test metrics indicate that endpoint morphologies already contain sufficient structured information for accurate classification. We agree with the reviewer that integrating temporal and spatial features within a unified framework will be an important future step, particularly in more complex or multiplexed recording scenarios where additional information may significantly improve decoding performance.

4. The pDawn-fliA experiments successfully illustrate modularity in input sensing but remain preliminary. The demonstration is limited to a binary comparison (red vs. blue light) without a corresponding decoding analysis or analog gradient. As written, this section feels disconnected from the rest of the paper. Expanding this part to include a quantifiable light-dose response or prediction of illumination time from colony patterns would make it more cohesive.

We thank the reviewer for this insightful comment. In response, we have expanded the experiments, analysis, and Discussion surrounding pDawn to now better integrate it with the rest of the manuscript. **First, in the pDawn swarming assays, we now include graded blue-light conditions (low and high irradiance) alongside two non-inducing controls (dark and red light; Revised Figure 6).** pDawn-*fliA* swarm colonies exhibited visible and quantifiable dose-dependent morphological responses to increasing blue-light input. We now describe these changes using multiple colony features—minimum radius, global intensity coefficient of variation (CV), and mean circumferential intensity CV—whereas we previously presented only minimum radius.

Second, we have now clarified the connection between the optogenetic and chemical systems. Specifically, we better motivate our choice of *fliA* within pDawn; draw clearer parallels between the response patterns of pLac-*fliA* and pDawn-*fliA*; and emphasize that we analyzed the pDawn datasets using the same endpoint image-processing and feature-extraction pipeline as the pLac datasets, highlighting the generalizability of the computational framework.

Finally, we have revised the Discussion to better position the pDawn system within the overall framework and expand on its broader implications. We highlight that the ability of swarm colonies to record both chemical and optical information demonstrates the platform's modularity across these distinct inputs, and thus its potential to interface with diverse signals of environmental, clinical, and materials relevance. We now also emphasize that optogenetic systems have been widely applied to control bacterial behaviors with high spatial and temporal precision in applications ranging from microbial therapeutics to engineered living materials, and that our results suggest collective swarming behaviors could be incorporated into such platforms. Together, these additions strengthen the cohesion of the pDawn experiments with the rest of the study and demonstrate that the platform can record and quantify both chemical and optical inputs in an analog manner.

5. The decoding tasks were trained separately for each engineered strain. It would strengthen the generality of the approach to test whether a single model can classify both IPTG concentration and strain identity. This would demonstrate that the learned morphological features reflect underlying biological structure rather than dataset-specific cues-an important step toward scaling to multiplexed sensing applications.

We thank the reviewer for this important suggestion. **In response, we implemented a new unified deep-learning model to jointly decode both strain identity and IPTG concentration from pooled image datasets, addressing the question of generality and multiplexed sensing (Revised Figure 4B,D; Appendix Figure S4 and Table S7).** Specifically, we trained a nine-class SwinTransformer classification model on combined pLac-*fliA* and pLac-*rpoS* colony images, where each class corresponds to a unique strain–IPTG condition. Despite the increased complexity of jointly distinguishing gene- and input-dependent morphological features, the unified model achieved high performance on held-out test data (93% accuracy, 98% Top-2 accuracy, and 0.9986 macro-averaged AUC). Notably, this performance exceeded that of the individual strain-specific models, demonstrating that multiplexed decoding can be achieved without sacrificing accuracy.

To assess whether this performance reflects underlying biological structure rather than dataset-specific cues, we compared this unified model to a matched nine-class pLac-*gfp* control model, which exhibited substantially lower performance and limited class-specific separability. Together, these results demonstrate that a single model can successfully decode both strain identity and inducer concentration from colony morphology, and that the learned features reflect biologically structured, dose-dependent pattern formation. This supports the scalability of our approach toward multiplexed sensing applications.

6. I did not understand why having a digital-like response for the lrp strain, wherein the patterns seem similar till a certain threshold and similar after that threshold is leading to a better classification performance. The analog behavior(fliA and rpoS strains), wherein the change is more gradual and conditions can be seemingly distinguished by the naked eye has a lower performance. This is reflected in both the machine learning and deep learning pipelines.

We thank the reviewer for raising this point. The difference in decoding performance arises primarily from the classification task structure rather than from any inconsistency in the underlying biology. The pLac-*lrp* strain exhibits a threshold-like phenotypic response that produces two distinct and well-separated colony patterns, resulting in a binary classification task. In contrast, the pLac-*fliA* and pLac-*rpoS* strains record IPTG in an analog-like manner, producing gradually varying and more similar colony patterns across five IPTG concentrations. This greater similarity in colony patterns across a larger number of IPTG classes makes perfect separation inherently more challenging than in the binary-like *lrp* case, leading to slightly lower—but still robust—performance for both the multiclass regression and deep-learning models. We have added a brief clarification in the Results to explain this difference in decoding task complexity.

7. Figures 2 and 3 use distinct sets of features for binary and analog strains. While the Supplementary Tables list these, a short statement in the main text explaining that feature sets were optimized separately for each strain would reduce confusion.

We thank the reviewer for this helpful suggestion. We have now explicitly clarified that feature sets were optimized independently for each engineered strain. In the revised Results, we note that the optimal model for each strain leveraged a unique combination of statistically significant features. In the revised Methods and Protocols (*Regression models*), we specify that feature selection and regression model fitting were performed independently for each strain to account for strain-specific IPTG–phenotype relationships. The specific feature combinations contributing most strongly to high decoding performances are reported in Figure EV3 (previously Supplementary Figure 5) and Appendix Table S5 (previously Supplementary Table 5). Figures 2 and 3 present additional representative morphological features to illustrate the breadth of IPTG-dependent phenotypic trends for each strain.

8. Panels (c-h) of Figure 4, focusing on spatial and temporal feature analysis, are conceptually separate from the machine- and deep-learning components in panels (a-b). The paper would flow more clearly if these were placed in a separate figure or moved to the Supplementary section.

We thank the reviewer for this thoughtful suggestion. In response, we have revised the figure organization to improve conceptual clarity and flow, breaking the original Figure 4 into three revised figures (Figures 4–6) that clearly separate classification, temporal, and spatial analyses. Revised Figure 4 now focuses exclusively on the classification results from the regression and deep-learning models (with extended results in Figures EV3–EV4 and Appendix Figure S4). Revised Figure 5 presents the time-lapse feature analysis. Revised Figure 6 presents an expanded spatial analysis of the pDawn swarming assays.

9. For their inference pipeline, the authors also used a deep-learning based approach to bypass human-guided extraction of features. I very much like the approach as I in principle agree with the rationale - the model should be able to learn relevant features (whether or not they're human interpretable) that are important for prediction. Overall, it appears that they achieved comparable performances. I think it's valuable to discuss a bit more the pros and cons of each approach.

We thank the reviewer for this thoughtful comment. In response, we have expanded the Discussion to more explicitly compare the advantages and limitations of the feature-based regression and deep-learning approaches. Specifically, we now highlight that regression models provide interpretable links between individual morphological features and predictive performance, offering insight into how regulatory perturbations shape swarm phenotypes and potentially informing future circuit design. In contrast, deep-learning models learn predictive representations directly from images without predefined feature extraction, enabling greater flexibility and potentially improved robustness to variation in imaging conditions. Such models may also simplify deployment in automated or user-facing imaging workflows (*e.g.*, mobile platforms), albeit at the cost of increased data and computational requirements. These distinct computational approaches involve trade-offs between interpretability, flexibility, and scalability, and can therefore serve complementary roles in biological information-decoding systems.

10. I wish they had deposited the data and codes to a public repository. Regardless, I'd encourage the authors to do so at least when the paper is accepted for publication.

We thank the reviewer for this important suggestion. In response, we have deposited all datasets and code generated in this study in publicly accessible repositories, as described in the revised Data Availability section. Raw full-resolution colony endpoint and time-lapse image datasets are deposited on Zenodo. Plate-reader data, processed datasets, model outputs, and analysis code are maintained in a GitHub repository (https://github.com/daninolab/ecoli-swarming-recording) and have also been archived on Zenodo. The code includes scripts for plate-reader data processing, colony preprocessing and segmentation, spatial and temporal feature extraction and analysis, and classification models.

Access links to these resources are provided below for the editor and reviewers during the review process, and these resources will be made publicly available upon publication.

Endpoint image datasets:
https://zenodo.org/records/19612563?preview=1&token=eyJhbGciOiJIUzUxMiJ9.eyJpZCI6ImZkZWExMmJiLWUyZjItNGU3Zi04ZjdmLTIwYzI3N2U5OGIyMCIsImRhdGEiOnt9LCJyYW5kb20iOiJjMWMwMWQ0ZTc5MTE0NDc2OTVkYTEyY2IyNjk1ZTY5MCJ9.8M31Id368IqIyqba0txSUBJ2AtwChEgu3q87viCTCp5Q3cVFNTBcrSw6HlIEUHfaH2wpkqgHeJ6AMNsxjI34Og

Time-lapse image datasets:
https://zenodo.org/records/19615229?preview=1&token=eyJhbGciOiJIUzUxMiJ9.eyJpZCI6IjlmZjVkOGRlLTMxYWEtNDYzMy1iYTM1LWJlZjM2YWZkNzk5ZiIsImRhdGEiOnt9LCJyYW5kb20iOiJmYTY2YThmZGFlZjMyZjAwOWU1NmQ1NWYyOTczNzYxNiJ9.KytVLw5ZhmypITVlUa9u5Vb1CNbov0u95r6dbaih_7R5K8X69Yo8HIxRqeJtnes7TN1eNEDfn5zwxR8IHqITPQ

Code and processed data archive:
https://zenodo.org/records/19857501?preview=1&token=eyJhbGciOiJIUzUxMiJ9.eyJpZCI6IjY1YzY0NzIyLTMyNjAtNGU0OS1hZjM4LTg1MzY3ODZmYzY2OSIsImRhdGEiOnt9LCJyYW5kb20iOiJhOTc5NzY4MGExOGY0NDBhZTMyNzA2Yjc3NzI0ZTU4NyJ9.mVyFfWEVlyMYwDix2fXV1awX0s1vU9Otw-7-dncH4czUR-6hjitwNIIHEAGjShGgHrdLh0RoB59c5xb7dvMnXQ

Other minor suggestions.
Line 83: Provide a citation or note for the origin or construction of the hypermotile E. coli strain.

We thank the reviewer for this suggestion. In the revised Methods and Protocols (*Bacterial strains, storage, and growth conditions*), we have clarified the origin of the hypermotile strain (MG1655hm) used in this study. Specifically, we note that MG1655hm corresponds to "MG1655(motile)" from the Coli Genetic Stock Center (CGSC; strain #8237), an isolate derived from MG1655 (CGSC #6300) that was selected and verified for motility. We have also added citations describing the reported genetic basis for increased motility in this strain (Barker et al., 2004; Pleška et al., 2021). Additionally, we have clarified the strain identifier when first introducing MG1655hm in the Results section ("MG1655hm; CGSC #8237").

Line 101: Clarify how fliA overexpression, which enhances flagellar gene expression, mechanistically leads to reduced swarming. Are swimming and swarming oppositely regulated in this context?

We thank the reviewer for this insightful question. Although *fliA* (the flagellum-specific sigma factor σ^{28}) promotes expression of late flagellar genes, swarming on semi-solid surfaces is a coordinated multicellular behavior that depends not only on flagellar gene expression but also on balanced regulation across the flagellar transcriptional hierarchy. Overexpression of *fliA* may therefore drive strong activation of Class III flagellar assembly and chemotaxis genes independently of maintaining upstream regulatory coordination. Such excessive activation can disrupt the regulatory balance of the motility program or impose energetic costs associated with flagellar biosynthesis and rotation (Fitzgerald et al., 2014), which may ultimately limit swarm expansion despite increased expression of flagellar components. Thus, swimming and swarming are not oppositely regulated per se; rather, efficient swarming requires coordinated regulation of the motility network beyond simply increasing flagellar gene expression. We now clarify this interpretation in the revised Results section alongside Figure 1B,C.

Line 110: Include representative replicate colony images (e.g., in Supplementary Information) to show the reproducibility of the observed morphologies.

We thank the reviewer for this suggestion. **In response, we have added a new Expanded View figure (Revised Figure EV1) containing representative replicate colony images to demonstrate the reproducibility of the observed morphologies.** For each engineered strain, three independent biological replicates (n = 3) are shown under a representative induction condition (IPTG or blue light, as indicated), illustrating qualitative consistency across experiments while keeping the Expanded View concise. Additional replicate images are available in the publicly released datasets described in the Data Availability section.

Line 126: Explain why different IPTG concentration ranges were used for fliA and rpoS experiments-was this based on empirical optimization or sensitivity differences?

We thank the reviewer for raising this point. In the revised Results section describing the analog-recording strains, we now note that we selected distinct IPTG concentrations empirically through strain-specific screening to capture the IPTG window over which pLac-*fliA* and pLac-*rpoS* exhibited measurable morphological changes.

For clarity and consistency, we now also indicate this empirical selection in the legends of revised Figure 2A, Figure 3A, and Appendix Figure S2.

Line 224: Add a brief rationale for choosing fliA as the gene for optogenetic control.

We thank the reviewer for this suggestion. In the IPTG-inducible system, *fliA* overexpression produced a strong, graded morphological response and enabled accurate decoding of inducer levels from colony images. These results motivated our selection of *fliA* to test whether the system could similarly record varying levels of blue light. We have added this brief rationale in the revised Results section when introducing the pDawn-*fliA* experiments.

Line 244: Move speculative discussion about future chromoprotein applications to the Discussion section.

We thank the reviewer for this suggestion. As noted in our responses to Reviewers 1 and 3, we have removed the chromoprotein figure and its associated Results text from the revised manuscript. We agree, however, that the broader concept of multiplexed recording and alternative readouts is appropriate for the Discussion. We have thus retained the brief forward-looking statement there that contextualizes how future work could integrate additional colorimetric or spectral outputs into swarm-based information recording.

Reviewer #3:

This work introduces a synthetic biology platform that employs engineered swarming motility of E. coli to spatially record chemical and optical inputs as visual-scale colony morphologies. By modulating key motility regulators, the authors establish a 'visual language' of patterns that they aim to decode using a computational pipeline integrating texture analysis and deep learning.

I like, in theory, the idea of modulating the swarm behavior of bacteria using genetic switches and dials. I like the promise of the work, additionally. I have no considerable issues with the design, performance or portrayal of the work.

We thank the reviewer for this thoughtful and encouraging assessment of our work. We are pleased that the reviewer appreciates the conceptual framework of using engineered swarming behavior to record environmental inputs, as well as the overall design and performance of the platform. As detailed below, we have addressed the reviewer's comments and suggestions, which have helped us further clarify and strengthen the manuscript.

Specific points:
In fig3, images of the GFP control should be included in the main figure, especially given that the quantitative descriptions in the plot below includes them.

We thank the reviewer for this comment. In the revised manuscript, we have incorporated pLac-*gfp* control images directly into Figure 3A, now presenting the visual controls that correspond to the quantitative analyses shown below. Following Reviewer 2's suggestion, we include a single row of pLac-*gfp* control colonies grown in parallel with the pLac-*rpoS* experiments in revised Figure 3A to avoid redundancy. Representative images of pLac-*gfp* controls grown in parallel with the pLac-*fliA* experiments are shown in revised Appendix Figure S2. The pLac-*gfp* control colonies are qualitatively and quantitatively similar across the IPTG ranges associated with both pLac-*fliA* and pLac-*rpoS* experiments.

General comments:
This work establishes a fundamental ability to engineer macroscopic swarm patterns, but it essentially operates as a technical proof-of-concept. To demonstrate true utility, the authors need to show the system working in a relevant application beyond simple induction assays (such as metal sensing as authors exemplify in Discussion section).

We thank the reviewer for this important comment. We agree that demonstrating the broader utility of the platform is critical. Importantly, the optogenetic (pDawn) experiments extend beyond simple chemical induction assays that use standard laboratory reagents. Demonstrating that swarm morphologies can record blue-light inputs establishes compatibility with an existing and application-relevant class of biological control systems, rather than blue light functioning solely as a synthetic or abstract input. We have revised the Discussion to emphasize that optogenetic systems have been applied to control bacterial behaviors with high spatial and temporal precision in contexts ranging from microbial therapeutics to engineered living materials, and that our results further suggest that swarming-based spatial recording could be incorporated into such platforms as an additional, macroscale readout of cellular responses.

More broadly, the goal of this study was to establish a generalizable, colony-scale information-recording framework in swarming *E. coli* that supports multiple modes of information processing and interoperability across distinct input types, enabling future integration with diverse sensing applications. In the revised manuscript, we further strengthen the applicability of this framework by demonstrating graded (analog) recording of not only

chemical but also optical inputs; spatially and temporally resolved recording of localized IPTG perturbations; and scalable, multiplexed decoding via a unified multi-class model.

We have also expanded the Discussion to emphasize how this modular architecture can interface with existing, well-characterized *E. coli* sensor circuits across diverse domains, including environmental, clinical, and materials contexts, without requiring re-engineering of the swarm-patterning system. Together, these expanded results and context demonstrate that the platform provides a generalizable and extensible strategy for translating diverse, application-relevant inputs into spatially resolved, interpretable outputs.

There is a notable inconsistency in the depth of analysis between the chemical and optical recording sections. While the chemical recording utilizes robust, multi-dimensional feature extraction, the optical recording is left to primarily rely on 'minimum colony radius.'
This is understandably due to the difference in the nature of the promoters. However, this kind of biological 'quirk' is also what elevates the need of demonstration of applicability and scalability over the demonstration of concept. This reduction to a single metric makes the optical system more susceptible to experimental artifacts and less distinct than the high-dimensional 'visual language' established earlier in the paper. A consistent application of the computational pipeline to the optical data is needed to validate the system's robustness.
The work could have benefited from experimenting with varying light intensities or periodic duty cycles. Without tunability, I fail to perceive the utility of light based sensors - a very crude parallel could be exposure to anything that inhibits swarming thus eliciting a binary response.

We thank the reviewer for this insightful comment. In response, we have expanded the experiments, analysis, and discussion surrounding pDawn to better integrate it with the rest of the manuscript and to address concerns regarding consistency, feature dimensionality, applicability, scalability, and tunability. **First, in the pDawn swarming assays, we now include graded blue-light conditions (low and high irradiance) alongside two non-inducing controls (dark and red light; Revised Figure 6).** pDawn-*fliA* swarm colonies exhibited visible and quantifiable dose-dependent morphological responses to increasing blue-light irradiance, demonstrating that the optogenetic system supports analog recording of input strength rather than solely a binary response.

Second, we no longer rely on a single metric to characterize optical recording. We applied the same computational pipeline in the original manuscript and now report multiple colony features—minimum radius, global intensity coefficient of variation (CV), and mean circumferential intensity CV—demonstrating that this computational approach generalizes across both pLac and pDawn datasets. These features varied systematically with illumination intensity, indicating that optical inputs are recorded in a multi-dimensional morphological space and are less susceptible to any single-feature artifact.

Finally, we have revised the Discussion to better position the pDawn system within the overall framework and expand on its broader implications. We highlight that the ability of swarm colonies to record both chemical and optical inputs demonstrates the platform's modularity across these distinct inputs, and thus its potential to interface with diverse signals of environmental, clinical, and materials relevance. We also note that optogenetic systems have been widely used to control bacterial behaviors with high spatial and temporal precision in applications ranging from microbial therapeutics to engineered living materials, and that our results suggest collective swarming behaviors could be incorporated into such platforms. Together, these additions strengthen the cohesion of the pDawn experiments with the rest of the study and demonstrate that the optical recording system supports multi-feature, tunable, and scalable recording of input signals, consistent with the high-dimensional "visual language" established in the chemical recording experiments.

The inclusion of the chromoprotein data (pg 9, supp. fig 8) feels disconnected from the manuscript's primary focus on morphological recording. The sudden introduction of color as an output channel, even though described by the authors themselves as 'preliminary' and 'briefly explored', lacks the depth of characterization seen in the swarming experiments. Because these colored strains are not integrated into the computational decoding pipeline, this section appears as an add-on that dilutes the cohesion of the main story rather than enhancing the proof-of-concept.

We thank the reviewer for this helpful comment. We agree that the chromoprotein data were not sufficiently integrated with the manuscript's primary focus on swarm-based information recording and computational decoding. To maintain the coherence of the main narrative, we have removed this section from the revised Results and have deleted the corresponding supplementary figure. The broader concept of alternative visual outputs is retained only briefly in the Discussion as a potential future direction.

Swarming itself offers unique advantages: it produces macroscale readouts on timescales comparable to fluorescent reporters" - in the discussion section sounds like an overstatement. Swarming, technically, is a growth-dependent process resulting in a macroscopic endpoint (typically 24 hours), (even if one would restrict the observations to colony expansion, it is still a long process), whereas fluorescent reporters can potentially enable real-time dynamic monitoring (for e.g. doi = 10.1038/s41592-022-01597-x). The limited 'early detection' data provided for IPTG induction also, is insufficient to claim temporal equivalence with fluorescent methods.

We thank the reviewer for this important clarification. We agree that the previous statement comparing swarming timescales to fluorescent reporters could be interpreted as overstating temporal equivalence. We have therefore removed this comparison. We now provide a more balanced comparison of swarm-based sensing with traditional sensor modalities, explicitly noting the slower timescale of swarming relative to real-time measurements while highlighting its complementary capability to integrate environmental inputs over time and generate stable, macroscale readouts.

Concluding, the work should include demonstration of applicability beyond the proof of concept, such as an environmental sensing task, and must optimize the optical circuit to achieve the claimed analog recording capability, which currently appears limited by leakiness and a reliance on only binary inputs.

We thank the reviewer for this summary comment. As detailed above, we have clarified and strengthened the applicability of the platform in several ways. In the expanded Discussion, we now clarify that the optogenetic (pDawn) system represents an application-relevant input modality, and we emphasize how the swarming platform can interface with existing, well-characterized *E. coli* sensor circuits across environmental, clinical, and materials contexts.

We have also addressed concerns regarding optical recording by introducing graded blue-light conditions and demonstrating analog, multi-feature responses across increasing illumination intensities, rather than a binary single-feature response. We performed these analyses using the same computational pipeline as the pLac datasets, demonstrating consistency and generalizability across strains and input modalities.

Together with the addition of spatiotemporally resolved recording via localized IPTG perturbations and scalable, multiplexed decoding via a unified nine-class classification model, these revisions demonstrate that the platform supports tunable, multi-dimensional, and extensible information recording and decoding. We therefore believe the revised manuscript addresses the reviewer's concerns regarding applicability and analog recording capability. All in all, these results open avenues for future work exploring the integration of the platform with diverse sensing applications and real-world contexts.

28th May 2026

Manuscript Number: MSB-2025-13399R
Title: Engineered E. coli swarming for binary and analog input recording

Dear Dr. Danino,

Thank you for the submission of your revised manuscript to Molecular Systems Biology. We have now received the enclosed reports from the referees that were asked to re-assess it. As you will see the reviewers are now globally supportive and I am pleased to inform you that we will be able to accept your manuscript pending the following final amendments:

1) In the author checklist, it is not necessary to indicate that cell lines have been used if only bacterial strains have been used, as this is indicated in the microbes section of the checklist.
2) In the main manuscript file, please include keywords to max. 5.
3) Please format the Data availability section according to the example below, ensuring that all private data and code are now publicly accessible:
"The datasets and computer code produced in this study are available in the following databases:
- Chip-Seq data: Gene Expression Omnibus GSE46748 (https://www.ncbi.nlm.nih.gov/geo/query/acc.cgi?acc=GSE46748)
- Modeling computer scripts: GitHub (https://github.com/SysBioChalmers/GECKO/releases/tag/v1.0)
- [data type]: [full name of the resource] [accession number/identifier] ([doi or URL or identifiers.org/DATABASE:ACCESSION])"
4) Please include a README file on Github/Zenodo with practical use instructions for potential future users of your code.
5) Employment in a biotech company such as Google DeepMind should be listed under "Disclosure and competing interests statement". We updated our journal's competing interests policy in January 2022 and request authors to consider both actual and perceived competing interests. Please review the policy https://link.springer.com/partners/embo-press/editorial-policies#Competing%20interest%20disclosures and update your competing interests if necessary.
6) Please correct the reference citation in the reference list to be alphabetical (not numerical). Where there are more than 10 authors on a paper, only the first 10 should be listed, followed by "et al.". DOIs should only be used for preprints and datasets that have not been published yet. Please check "Author Guidelines" for more information:
https://link.springer.com/journal/44320/submission-guidelines#cms-Reference-guidelines
7) In the Methods, please take care of the following:
- The Materials and Methods section should be renamed to "Methods".
- Please remove the "Reagents and Tools table" title/placeholder from the main manuscript.
8) Please place individual sections of the manuscript in the following order: Title page - Abstract & Keywords - Introduction - Results - Discussion - Methods - Data Availability - Acknowledgements - Disclosure and Competing Interests Statement - References - Figure Legends - (Main Tables with legends if applicable) - Expanded View Figure Legends.
9) For the figures and figure legends, please take care of the following:
- Please remove all figures from main manuscript file and leave only main figure legends placed after the references.
- Please indicate the statistical test used for data analysis in the legends of figures 2C,D,E; 3C-F; 5B; EV-3B.
10) Please upload Table EV1-EV2 as one .xsl file per table and rename them to Dataset EV1-X. The legend/notes provided in the README file should be added to the corresponding file in a separate tab. Please update their callouts in main manuscript text. Please remove the legends for these files from the main manuscript.
11) Please zip each individual Movie file together with its respective legend provided in a text file. Please remove the legends for these files from the main manuscript.
12) In the Appendix file, please remove the author list and affiliations from the title page.
13) As part of the EMBO Publications transparent editorial process initiative (see our policy here:
https://www.embopress.org/transparent-process#Review_Process), Molecular Systems Biology will publish online a Peer Review File (PRF) to accompany accepted manuscripts. This file will be published in conjunction with your paper and will include the anonymous referee reports, your point-by-point response and all pertinent correspondence relating to the manuscript. Let us know whether you agree with the publication of the PRF and as here, if you want to remove or not any figures from it prior to publication. Please note that the Authors checklist will be published at the end of the PRF.
14) After your paper is published, we may promote it on social media. If you have any handles or hashtags for Bluesky you would like included, please let us know.
15) Please provide a point-by-point letter INCLUDING my comments and your detailed responses (as Word file).

I look forward to reading a new revised version of your manuscript as soon as possible.

Yours sincerely,

Poonam Bheda, PhD
Scientific Editor

Reviewer #1:

The authors addressed all our comments satisfactorily. Consequently, the manuscript has significantly improved, and we recommend its publication.

Reviewer #2:

The authors have fully addressed my comments on the original version. The revised version has been substantially improved.

Reviewer #3:

I am satisfied with the resolution on all of my comments except for the one major one -- experimental demostration of the proof-of-concept as a functional sensor for e.g..
In my revised opinion, given the new form of the manuscript, I am willing to accept the lack of aforementioned demonstration to be scope-adjacent for this work and thus dont reckon it as the only reason why this work should not be published. I therefore recommend this work for publication.

Point-by-point Responses to the Editor and Reviewers
Manuscript Number: MSB-2025-13399R
Title: *Engineered E. coli swarming for binary and analog input recording*

We thank Dr. Bheda and the reviewers for their positive assessment of our revised manuscript and for the opportunity to address the remaining editorial requests. We have addressed each comment below.

1) In the author checklist, it is not necessary to indicate that cell lines have been used if only bacterial strains have been used, as this is indicated in the microbes section of the checklist.

We have updated the author checklist by selecting “Not Applicable” for cell lines.

2) In the main manuscript file, please include keywords to max. 5.

We have added five keywords to the manuscript.

3) Please format the Data availability section according to the example below, ensuring that all private data and code are now publicly accessible:
"The datasets and computer code produced in this study are available in the following databases:
- Chip-Seq data: Gene Expression Omnibus GSE46748 (https://www.ncbi.nlm.nih.gov/geo/query/acc.cgi?acc=GSE46748)
- Modeling computer scripts: GitHub (https://github.com/SysBioChalmers/GECKO/releases/tag/v1.0)
- [data type]: [full name of the resource] [accession number/identifier] ([doi or URL or identifiers.org/DATABASE:ACCESSION])"

We have accordingly reformatted the Data Availability section and have made all private data and code publicly accessible.

4) Please include a README file on Github/Zenodo with practical use instructions for potential future users of your code.

We have updated the README file on GitHub (which is also archived at Zenodo) with a “Usage” section that states practical instructions for accessing and using the code and datasets.

5) Employment in a biotech company such as Google DeepMind should be listed under "Disclosure and competing interests statement". We updated our journal's competing interests policy in January 2022 and request authors to consider both actual and perceived competing interests. Please review the policy https://link.springer.com/partners/embo-press/editorial-policies#Competing%20interest%20disclosures and update your competing interests if necessary.

We have updated the Disclosure and Competing Interests Statement to indicate that S.S. and T.T. are employees of Google DeepMind.

6) Please correct the reference citation in the reference list to be alphabetical (not numerical). Where there are more than 10 authors on a paper, only the first 10 should be listed, followed by "et al.". DOIs should only be used for preprints and datasets that have not been published yet. Please check "Author Guidelines" for more information: https://link.springer.com/journal/44320/submission-guidelines#cms-Reference-guidelines

We have reformatted the references according to the journal guidelines, including citation style, reference ordering, author-list formatting, and DOI usage.

7) In the Methods, please take care of the following:
- The Materials and Methods section should be renamed to "Methods".
- Please remove the "Reagents and Tools table" title/placeholder from the main manuscript.

We have renamed the section to "Methods" and removed the "Reagents and Tools table" title/placeholder from the manuscript.

8) Please place individual sections of the manuscript in the following order: Title page - Abstract & Keywords - Introduction - Results - Discussion - Methods - Data Availability - Acknowledgements - Disclosure and Competing Interests Statement - References - Figure Legends - (Main Tables with legends if applicable) - Expanded View Figure Legends.

We have reordered all manuscript sections accordingly.

9) For the figures and figure legends, please take care of the following:
- Please remove all figures from main manuscript file and leave only main figure legends placed after the references.
- Please indicate the statistical test used for data analysis in the legends of figures 2C,D,E; 3C-F; 5B; EV-3B.

We have removed all figures from the main manuscript file and retained only the figure legends after the references. We have also updated the specified figure legends to indicate the statistical tests used for data analysis.

10) Please upload Table EV1-EV2 as one .xsl file per table and rename them to Dataset EV1-X. The legend/notes provided in the README file should be added to the corresponding file in a separate tab. Please update their callouts in main manuscript text. Please remove the legends for these files from the main manuscript.

We have renamed Tables EV1–EV2 as Datasets EV1–EV2, uploaded them as separate Excel files, added the corresponding legends to separate tabs within each file, updated the manuscript callouts, and removed the associated legends from the manuscript.

11) Please zip each individual Movie file together with its respective legend provided in a text file. Please remove the legends for these files from the main manuscript.

We have uploaded each movie together with its corresponding legend as a text file in a zipped archive and removed the movie legends from the manuscript.

12) In the Appendix file, please remove the author list and affiliations from the title page.

We have removed the author list and affiliations from the Appendix title page.

13) As part of the EMBO Publications transparent editorial process initiative (see our policy here: https://www.embopress.org/transparent-process#Review_Process), Molecular Systems Biology will publish online a Peer Review File (PRF) to accompany accepted manuscripts. This file will be published in conjunction with your paper and will include the anonymous referee reports, your point-by-point response and all pertinent correspondence relating to the manuscript. Let us know whether you agree with the publication of the PRF and as here, if you want to remove or not any figures from it prior to publication. Please note that the Authors checklist will be published at the end of the PRF.

We agree to publication of the Peer Review File and do not request removal of any figures.

14) After your paper is published, we may promote it on social media. If you have any handles or hashtags for Bluesky you would like included, please let us know.

We do not currently have any Bluesky handles or hashtags to provide.

15) Please provide a point-by-point letter INCLUDING my comments and your detailed responses (as Word file).

We have included the editor's and reviewers' comments together with our point-by-point responses in this document.

Reviewer #1:

The authors addressed all our comments satisfactorily. Consequently, the manuscript has significantly improved, and we recommend its publication.

We thank the reviewer for their careful evaluation of our manuscript and for their positive assessment of the revised work.

Reviewer #2:

The authors have fully addressed my comments on the original version. The revised version has been substantially improved.

We thank the reviewer for their careful evaluation of our manuscript and for their positive assessment of the revised work.

Reviewer #3:

I am satisfied with the resolution on all of my comments except for the one major one -- experimental demostration of the proof-of-concept as a functional sensor for e.g..
In my revised opinion, given the new form of the manuscript, I am willing to accept the lack of aforementioned demonstration to be scope-adjacent for this work and thus dont reckon it as the only reason why this work should not be published. I therefore recommend this work for publication.

We thank the reviewer for their careful evaluation of our manuscript, for their constructive feedback, and for their recommendation for publication.

25th Jun 2026
Manuscript number: MSB-2025-13399RR
Title: Engineered E. coli swarming for binary and analog input recording

Dear Dr. Danino,

Congratulations on an excellent manuscript, I am pleased to inform you that your manuscript has been accepted for publication in Molecular Systems Biology. Thank you for your comprehensive response to referee concerns. It has been a pleasure to work with you to get this to the acceptance stage.

Your manuscript will be processed for publication by EMBO Press. It will be copy edited and you will receive page proofs prior to publication. Please note that you will be contacted by Springer Nature Author Services to complete licensing and payment information.

You may qualify for financial assistance for your publication charges - either via a Springer Nature fully open access agreement or an EMBO initiative. Check your eligibility: https://link.springer.com/journal/44320/how-to-publish-with-us

Should you be planning a Press Release on your article, please notify the production team via this form in order to coordinate publication and release dates: https://forms.cloud.microsoft/e/eH4A8JVP6g

If you have any questions, please do not hesitate to contact the Editorial Office. Thank you for your contribution to Molecular Systems Biology.

Yours sincerely,

Poonam Bheda, PhD
Scientific Editor
Molecular Systems Biology

>>> Please note that it is Molecular Systems Biology policy for the transcript of the editorial process (containing referee reports and your response letter) to be published as an online supplement to each paper. If you do NOT want this, you will need to inform the Editorial Office via email immediately. More information is available here: https://link.springer.com/partners/embo-press/editorial-policies#Peer%20review

EMBO Press Author Checklist

Corresponding Author Name: Tal Danino	
Journal Submitted to: Molecular Systems Biology	
Manuscript Number: MSB-2025-13399-T	

USEFUL LINKS FOR COMPLETING THIS FORM
The EMBO Journal - Author Guidelines
EMBO Reports - Author Guidelines
Molecular Systems Biology - Author Guidelines
EMBO Molecular Medicine - Author Guidelines

Reporting Checklist for Life Science Articles (updated January
This checklist is adapted from Materials Design Analysis Reporting (MDAR) Checklist for Authors. MDAR establishes a minimum set of requirements in transparent reporting in the life sciences (see Statement of Task: 10.31222/osf.io/9sm4x). Please follow the journal's guidelines in preparing your
Please note that a copy of this checklist will be published alongside your article.

Abridged guidelines for figures
1. Data
The data shown in figures should satisfy the following conditions:
- the data were obtained and processed according to the field's best practice and are presented to reflect the results of the experiments in an accurate and unbiased manner.
- ideally, figure panels should include only measurements that are directly comparable to each other and obtained with the same assay.
- plots include clearly labeled error bars for independent experiments and sample sizes. Unless justified, error bars should not be shown for technical
- if n<5, the individual data points from each experiment should be plotted. Any statistical test employed should be justified.
- Source Data should be included to report the data underlying figures according to the guidelines set out in the authorship guidelines on Data

2. Captions
Each figure caption should contain the following information, for each panel where they are relevant:
- a specification of the experimental system investigated (eg cell line, species name).
- the assay(s) and method(s) used to carry out the reported observations and measurements.
- an explicit mention of the biological and chemical entity(ies) that are being measured.
- an explicit mention of the biological and chemical entity(ies) that are altered/varied/perturbed in a controlled manner.
- the exact sample size (n) for each experimental group/condition, given as a number, not a range;
- a description of the sample collection allowing the reader to understand whether the samples represent technical or biological replicates (including how many animals, litters, cultures, etc.).
- a statement of how many times the experiment shown was independently replicated in the laboratory.
- definitions of statistical methods and measures:
  - common tests, such as t-test (please specify whether paired vs. unpaired), simple χ2 tests, Wilcoxon and Mann-Whitney tests, can be unambiguously identified by name only, but more complex techniques should be described in the methods section;
  - are tests one-sided or two-sided?
  - are there adjustments for multiple comparisons?
  - exact statistical test results, e.g., P values = x but not P values < x;
  - definition of 'center values' as median or average;
  - definition of error bars as s.d. or s.e.m.

Please complete ALL of the questions below.
Select "Not Applicable" only when the requested information is not relevant for your study.

Materials

Newly Created Materials	Information included in the manuscript?	In which section is the information available? (Reagents and Tools Table, Materials and Methods, Figures, Data Availability Section)
New materials and reagents need to be available; do any restrictions apply?	Yes	Data Availability

Antibodies	Information included in the manuscript?	In which section is the information available? (Reagents and Tools Table, Materials and Methods, Figures, Data Availability Section)
For **antibodies** provide the following information:<br>- Commercial antibodies: RRID (if possible) or supplier name, catalogue number and or/clone number<br>- Non-commercial: RRID or citation	Not Applicable	

DNA and RNA sequences	Information included in the manuscript?	In which section is the information available? (Reagents and Tools Table, Materials and Methods, Figures, Data Availability Section)
**Short novel DNA or RNA including primers, probes**: provide the sequences.	Yes	Appendix Table S2

Cell materials	Information included in the manuscript?	In which section is the information available? (Reagents and Tools Table, Materials and Methods, Figures, Data Availability Section)
**Cell lines:** Provide species information, strain. Provide accession number in repository **OR** supplier name, catalog number, clone number, and/**OR** RRID.	Not Applicable	
**Primary cultures:** Provide species, strain, sex of origin, genetic modification status.	Not Applicable	
Report if the cell lines were recently **authenticated** (e.g., by STR profiling) and tested for mycoplasma contamination.	Not Applicable	

Experimental animals	Information included in the manuscript?	In which section is the information available? (Reagents and Tools Table, Materials and Methods, Figures, Data Availability Section)
**Laboratory animals or Model organisms:** Provide species, strain, sex, age, genetic modification status. Provide accession number in repository **OR** supplier name, catalog number, clone number, **OR** RRID.	Not Applicable	
**Animal observed in or captured from the field:** Provide species, sex, and age where possible.	Not Applicable	
Please detail **housing and husbandry conditions**.	Not Applicable	

Plants and microbes	Information included in the manuscript?	In which section is the information available? (Reagents and Tools Table, Materials and Methods, Figures, Data Availability Section)
**Plants:** provide species and strain, ecotype and cultivar where relevant, unique accession number if available, and source (including location for collected wild specimens).	Not Applicable	
**Microbes:** provide species and strain, unique accession number if available, and source.	Yes	Reagents and Tools Table, Materials and Methods

Human research participants	Information included in the manuscript?	In which section is the information available? (Reagents and Tools Table, Materials and Methods, Figures, Data Availability Section)
If collected and within the bounds of privacy constraints report on age, sex and gender or ethnicity for all study participants.	Not Applicable	

Core facilities	Information included in the manuscript?	In which section is the information available? (Reagents and Tools Table, Materials and Methods, Figures, Data Availability Section)
If your work benefited from core facilities, was their service mentioned in the acknowledgments section?	Not Applicable	

Design

Study protocol	Information included in the manuscript?	In which section is the information available? (Reagents and Tools Table, Materials and Methods, Figures, Data Availability Section)
If study protocol has been **pre-registered, provide DOI in the manuscript**. For clinical trials, provide the trial registration number **OR** cite DOI.	Not Applicable	
Report the **clinical trial registration number** (at ClinicalTrials.gov or equivalent), where applicable.	Not Applicable	

Laboratory protocol	Information included in the manuscript?	In which section is the information available? (Reagents and Tools Table, Materials and Methods, Figures, Data Availability Section)
Provide DOI OR other citation details if **external detailed step-by-step protocols** are available.	Not Applicable	

Experimental study design and statistics	Information included in the manuscript?	In which section is the information available? (Reagents and Tools Table, Materials and Methods, Figures, Data Availability Section)
Include a statement about **sample size** estimate even if no statistical methods were used.	Yes	Materials and Methods, Figure legends
Were any steps taken to minimize the effects of subjective bias when allocating animals/samples to treatment (e.g. **randomization procedure**)? If yes, have they been described?	Not Applicable	
Include a statement about **blinding** even if no blinding was done.	Yes	Materials and Methods
Describe **inclusion/exclusion criteria** if samples or animals were excluded from the analysis. Were the criteria pre-established?<br><br>If sample or data points were omitted from analysis, report if this was due to attrition or intentional exclusion and provide justification.	Yes	Materials and Methods
For every figure, are **statistical tests** justified as appropriate? Do the data meet the assumptions of the tests (e.g., normal distribution)? Describe any methods used to assess it. Is there an estimate of variation within each group of data? Is the variance similar between the groups that are being statistically compared?	Yes	Materials and Methods

Sample definition and in-laboratory replication	Information included in the manuscript?	In which section is the information available? (Reagents and Tools Table, Materials and Methods, Figures, Data Availability Section)
In the figure legends: state number of times the experiment was **replicated** in laboratory.	Yes	Figure legends
In the figure legends: define whether data describe **technical or biological replicates**.	Yes	Figure legends

Ethics

Ethics	Information included in the manuscript?	In which section is the information available? (Reagents and Tools Table, Materials and Methods, Figures, Data Availability Section)
Studies involving **human participants**: State details of **authority granting ethics approval** (IRB or equivalent committee(s), provide reference number for approval.	Not Applicable	
Studies involving **human participants**: Include a statement confirming that **informed consent** was obtained from all subjects and that the experiments conformed to the principles set out in the WMA Declaration of Helsinki and the Department of Health and Human Services Belmont Report.	Not Applicable	
Studies involving **human participants:** For publication of **patient photos**, include a statement confirming that consent to publish was obtained.	Not Applicable	
Studies involving experimental **animals**: State details of **authority granting ethics approval** (IRB or equivalent committee(s), provide reference number for approval. Include a statement of compliance with ethical regulations.	Not Applicable	
Studies involving **specimen and field samples:** State if relevant **permits** obtained, provide details of authority approving study; if none were required, explain why.	Not Applicable	

Dual Use Research of Concern (DURC)	Information included in the manuscript?	In which section is the information available? (Reagents and Tools Table, Materials and Methods, Figures, Data Availability Section)
Could your study fall under dual use research restrictions? Please check biosecurity documents and list of **select agents and toxins** (CDC): https://www.selectagents.gov/sat/list.htm	Not Applicable	
If you used a select agent, is the security level of the lab appropriate and reported in the manuscript?	Not Applicable	
If a study is subject to dual use research of concern regulations, is the name of the **authority granting approval and reference number** for the regulatory approval provided in the manuscript?	Not Applicable	

Reporting

The MDAR framework recommends adoption of discipline-specific guidelines, established and endorsed through community initiatives. Journals have their own policy about requiring specific guidelines and recommendations to complement MDAR.

Adherence to community standards	Information included in the manuscript?	In which section is the information available? (Reagents and Tools Table, Materials and Methods, Figures, Data Availability Section)
State if relevant guidelines or checklists (e.g., **ICMJE, MIBBI, ARRIVE, PRISMA**) have been followed or provided.	Not Applicable	
For **tumor marker prognostic studies,** we recommend that you follow the **REMARK** reporting guidelines (see link list at top right). See author guidelines, under 'Reporting Guidelines'. Please confirm you have followed these guidelines.	Not Applicable	
For **phase II and III randomized controlled trials**, please refer to the **CONSORT** flow diagram (see link list at top right) and submit the CONSORT checklist (see link list at top right) with your submission. See author guidelines, under 'Reporting Guidelines'. Please confirm you have submitted this list.	Not Applicable	

Data Availability

Data availability	Information included in the manuscript?	In which section is the information available? (Reagents and Tools Table, Materials and Methods, Figures, Data Availability Section)
Have **primary datasets** been deposited according to the journal's guidelines (see 'Data Deposition' section) and the respective accession numbers provided in the Data Availability Section?	Yes	Data Availability
Were **human clinical and genomic datasets** deposited in a public access-controlled repository in accordance to ethical obligations to the patients and to the applicable consent agreement?	Not Applicable	
Are **computational models** that are central and integral to a study available without restrictions in a machine-readable form? Were the relevant accession numbers or links provided?	Yes	Data Availability
If publicly available data were reused, provide the respective **data citations in the reference list**.	Yes	Materials and Methods, References
