# Supplementary material for: Engineered E. coli swarming for binary and analog input recording
Source: Mol Syst Biol. 2026 Jul 16;22(9):1377–400. doi: 10.1038/s44320-026-00232-7 (PMC13538604; doi:10.1038/s44320-026-00232-7)
Supplement: Supplementary file 8 [file 44320_2026_232_MOESM6_ESM.zip › Movie-EV2/Movie-EV2_Legend.rtf]

Movie EV2 | Time-lapse swarming of pLac-rpoS and pLac-gfp strains across IPTG concentrations. Representative raw time-lapse images of colony expansion acquired every 10 min for 24 h at 37 °C using a flatbed scanner (400 dpi; no magnification).
